# Mechanochemical Catalytic Transfer Hydrogenation of Aromatic Nitro Derivatives

**DOI:** 10.3390/molecules23123163

**Published:** 2018-11-30

**Authors:** Tomislav Portada, Davor Margetić, Vjekoslav Štrukil

**Affiliations:** Division of Organic Chemistry and Biochemistry, Ruđer Bošković Institute, Bijenička cesta 54, 10000 Zagreb, Croatia; Tomislav.Portada@irb.hr (T.P.); Davor.Margetic@irb.hr (D.M.)

**Keywords:** mechanochemistry, catalytic transfer hydrogenation, aromatic nitro derivatives, ammonium formate, aging, ball milling, synthesis

## Abstract

Mechanochemical ball milling catalytic transfer hydrogenation (CTH) of aromatic nitro compounds using readily available and cheap ammonium formate as the hydrogen source is demonstrated as a simple, facile and clean approach for the synthesis of substituted anilines and selected pharmaceutically relevant compounds. The scope of mechanochemical CTH is broad, as the reduction conditions tolerate various functionalities, for example nitro, amino, hydroxy, carbonyl, amide, urea, amino acid and heterocyclic. The presented methodology was also successfully integrated with other types of chemical reactions previously carried out mechanochemically, such as amide bond formation by coupling amines with acyl chlorides or anhydrides and click-type coupling reactions between amines and iso(thio)cyanates. In this way, we showed that active pharmaceutical ingredients Procainamide and Paracetamol could be synthesized from the respective nitro-precursors on milligram and gram scale in excellent isolated yields.

## 1. Introduction

Catalytic hydrogenation is one of the most significant functional group transformation reactions in organic synthesis and numerous procedures and reagents have been developed for that purpose [1,2]. As such, the hydrogenation reaction plays one of the key roles in many industrially important processes, for example hydrogenation of carbon monoxide to methanol or in food industry for the conversion of unsaturated vegetable oils into saturated triglycerides [3]. From a variety of available protocols, we have selected to use of formic acid salts as the source of hydrogen as a particularly appealing methodology [4,5,6]. In the presence of a catalytic amount of palladium on carbon, ammonium formate undergoes decomposition to carbon dioxide, ammonia and hydrogen [7]. This type of hydrogenation reaction is typically performed in a solvent by stirring a heterogenous mixture until the reaction is complete. Following the emergence of new greener methods for chemical synthesis, ammonium formate promoted hydrogenation has been attempted under microwave irradiation [8,9] and in ionic liquids [10]. Mechanochemistry by ball milling has attracted much attention in the chemistry community as a promising methodology that meets many of the green chemistry criteria [11,12,13,14,15]. Its effectiveness is reflected in often quantitative reaction yields and short reaction times, high product purity, low energy consumption and cost. Simple sample preparation that avoids bulk solvent and bench-top operation mode make it suitable for research and educational laboratories [16], whereas the application of twin extrusion allows large scale solid-state synthesis of compounds [17,18,19]. Novel methodological concepts, such as resonant acoustic mixing, enable an efficient solid-state synthesis of pharmaceutical cocrystals [20] while technical solutions like multiposition jars/adapters and ball milling compatible LED reactors and glass jars, assist in broadening the utility of mechanochemistry in high-throughput parallel synthesis [21] and solid-state visible light photocatalysis [22].

**Scheme 1:**
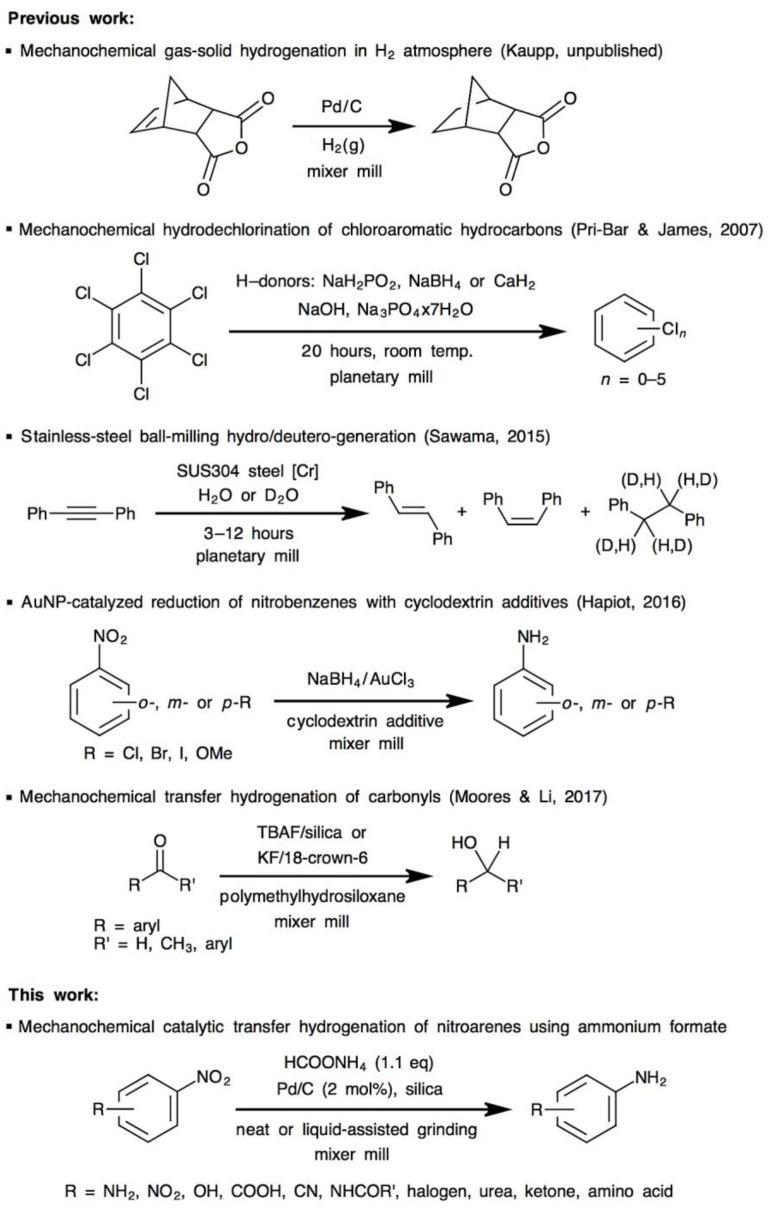
Selected recent examples of hydrogenation reactions under mechanochemical conditions.

Therefore, one can envisage that hydrogenation reaction might also be transferred into the arena of mechanochemically-assisted organic synthesis [23,24,25,26,27]. Significant progress has been made over the last several years in this respect, with mechanochemistry securing its place in organic chemistry areas like organocatalysis [28,29,30] and metal-catalysis [31], C–H functionalization [32,33], multicomponent reactions [34] and so forth. Considering the expansion of literature on utilising solid-state milling as a synthetic tool, the mechanochemical hydrogenation still remains one of the underexplored areas with only a few papers published (Scheme 1).

For example, Delogu reported hydrogenation of carbon monoxide over solid (Co_50_Fe_50_)_0.2_(TiO_2_)_99.8_ and Ni_40_(ZrO_2_)_60_ catalysts under ball milling conditions [35], while Mack et al. developed a mechanochemical approach for the hydride reduction of esters to alcohols using NaBH_4_/LiCl system [36]. An example of catalytic hydrogenation reaction using Pd/C as the catalyst under a pressure of hydrogen gas (unpublished results) was provided in a review by Kaupp [37], who described the gas-solid synthesis of norbornane-*endo*-2,3-dicarboxylic anhydride from the norbornene derivative in 200 g batches. The product was easily sublimed off from the catalyst but milling was not applicable if volatile liquids had to be constantly pumped off or if the products had become liquid or sticky. A few literature sources report on the mechanochemical reductive dehalogenation [38,39,40], for example catalytic hydrodechlorination of aromatic chlorinated compounds such as hexachlorobenzene by using calcium hydride, sodium hypophosphite or borohydride as the hydride donors in a ball mill [41]. Sawama reported on an interesting example of hydro- and deuterogenation of unsaturated substrates by H_2_O or D_2_O as H_2_ or D_2_ sources in a planetary ball mill. Chromium and nickel, as structural components of SUS304 stainless steel jars and balls used in experiments, reduce H_2_O/D_2_O generating H_2_/D_2_ in situ under rather harsh milling conditions [42,43]. In their latest report, in-situ generation of hydrogen gas from alkanes or even diethyl ether was demonstrated [44]. Hapiot et al. described gold nanoparticles-catalysed (AuNPs) reduction of several *o*-, *m*- and *p*-halogenated nitrobenzene derivatives by ball milling. With sodium borohydride as the reducing agent, cyclodextrin-stabilised AuNPs were employed as catalysts to achieve nitro-group reduction [45]. Most recently, Moores and Li employed milling for the catalytic transfer hydrogenation (CTH) of ketones and aldehydes to alcohols using polymethylhydrosiloxane with either tetrabutylammonium fluoride/silica or potassium fluoride/18-crown-6 mixtures as additives [46]. The authors demonstrated a successful reduction of the carbonyl group in the presence of potentially reactive functionalities such as alkenes or alkynes, carboxylic, cyano and nitro groups.

The reduction of aromatic nitro-group is a well-documented organic transformation [47,48,49,50,51,52,53,54]. Traditionally, the reaction is carried out in a suitable solvent under an atmosphere of hydrogen in the presence of a catalyst. Hydrogen gas can also be generated by the action of an acid (HCl or acetic acid) on metals such as zinc, iron or indium where electron transfer produces reduced radical species or indirectly from hydrogen transfer reagents (donors), for example formate salts or hydrazine. Except for the AuNP-catalysed reduction of halogenated nitrobenzenes using NaBH_4_ [45], it seems that mechanochemical approach has otherwise not been employed for the conversion of nitroaromatics to substituted anilines.

Here, we opted for easy-to-handle crystalline ammonium formate as a cheap, water soluble and low hazard hydrogen source which, in combination with Pd-C catalyst and silica as the only additive, allows for an environmentally-friendly mechanochemical catalytic transfer hydrogenation (CTH) of aromatic compounds containing the nitro functionality (Scheme 1). This method also avoids working with pressurized hydrogen gas which requires a special experimental setup and enables simple and easy-to-implement procedure in research laboratories but also in organic chemistry education as a model mechanochemical reduction reaction. Still, precautions are advised since aromatic nitro derivatives are toxic and potentially explosive.

In this paper, we demonstrate how simple milling of the reactant mixture in a ball mill provides an environment for efficient synthesis of a series of aromatic amines by CTH of nitro derivatives, particularly targeting biologically active compounds such as procainamide and paracetamol. Selected reactions have also been carried out on 1.0 g scale, whose efficiency matched the milligram scale ones.

## 2. Results and Discussion

Initially, all reactions were carried out by grinding a mixture of a nitroarene, ammonium formate and palladium on carbon (10 wt%) under solvent-free or neat grinding (NG) conditions. While CTH reactions in solution typically require 1.5–2.0 equivalents of ammonium formate as hydrogen source and extensive solvent extraction purification [55,56], solid-state milling approach allowed the loading to be decreased to 10 mol% excess with simple filtration-based work-up. However, a practical problem of diminished isolated yields was encountered in all cases due to inefficient mixing and recovery of the crude product caused by its sticky nature. For that reason, we employed silica as an inert solid support, which improved the appearance of the crude mixture to fine powder, enabling quantitative recovery of the material from the grinding jar. Due to build-up of pressure during the reaction, the jars were carefully opened in a well ventilated fume hood to allow safe release of gaseous by-products (CO_2_ and strong odour of NH_3_).

**Figure 1:**
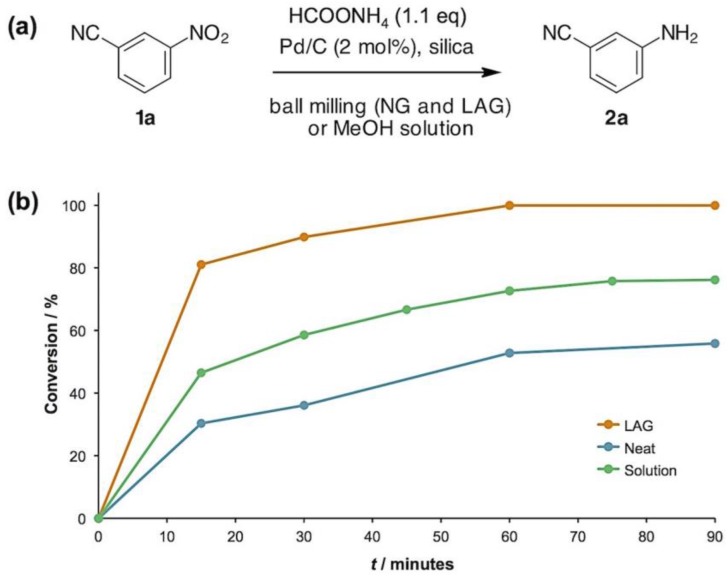
(**a**) Mechanochemical CTH model reaction; (**b**) Conversion of 3-nitro-benzonitrile during CTH determined by HPLC analysis. *Reaction conditions:* 3-nitrobenzonitrile (1.0 mmol), ammonium formate (3.3 mmol, 1.1 equivalent), 10 wt% Pd/C (21 mg, 2 mol%). In milling experiments, a 10 mL grinding jar containing one 12 mm stainless steel ball was charged with silica (175 mg) as an additive and 137 μL of methanol (LAG). The solution reaction was performed in methanol (10 mL) with stirring (1000 rpm) at 22 °C.

The advantage of performing the solid state ammonium formate promoted hydrogenation reaction is revealed in Figure 1b. The rate of 3-nitrobenzonitrile (**1a**) reduction as a model reaction (Figure 1a) in methanol (solution reaction) at room temperature using the same molar quantities of reactants as in milling reaction (1.1 eq of formate, 2 mol% of Pd/C (10 wt%)) shows partial conversion to product **2a** (ca. 70% after 90 min) monitored by HPLC. In the case of mechanochemical synthesis, the LAG reaction reaches quantitative conversion in 60 min [57]. We note that 3-nitrobenzonitrile reduction under neat grinding conditions is much less efficient in comparison with LAG reaction, reaching only ca. 55% conversion after 90 min. As previously demonstrated for mechanochemical amination of thiocarbamoyl benzotriazoles, the physico-chemical interaction of in situ formed gases and liquids used in LAG can have a profound effect on the outcome of milling reactions [58].

**Figure 2:**
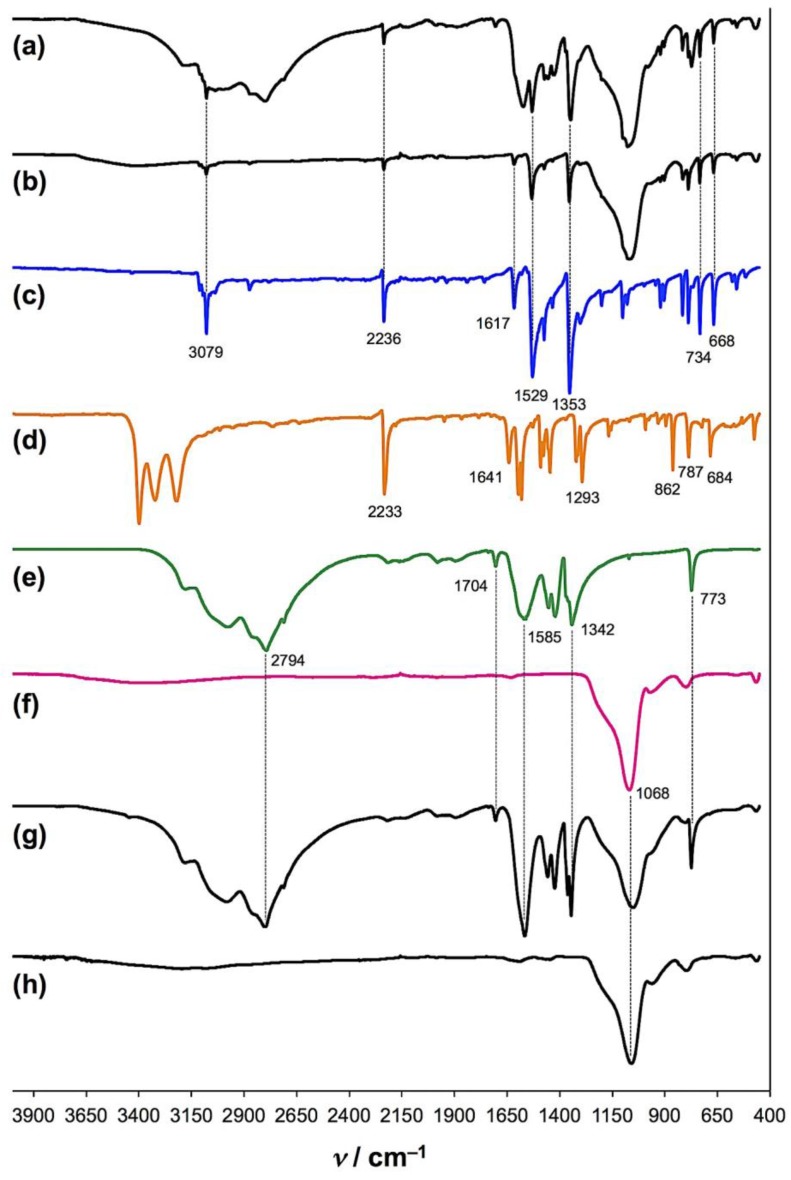
FTIR-ATR monitoring of aging reactions in open air. Reaction mixture after (**a**) gentle manual grinding for 2–3 min; (**b**) aging for 3 days; (**c**) 3-nitrobenzonitrile (**1a**); (**d**) 3-cyanoaniline (**2a**); (**e**) ammonium formate; (**f**) silica; (**g**) LAG for 60 min without substrate **1a**; (**h**) LAG-mixture under (**g**) after aging for 2 h in air.

Surprisingly, we note that the model reaction did not take place under aging conditions in open air, that is, without an extensive mechanical agitation. For example, when the reaction mixture was gently homogenized by brief manual grinding (ca. 2–3 min) in a mortar and left open at room temperature (25 °C) for 3 days, TLC, FTIR-ATR and ^1^H NMR analyses indicated complete absence of 3-cyanoaniline (**2a**) (Figure 2a–d). IR analysis also revealed quantitative decomposition of ammonium formate where only the characteristic absorption bands of the reactant **1a** (3079, 2236, 1617, 1529, 1353, 734 and 668 cm^−1^) and silica (1068 cm^−1^) were identified. Quantitative decomposition of HCOONH_4_ rapidly took place (2 h) in air when the nitro-substrate was left out. On the other hand, milling Pd/C-silica-HCOONH_4_ mixture without **1a** for 60 min resulted in partial decomposition of ammonium formate under neat or LAG conditions (ca. 20% conversion, Figure 2g) [59]. Comparison of IR spectra showed complete disappearance of the characteristic HCOONH_4_ absorption bands (2794, 1704, 1585, 1342 and 773 cm^−1^) on aging these mixtures overnight in air (Figure 2h), which is consistent with the observations in attempted aging of the model reaction. We conclude from these experiments that in open air ammonium formate undergoes Pd-catalysed decomposition to hydrogen (H_2_), carbon dioxide (CO_2_) and ammonia (NH_3_) gases, without affecting the reduction of the nitro substrate when present.

**Figure 3:**
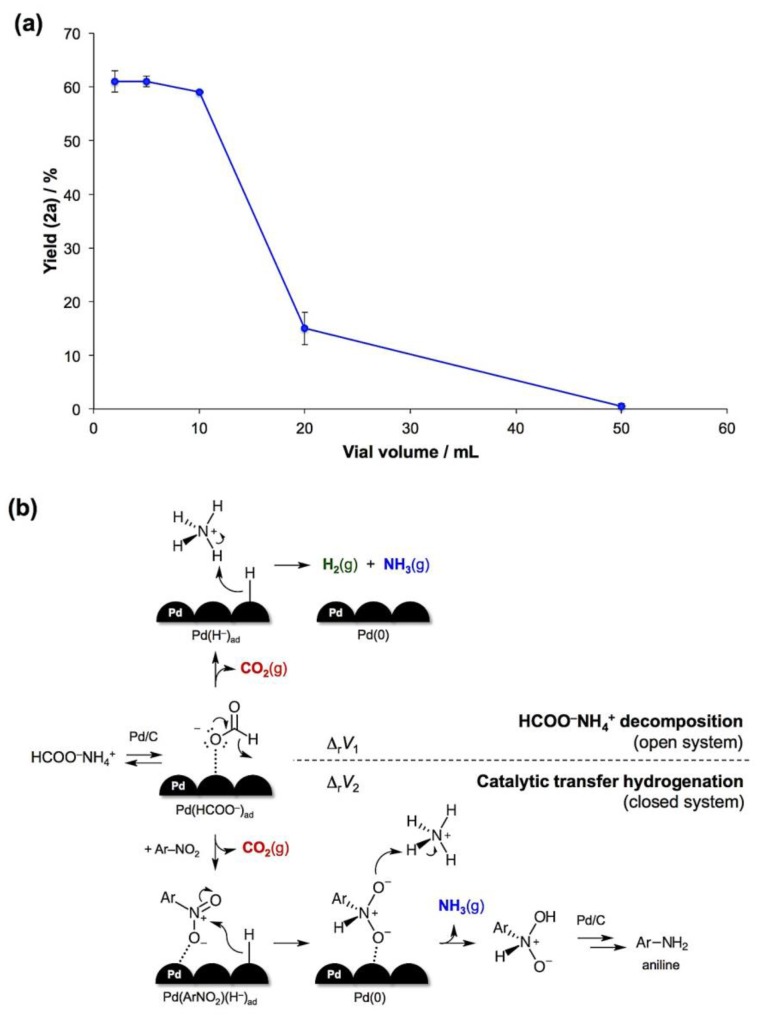
(**a**) The yield of 3-cyanoaniline (**2a**) vs. vial volume during aging reactions at room temperature; (**b**) Proposed reaction mechanism with preferred reaction paths in open and closed systems.

Next, the **1a**-Pd/C-silica-HCOONH_4_ mixture (0.5 mmol scale) was left undisturbed in a sealed 10 mL milling jar for 3 days. ^1^H NMR analysis showed 59% yield of aniline **2a**, suggesting that the reaction could take place even by aging but in a closed system. To verify this hypothesis, an experiment was designed where the model reaction mixture (0.5 mmol scale) underwent aging in glass vials of different volume, that is, 2, 5, 10, 20 and 50 mL. The 50 mL vial simulated an “open system” whereas smaller vials represented “closed systems” (Figure S2, ESI). In this way, by decreasing the reactor volume from 50 to 2 mL, the effect of internal pressure increase was achieved. After 3 days of aging, ^1^H NMR analyses confirmed that the model reaction did not proceed in the largest 50 mL vial. On contrary, the product yield in 20 mL vial averaged at 15% and levelled off at around 60% in 2, 5 and 10 mL vials (Figure 3a). The solid reaction mixtures were then left undisturbed in open air for 24 h and analysed by IR and NMR spectroscopy. As expected, IR spectra showed complete disappearance of ammonia formate absorption bands, initially present upon opening the vials (Figures S4 and S5) while ^1^H NMR revealed no or only minor changes in the composition of the reaction mixtures (Figure S3). These results indicate a strong correlation between the product yield and pressure but also provide some insight into the possible reaction mechanism outlined in Figure 3b.

Previous studies postulate the adsorption of formate ion on the surface of Pd-catalyst as the first step (Pd(HCOO^–^)_ad_) [60,61,62]. Upon release of CO_2_ molecule and formation of activated Pd-hydride species (labelled as Pd(H^–^)_ad_), ammonium cations acting as Brønsted acid protonate these hydride species, generating hydrogen and ammonia gases. Alternatively, substrate molecules compete with NH_4_^+^ ions for Pd(H^–^)_ad_ sites and following the hydride transfer to NO_2_-group, the resulting intermediate is protonated by NH_4_^+^ with the release of NH_3_ molecule. These species then re-enter the catalytic cycle and eventually afford the aniline product (which explains the required substrate:donor = 1:3 stoichiometry). While Pd-catalysed HCOONH_4_ decomposition generates 3 equivalents of gaseous products (H_2_, CO_2_ and NH_3_) per 1 equivalent of the salt, during CTH reaction 2 equivalents are released (CO_2_ and NH_3_). The respective reaction volume changes are denoted as Δ_r_*V*_1_ and Δ_r_*V*_2_, whereas Δ_r_*V*_1_ > Δ_r_*V*_2_ [63]. This implies that the former reaction (Δ_r_*V*_1_) is more susceptible to pressure changes than the latter one (Δ_r_*V*_2_). In particular, in accordance with Le Chatelier’s principle an increase in the internal pressure should suppress the reaction with larger Δ_r_*V*, in our case ammonium formate decomposition. However, this reaction pathway is preferred in an open system (large reactor volumes or completely exposed to air) where gases freely escape the reaction mixture and due to irreversibility of this process, the reduction of NO_2_-group does not occur. In a closed system, the preferred reaction path would be CTH as it is less sensitive to higher internal pressure. The competition between these two possible reaction channels is best reflected in higher conversion of **1a** and increased yield of **2a** on reducing the reactor volume (Figure 3a). When the **1a**-Pd/C-silica-HCOONH_4_ mixture (0.5 mmol scale) was briefly ball milled in a 10 mL jar for only 2 min, prior to aging for 3 days at room temperature, the yield of **2a** reached 88%. This also illustrates the importance of ball milling for the efficiency of CTH since the introduction of mechanical agitation into the aging system alleviates the 60% yield plateau and facilitates effective mixing, mass transfer and fresh surface exposure, thus allowing for a quantitative synthesis of **2a** and a number of other aromatic derivatives in 90 min (Table 1). Finally, the dual reactivity was exploited in the isolation procedure where a slight excess of HCOONH_4_ is efficiently removed from the crude reaction mixture by allowing it to stand in air overnight and decompose to gaseous by-products.

**Figure 4:**
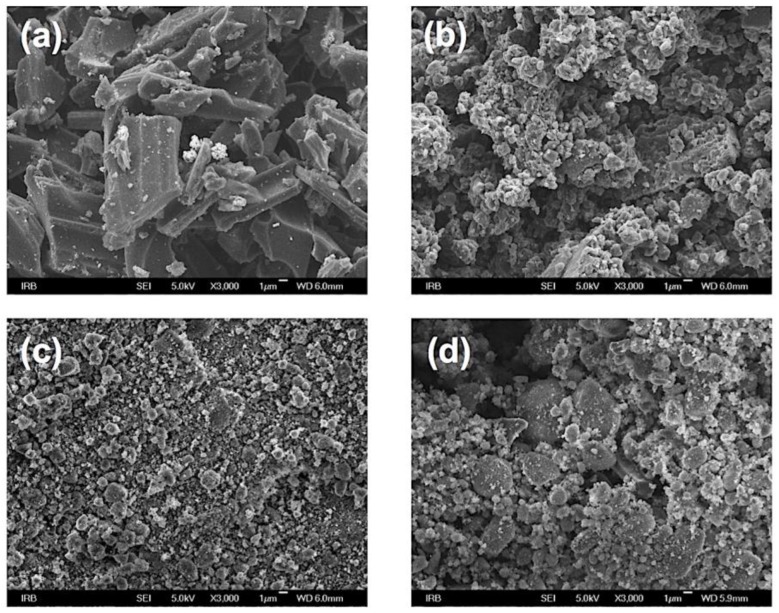
SEM images (3000×) of (**a**) commercial and (**b**) milled samples of 10 wt% Pd/C catalyst; (**c**) The catalyst milled with silica under LAG conditions for 60 min and (**d**) post-workup sample after CTH of 3-nitrobenzonitrile.

In the studied system, an important consideration is the effect of milling on the particle size and their morphology. Samples of commercial and milled Pd/C catalyst, as well as the catalyst milled in the presence of silica were analysed by scanning electron microscopy (SEM) as shown in Figure 4. Energy-dispersive X-ray spectroscopy (EDS) analysis indicates that palladium is organized into aggregates of different sizes adsorbed on the surface of carbon particles (Figures S6 and S8). On milling for 60 min, their size and morphology drastically change and the sample becomes more homogeneous with palladium distribution more uniform as suggested by EDS analysis (Figures S7 and S9). Similar effect is observed in Pd/C milled with silica where the average particle size achieved after 60 min of LAG (methanol, 12 mm ball size, 30 Hz) is estimated at ca. 0.1–1.0 μm. Analysis of the catalyst milled with silica under solvent-free conditions reveals similar average particle size range, suggesting that LAG has no influence on particle size in this system.

Following the initial studies on the reduction of 3-nitrobenzonitrile (**1a**) to 3-cyanoaniline (**2a**), we focused our efforts on investigating the scope of mechanochemical CTH reaction (Table 1). Simple nitroaromatic derivatives such as *o*-, *m*- and *p*-nitroanilines and phenols gave the respective phenylenediamines (***o*-**, ***m*-** and ***p*-2b**) and aminophenols (***o*-**, ***m*-** and ***p*-2c**) in almost quantitative yields. 1,2-, 1,3- and 1,4-dinitrobenzenes also smoothly underwent the reduction and led to excellent isolated yields of phenylenediamines (***o*-**, ***m*-** and ***p*-2b**). The same results were obtained with *o*-, *m*- and *p*-nitrobenzoic acids, 3,5-dinitrobenzoic acid and 2-nitroterephthalic acid as the carboxylic group containing substrates, which all efficiently gave aminobenzoic acids **2d–f**. Next, we wanted to investigate the compatibility of our mechanochemical approach with different protecting groups, for example acetyl, ethyl carbamoyl, *tert*-butoxycarbonyl (Boc) and 9-fluorenylmethyloxycarbonyl (Fmoc). Gratifyingly, in all cases the protecting group survived the reduction conditions and afforded substituted anilines **2g–j** in high yields. In the case of amino acid derivatives, the Boc-protection in alanine compound **2k**, as well as Fmoc-protection present in phenylalanine compound **2l-Phe** and valine product **2l-Val** did not interfere with the reduction of the nitro group which proceeded in a straightforward and clean fashion. This result is in contrast to the reported Fmoc-deprotection of amino acids which was accomplished under relatively mild conditions by hydrogenation (1 atm, baloon) in methanol solution using 10 wt% of Pd/C catalyst [64]. 4-Nitrophthalimide (**1m**), as a model compound containing the imide functionality, was also quantitatively converted to 4-amino-phthalimide (**2m**).

Expectedly, *N*-(3-nitrophenyl)-*N’*-phenylthiourea (**1n-S**) did not react as it is known from literature that sulphur is not compatible with this type of hydrogenation reaction due to catalyst poisoning [65,66]. On the other hand, the presence of an oxygen atom in the analogous *N*-(3-nitrophenyl)-*N’*-phenylurea (**1n-O**) did not affect the catalyst activity and led to an excellent 96% isolated yield of aminophenylurea **2n-O** in 90 min. 3-Nitroacetophenone **1o** only partially reacted under standard 1.0 mmol scale conditions. This reduction resulted in 11% conversion to the product **2o** in 90 min but increasing the ball-to-reactant ratio [67] from 10.6 to 21.1 by lowering the scale to 0.5 mmol, as well as increasing the HCOONH_4_ and Pd/C loadings to 2.2 eq and 5 mol%, proved beneficial providing 3-aminoacetophenone (**2o**) in 95% isolated yield after 6 h milling.

In the case of halogen-substituted nitroarenes, for example 2-chloro-4-nitroaniline (***p*-1p**) and 4-chloro-2-nitroaniline (***o*-1p**), CTH with 1.1 eq of ammonium formate gave a mixture of the hydrogenation and dehalogenation products after 90 min of LAG, as established by GC analysis. Following chromatographic separation, 2-chloro-1,4-phenylenediamine (***p*-2p**) (17%) and 4-chloro-1,2-phenylenediamine (***o*-2p**) (42%) were isolated. Interestingly, MS analysis of polar fractions (MeOH as eluent) from the reduction of 4-chloro-2-nitroaniline (***o*-1p**) indicated the presence of compounds with *m*/*z* values 212 and 246, which correspond to 2,2′-diaminoazobenzene and 2,2′-diamino-4-chloroazobenzene, respectively (Figure S10, ESI). According to the generally accepted mechanism of NO_2_ to NH_2_ reduction [51,53], azobenzene species have been proposed as reactive intermediates, implying that in our case the CTH reaction was not complete despite almost quantitative conversion of the starting nitro derivatives ***p*-1p** and ***o*-1p**, as shown by GC analyses (Figures S11–S13). This is the result of HCOONH_4_ being consumed for the cleavage of carbon-chlorine bond, which disrupts the stoichiometry required for a quantitative reduction to the amino product. Unfortunately, increasing the amount of the hydrogen donor to 2.2 eq (6.6 mmol) or catalyst loading to 5.0 mol% again led to dehalogenation as the primary reaction pathway. Similarly, a selective NO_2_-reduction in the presence of *O*-benzyl protecting group in compound **1q** failed with only 37% yield of the desired amino-derivative **2q** after chromatographic purification. Here, the cleavage of O–Bn bond competes with the reduction of NO_2_ group leading to a mixture of unreacted **1q**, **2q**, ***p*-1c** and ***p*-2c** (4-nitro- and 4-aminophenol).

As examples of α,β-unsaturated carbonyl compounds, *m*- and *p*-nitrochalcones ***m*-1r** and ***p*-1r** were subjected to mechanochemical reduction. While the respective aminochalcones ***m*-2r** and ***p*-2r** were isolated in quantitative yields, we did not observe reduction of the carbonyl groups in these reactions, which makes the proposed synthetic protocol complementary to the recently published CTH of aldehydes and ketones [46]. The quantitative conversion was also observed for 4-nitrotoluene (**1s**) which gave 95% of *p*-toluidine (**2s**) after isolation. CTH of the corresponding nitro substrates **1t**, **1u** and **1v** efficiently afforded 5-aminoquinoline (**2t**), 5-amino-4-methylquinoline (**2u**) and 8-aminoquinoline (**2v**) as representatives of pyridine-fused nitroaromatics.

The most challenging substrates were found to be polyaromatic nitro derivatives, for example 1-nitronaphthalene (**1x**), 9-nitroanthracene (**1y**) and 1-nitropyrene (**1z**) where GC analysis showed only traces of 9-aminoanthracene after 6 h of milling and no reaction with the pyrene compound. Naphthalene derivative **1x** displayed some reactivity based on NMR analysis of the crude reaction mixture. Under standard conditions (1.0 mmol, 90 min), 18% of 1-aminonaphthalene (**2x**) was obtained while prolonging the reaction time to 6 h increased the yield to 37% [68]. Interestingly, 6-nitrotetralin (**1w**) was more reactive and afforded 40% of 6-aminotetralin (**2w**) after 90 min. Still, quantitative conversion to **2w** required 2.2 eq of HCOONH_4_ (6.6 mmol), 5 mol% of Pd/C and 3 h of milling.

**Scheme 2:**
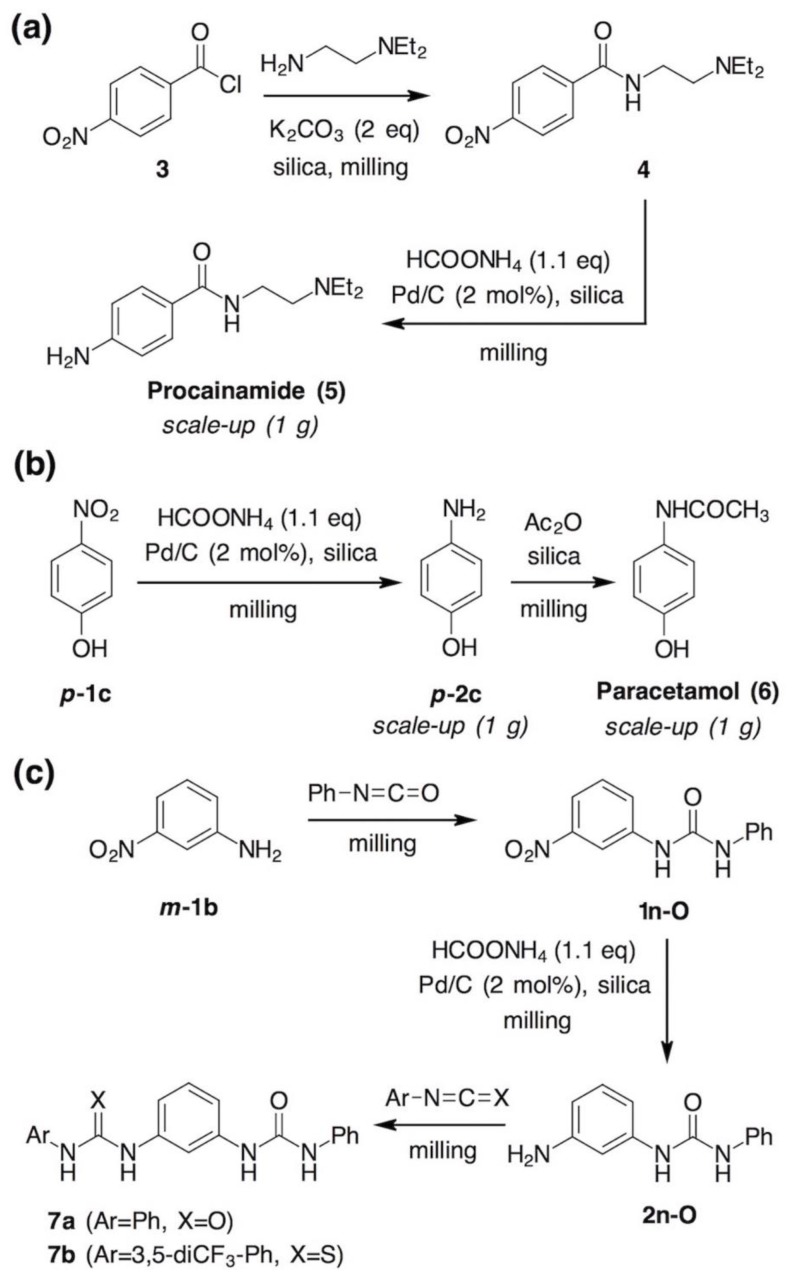
Two-step mechanochemical synthesis of active pharmaceutical ingredients (**a**) procainamide (**5**); (**b**) paracetamol (**6**) by coupling mechanochemical nitro-group reduction, amide formation and acetylation; (**c**) Integration of CTH with amine-iso(thio)cyanate coupling for the mechanochemical three-step synthesis of bis (thio)ureas **7a–b**. For details, see Supplementary Materials.

Finally, we wanted to demonstrate the utility of mechanochemical CTH reaction in the multi-step milling syntheses of two active pharmaceutical ingredients (API) and two structural analogues of urea-type anion sensors. The first example describes a successful two-step preparation of an API procainamide (**5**). Procainamide is classified as a sodium channel blocker used in treatment of cardiac arrhythmia, administered orally or intravenously [69]. Previously, there have been several attempts at utilizing mechanochemistry for the synthesis of biologically active compounds and APIs [70], for example teriflunomide [71], Leu-enkephalin [72], atorvastatin [73] and several sulfonylureas [74]. Here we wish to extend the list of useful marketed drugs amenable to mechanochemical milling synthesis (Scheme 2).

Excellent conversion of 4-nitrobenzoic acid to 4-aminobenzoic acid (***p*-2d**) was further explored in CTH reaction of 4-nitrobenzoyldiethylethylenediamine (**4**) which is a precursor for procainamide on the industrial scale synthesis. A two-step procedure, consisting of amide coupling followed by catalytic transfer hydrogenation, was employed to prepare **5** by solid state ball milling. The formation of amide **4** in the first step was accomplished through mechanochemical K_2_CO_3_–assisted coupling of solid 4-nitrobenzoylchloride (**3**) (1.1 eq) with *N*,*N*-diethylethylenediamine in the presence of silica as the milling auxiliary [75,76]. Simple extraction with ethyl acetate furnished the pure nitro intermediate **4** in 88% yield. In the next step, **4** was subjected to CTH under LAG conditions using methanol to afford procainamide (**5**) quantitatively in 90 min (Scheme 2a). As in previous examples, the crude reaction mixture was suspended in methanol, followed by filtration and evaporation of the solvent, yielding the target drug **5**. The CTH of nitro-precursor **4** can be scaled-up to 5.0 mmol, affording 1.18 g of procainamide (**5**) in quantitative yield after 6 h of milling.

Furthermore, 4-aminophenol (***p*-2c**) obtained by mechanochemical hydrogenation of *p*-nitrophenol (***p*-1c**) was successfully utilized for the rapid and quantitative solvent-free synthesis of paracetamol (**6**), a widely used analgesic and antipyretic active pharmaceutical ingredient (API) (Scheme 2b). Acetylation of the amino group was in this case accomplished by milling an equimolar mixture of ***p*-2c** and acetic acid anhydride for 30 min with silica as the milling auxiliary. To avoid using stainless steel in a corrosive environment and also potential contamination of the sample with iron by the action of acetic acid, all experiments were carried out in Teflon jars charged with a single 10 mm Teflon ball. The product was separated from silica by simply suspending the crude mixture in methanol, filtration and evaporation of the filtrate. To the best of our knowledge, this is the first example of paracetamol synthesis aided by milling mechanochemistry. NMR and IR analyses of the product **6** confirmed quantitative reaction and exclusive acetylation at the nitrogen atom. Each step (hydrogenation and acetylation) was also done on a gram scale producing ***p*-2c** (10 mmol, 1.09 g) and **6** (6.6 mmol, 0.99 g) in near quantitative isolated yields (99%). Paracetamol is known to crystallise in three polymorphic forms (form I, II and III) with distinctive physical properties important to pharmaceutical application [77,78,79]. Powder X-ray diffraction (PXRD) analysis showed that paracetamol synthesised mechanochemically by this route adopts the structure of form I (CSD refcode HXACAN01), which is the thermodynamically most stable polymorph (Figure S36, ESI).

In our previous studies, the solid state ball milling approach was established as the superior method for the synthesis of (thio)ureas by coupling amines with iso(thio)cyanates [80,81]. Particularly interesting feature of this method is the ability to quantitatively desymmetrise 1,2-phenylenediamine (***o*-2b**) by reacting it with an equimolar amount of iso(thio)cyanate electrophile [82]. Sterically less demanding 1,4-phenylenediamine (***p*-2b**) proved to be much more difficult substrate in this respect, resulting in a successful desymmetrisation reaction only with less reactive 4-methoxyphenyl isothiocyanate. For the same reason, milling 1,3-phenylenediamine (***m*-2b**) with highly reactive phenyl isocyanate failed to selectively afford aminourea **2n-O** (21%) [83]. With the optimised mechanochemical CTH reaction conditions in hands, we now envisaged a three-step route to bis(urea) and hybrid urea-thiourea derivatives **7a,b** based on a 1,3-phenylene spacer group by integrating the solid state click-type amine-isocyanate coupling with the mechanochemical CTH of nitroarenes as the key "indirect" desymmetrization step (Scheme 2c). This approach produced the desymmetrized amino-urea **2n-O** in 88% yield (over two steps), which was then in the third step successfully transformed into bis(urea) **7a** (98%). Also, the introduction of a privileged 3,5-di(trifluoromethyl)phenyl structural motif in **7b** was accomplished in 93% yield, resulting in isolated yields for **7a,b** over three steps of 87% and 82%, respectively.

## 3. Materials and Methods 

In a typical experiment, a mixture of nitroarene (1.0 mmol), ammonium formate (3.3 mmol, 208 mg, 1.1 equivalent based on the donor:substrate = 3:1 stoichiometry required for a quantitative reduction of the NO_2_ to NH_2_ functional group), 10 wt% palladium on carbon (21.0 mg, 2 mol%) and silica (175 mg) was loaded into a 10 mL stainless steel grinding jar along with one 12 mm diameter grinding ball. The mixture was ground in the presence of methanol as the grinding liquid (*η* = 0.25 μL mg^−1^) for 90 min, unless otherwise stated (see Table 1). The crude product was scraped off the walls of the jar, left in air overnight to allow excess ammonium formate to completely decompose and suspended in 10 mL of methanol. The suspension was filtered over a Büchner funnel and the filtrate was evaporated to afford amino derivatives. If necessary, the products were purified by column chromatography. Experimental details, spectroscopic and electron microscopy characterization data can be found in the Supplementary Materials.

## 4. Conclusions

We have successfully demonstrated that an efficient catalytic transfer hydrogenation of aromatic nitro compounds using readily available ammonium formate as hydrogen donor and palladium on carbon as the catalyst can be accomplished under solid-state mechanochemical milling conditions. The synthetic protocol, which is very simple and straightforward, tolerates a variety of functional groups, including amino, hydroxyl, carboxyl, carbamate, amide, imide, urea, acetyl, α,β-unsaturated carbonyl and fused heterocycles such as pyridine. The presence of halogen or *O*-benzyl substituents results in diminished yields, while polyaromatic substrates are not compatible. The mechanochemical approach has also been applied for the two-step synthesis of pharmaceutically important procainamide and paracetamol drugs, both on milligram and gram scale. We have also shown that the mechanochemical CTH can be coupled with the previously developed concept of click-mechanochemistry, to enable a multi-step synthesis of (thio)urea hybrid molecules as potential scaffolds in anion sensor and organocatalyst design.

## Figures and Tables

**Table 1:** The scope of mechanochemical catalytic transfer hydrogenation ^1^.

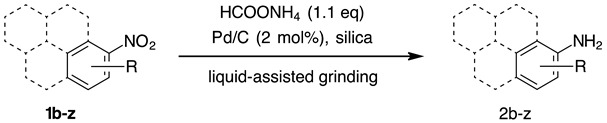			
Product	Yield ^2^	Product	Yield
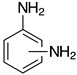	***o*-2b**, 99% (99%) ***m*-2b**, 98% (96%) ***p*-2b**, 95% (96%)	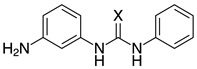	**2n-S**, n.r. (X=S) **2n-O**, 96% (X=O)
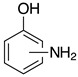	***o*-2c**, 99% ***m*-2c**, 94% ***p*-2c**, 99%	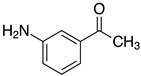	**2o**, 95% ^3^
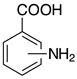	***o*-2d**, 98% ***m*-2d**, 99% ***p*-2d**, 97%	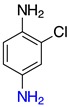	***p*-2p**, 17%
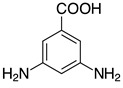	**2e**, 99%	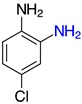	***o*-2p**, 42%
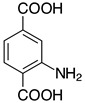	**2f**, 99%	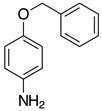	**2q**, 37%
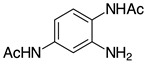	**2g**, 94%	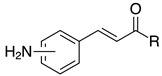	***m*-2r**, 99% (R=Ph) ***p*-2r**, 99% (R=Ph)
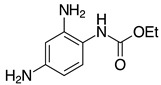	**2h**, 93%	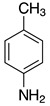	**2s**, 95%
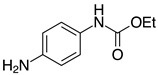	**2i**, 99%	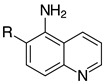	**2t**, 95% (R=H) **2u**, 84% (R=Me)
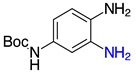	**2j**, 99%	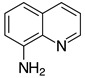	**2v**, 90%
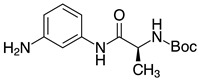	**2k**, 99%	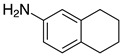	**2w**, 40% (97%) ^3^
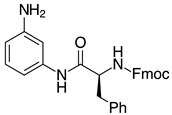	**2l-Phe**, 93% ^3^	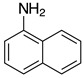	**2x**, 18% (37%)
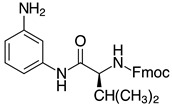	**2l-Val**, 90% ^3^	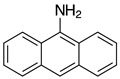	**2y**, traces
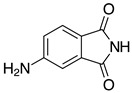	**2m**, 97%	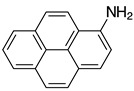	**2z**, n.r.

^1^ Reaction conditions: 1.0 mmol substrate, 3.3 mmol HCOONH_4_, 2 mol% of Pd/C and 175 mg of silica were ground in a 10 mL jar using one 12 mm stainless steel ball at 30 Hz for 90 min. Methanol was used for LAG (*η* = 0.25 μL mg^−1^). ^2^ For **2b** isomers, yields in the parentheses refer to CTH of the respective dinitrobenzenes on 0.75 mmol scale. ^3^ For compounds **2l-Phe**, **2l-Val**, **2o** and **2w**, 2.2 eq of HCOONH_4_ and 5 mol% of Pd/C were required, while for **2j**, ***p*-2p** and ***o*-2p**, blue colour indicates the position of the reduced nitro group. Other details can be found in the Supplementary Materials.

## Supplementary Materials

The following are available online at http://www.mdpi.com/1420-3049/23/12/3163/s1. General procedure for the mechanochemical reduction of nitroarenes, spectroscopic data for compounds **2a**–**x** and **4**–**7**, catalytic transfer hydrogenation of 3-nitrobenzonitrile under aging conditions, SEM and EDS analysis of the commercial and milled Pd/C catalyst samples, MS and GC analysis of CTH of 4-chloro-2-nitroaniline (***o*-1p**) and 2-chloro-4-nitroaniline (***p*-1p**), ^1^H and ^13^C NMR spectra of selected compounds and PXRD analysis of mechanochemically-synthesized paracetamol (**6**). Figure S1: ^1^H NMR spectrum of the crude reaction mixture after 6 h of milling under standard conditions (1.0 mmol scale, 1.1 eq HCOONH_4_, 2 mol% Pd/C) for the catalytic transfer hydrogenation of 1-nitronaphthalene (**1x**) to 1-aminonaphthalene (**2x**). Integration of the corresponding signals revealed the ratio **1x**:**2x** = 63:37, Figure S2: Aging experiments in vials of different volume. Each vial contained a mixture of 0.5 mmol of 3-nitrobenzonitrile (**1a**), 1.65 mmol of HCOONH_4_ (104 mg, 1.1 eq), 2 mol% of Pd/C catalyst (10.5 mg) and 87.5 mg of silica. This mixture was prepared by gentle manual grinding of **1a**, Pd/C and silica in a mortar, the resulting fine powder was then transferred to a vial, pre-ground ammonium formate was stirred in and quickly sealed. The final mixture was gently shaken in the vial for ca. 10 s and left undisturbed for 3 days, Figure S3: Representative ^1^H NMR spectra of the mixture taken from the 10 mL vial immediately after opening and 24 h in air. The spectra were recorded in CDCl_3_. Notably, there is no significant change in the amount of 3-cyanoaniline (**2a**), confirming that upon exposure to air ("open system") the NO_2_-reduction stops and the primary reaction path becomes Pd-catalysed decomposition of HCOONH_4_, Figure S4: Representative IR spectra of the aging mixture from 10 mL vial immediately after opening, 4 h and 24 h in air. Ammonium formate decomposes in air revealing the absorption bands of **1a**, **2a** and silica after 24 h, Figure S5: Representative IR spectra of the aging mixture from 20 mL vial immediately after opening, 4 h and 24 h in air. Ammonium formate decomposes in air revealing the absorption bands of **1a** and silica after 24 h, Figure S6: SEM analysis of commercial Pd/C catalyst (10 wt%). (a–c) Typical morphology of carbon particles (ca. 10–100 μm) with palladium aggregates (shown in light grey to white) of different sizes (from ca. 100 nm to several μm) distributed on carbon surface (500× and 5000×). (d) A close-up view of one Pd aggregate (50,000×). Each aggregate is composed of many nanometre-sized Pd particles, Figure S7: SEM analysis of commercial Pd/C catalyst (10 wt%) milled for 60 min at 30 Hz using a single 12 mm (7.0 g) stainless steel ball. Morphology of the samples a–d (500×–10,000×) is characteristic for homogenization and pulverization of carbon particles during ball milling resulting in particle sizes ca. 0.5–5 μm, Figure S8: SEM-EDS analysis of commercial palladium on carbon (10 wt%). A distinctive feature of the catalyst as determined by SEM is the organization of palladium into aggregates (seen as small light particles) on the surface of larger carbon particles. (**a**) Analysis of the selected area on a large carbon particle shows traces of Pd, (**b**) point analysis on the same carbon particle identifies the palladium aggregate, (**c**) analysis of the selected area comprising both carbon particles and Pd aggregates, Figure S9: SEM-EDS analysis of (**a**) commercial Pd/C catalyst (10 wt%) milled for 60 min at 30 Hz using a single 12 mm (7.0 g) stainless steel ball shows a homogeneous distribution of Pd over the entire selected area, (**b**) Pd/C catalyst milled in the presence of silica under LAG conditions for 60 min, (**c**) a post-workup sample of the mixture containing Pd/C and silica after LAG reduction of **1a** to **2a** for 60 min. The intensity of Pd signal in EDS spectra under (**b**) and (**c**) is reduced because of dilution effect by added silica. All samples contain iron contamination (Fe*) due to abrasion of stainless steel jar walls and the ball during milling, Figure S10: MS analysis of the methanol fraction after CTH of 4-chloro-2-nitroaniline (***o*-1p**) under standard conditions. 2,2′-Diaminoazobenzene (*M*_r_ = 212) and 2,2′-diamino-4-chloroazobenzene (*M*_r_ = 246) were identified as intermediates in the reaction mixture, which was consistent with the observed diminished yield (42%) of 4-chloro-*o*-phenylenediamine (***o*-2p**) due to dehalogenation side reaction, Figure S11: GC analysis of the crude mixture after CTH of 4-chloro-2-nitroaniline (***o*-1p**). *o*-Phenylenediamine (*t*_R_ = 4.18 min) and *o*-nitroaniline (*t*_R_ = 5.06 min) are the by-products of dehalogenation side reaction. The reactant was consumed completely while the signal at *t*_R_ = 5.22 min corresponds to 4-chloro-*o*-phenylenediamine (***o*-2p**) product, Figure S12: GC analysis of the crude mixture after CTH of 2-chloro-4-nitroaniline (***p*-1p**). *p*-Phenylenediamine (*t*_R_ = 4.41 min) and *p*-nitroaniline (*t*_R_ = 5.77 min) are the by-products of dehalogenation side reaction. The reactant (*t*_R_ = 6.06 min) was not consumed completely while the signal at *t*_R_ = 5.16 min corresponds to 2-chloro-*p*-phenylenediamine (***p*-2p**) product, Figure S13: GC analysis of the crude mixture after CTH of 2-chloro-4-nitroaniline (***p*-1p**) with 2.2 eq of ammonium formate (6.6 mmol). *p*-Phenylenediamine (*t*_R_ = 4.41 min) and small amount of 2-chloro-*p*-phenylenediamine (***p*-2p**) were found as the only products, suggesting that dehalogenation became the primary reaction pathway, Figures S14–35: ^1^H and ^13^C NMR spectra of **2g**–**2r**, **2t**–**2w**, **5** and **7a**–**b**, Figure S36: PXRD analysis shows that mechanochemically-synthesized paracetamol (**6**) adopts the crystal structure of the thermodynamically most stable polymorph, form I.
