# Supplementary material for: Mechanochemical Catalytic Transfer Hydrogenation of Aromatic Nitro Derivatives
Source: Molecules. 2018 Nov 30;23(12):3163. doi: 10.3390/molecules23123163 (PMC6321105; doi:10.3390/molecules23123163)
Supplement: Supplementary file 1 [file molecules-23-03163-s001.pdf]

Supplementary Materials for

Mechanochemical catalytic transfer hydrogenation of aromatic nitro derivatives

Tomislav Portada, Davor Margetić and Vjekoslav Štrukil*

vstrukil@irb.hr

Division of Organic Chemistry and Biochemistry, Ruđer Bošković Institute, Bijenička cesta 54, 10000 Zagreb, Croatia

Table of contents

1. General comments S2

2. General procedure for the mechanochemical reduction of nitroarenes S2

3. Spectroscopic data for compounds **2a-x** and **4-7** S3

4. Catalytic transfer hydrogenation of 3-nitrobenzonitrile under aging conditions S14

5. SEM and EDS analysis of the commercial and milled Pd/C catalyst samples S17

6. MS and GC analysis of CTH of 4-chloro-2-nitroaniline (***o*-1p**) and 2-chloro-4-nitroaniline (***p*-1p**) S20

7. ^{1}H and ^{13}C NMR spectra of selected compounds S22

8. PXRD analysis of mechanochemically-synthesized paracetamol (**6**) S44

9. References S45

1. General comments

All chemicals were purchased from commercial sources (Sigma Aldrich, Alfa Aesar, Kemika) and were used as received. Small-scale experiments were carried out in a Retsch MM200 mill at frequency of 30 Hz using a 10 mL stainless steel grinding jar and a single stainless steel ball of 12 mm diameter. For scale-up experiments, a 25 mL jar charged with two 12 mm balls was used. Dry methanol was used as the liquid phase throughout all liquid-assisted grinding (LAG) experiments and Merck Silica 60 (35-75 μm) was used as the milling auxiliary. ^{1}H and ^{13}C NMR spectra were recorded on a Brucker Avance 300 or 600 MHz spectrometer, with tetramethylsilane as an internal standard. Proton chemical shifts were referenced to tetramethylsilane (TMS) as an internal standard. Thin-layer chromatography was performed on TLC Silica gel 60 F254 plates. For column chromatography, Merck Silica gel 60 (63-200 μm) was used. FTIR-ATR spectra were recorded using a Fourier-Transform Infrared Attenuated Total Reflection PerkinElmer UATR Two spectrometer in the range 450 cm^{-1} to 4000 cm^{-1}. HRMS analysis for compounds **2g-x**, **4**, **5** and **7** was performed on MALDI TOF/TOF 4800 Plus analyzer. Quantitative HPLC analysis was done on a Varian Prostar instrument equipped with Restek Ultra IBD column (25 x 4.6 mm) and eluted with 2% acetic acid : methanol = 50 : 50 at 254 nm and a flow rate 1.0 mL min^{-1}. The temperature was set to 22 °C. A methanol solution of *N*,*N'*-diphenylcyanoguanidine ($c = 8\cdot10^{-4}$ mol dm^{-3}) was used as an internal standard. For the analysis of mechanochemical reactions, 2.0–3.0 mg of the crude mixture was suspended in 1 : 1 mixture of methanol (500 μL) and the internal standard solution (500 μL, total volume 1000 μL). An aliquot of 5 μL was injected and the conversion of 3-nitrobenzonitrile (**1a**) to 3-aminobenzonitrile (**2a**) was calculated from the relative ratio of areas under the corresponding signals. In the case of solution reaction, a 10 μL sample was diluted in 1 : 1 mixture of methanol (500 μL) and the internal standard solution (500 μL) and analyzed as described above. Scanning electron microscopy (SEM) images and energy-dispersive X-ray spectroscopy (EDS) data for Pd/C catalyst were aquired on a JEOL JSM-7000F microscope.

2. General procedure for the mechanochemical reduction of nitroarenes

In a typical experiment, a mixture of nitroarene compound (1.0 mmol), ammonium formate (3.3 mmol, 208 mg, 1.1 equivalent), palladium catalyst (10 % Pd on activated carbon, 2 mol%, 21 mg) and silica (175 mg) was ball milled in the presence of dry methanol (η = 0.25 μL mg^{-1}) for 90 minutes. After milling, a small sample (≈ 1 mg) of the crude reaction mixture was suspended in methanol and immediately analyzed by TLC (typically using dichloromethane : methanol = 20 : 1 mixture as an eluent). The crude mixture was left in a well ventilated hood overnight, suspended in methanol and filtered over a Büchner funnel. Evaporation of the filtrate afforded the desired amino-derivative. If necessary, the final product was purified by column chromatography.

3. Spectroscopic data for compounds 2a-x and 4-7

Note: Compounds **2a-f** (3-aminobenzonitrile, phenylenediamines, aminophenols and aminobenzoic acids), **2s** (*p*-toluidine) and **6** (paracetamol) were identified by comparison of their NMR and IR spectroscopic data with the ones available online (https://www.sigmaaldrich.com/european-export.html and https://sdbs.db.aist.go.jp/sdbs/cgi-bin/direct_frame_top.cgi)[1] or in literature sources.[2]

3-aminobenzonitrile (2a): 117 mg (99 %), ^{1}H NMR (d_6-DMSO, 300 MHz) δ/ppm = 7.19 (m, 1 H, arom.), 6.86 (m, 3 H, arom.), 5.60 (s, 2 H, NH_2); ^{13}C NMR (d_6-DMSO, 75 MHz) δ/ppm = 149.5, 130.1, 119.5, 118.7, 118.4, 115.7, 111.4; FTIR-ATR ν/cm^{-1} = 3399, 3323, 3221, 2233, 1641, 1597, 1581, 1491, 1446, 1322, 1293, 862, 787, 684, 475.

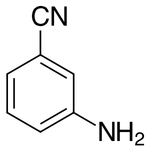

***o*-phenylenediamine (*o*-2b):**

From 2-nitroaniline: 107 mg (99 %), *from 1,2-dinitrobenzene (0.75 mmol scale):* 80 mg (99 %); ^{1}H NMR (d_6-DMSO, 300 MHz) δ/ppm = 6.49 (m, 2 H, arom.), 6.36 (m, 2 H, arom.), 4.35 (s, 4 H, NH_2); ^{13}C NMR (d_6-DMSO, 75 MHz) δ/ppm = 134.9, 117.2, 114.5; FTIR-ATR ν/cm^{-1} = 3385, 3364, 3186, 3028, 1630, 1590, 1499, 1458, 1272, 1247, 1155, 1030, 927, 743.

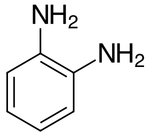

***m*-phenylenediamine (*m*-2b):**

From 3-nitroaniline: 106 mg (98%), *from 1,3-dinitrobenzene (0.75 mmol scale):* 78 mg (96%); ^{1}H NMR (d_6-DMSO, 300 MHz) δ/ppm = 6.65 (t, J = 7.8 Hz, 1 H, arom.), 5.79 (m, 3 H, arom.), 4.60 (s, 4 H, NH_2); ^{13}C NMR (d_6-DMSO, 75 MHz) δ/ppm = 149.0, 129.0, 103.0, 100.0; FTIR-ATR ν/cm^{-1} = 3326, 3211, 1587, 1493, 1316, 1195, 1159, 993, 953, 838, 769, 684.

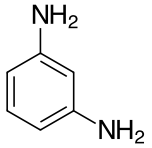

***p*-phenylenediamine (*p*-2b):**

From 4-nitroaniline: 103 mg (95%), *from 1,4-dinitrobenzene (0.75 mmol scale):* 78 mg (96%); ^{1}H NMR ($CDCl_3$, 300 MHz) δ/ppm = 6.57 (s, 4 H, arom.), 3.33 (s, 4 H, NH_2); ^{13}C NMR ($CDCl_3$, 75 MHz) δ/ppm = 138.8, 116.8; FTIR-ATR ν/cm^{-1} = 3373, 3303, 3197, 3009, 1628, 1513, 1310, 1259, 1127, 822, 700, 511.

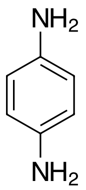

Note: For the mechanochemical reduction of dinitrobenzenes, 1.1 eq of ammonium formate and 2 mol% of Pd/C *per one NO_2 group* was used, giving a total of 4.95 mmol (312 mg) of $HCOONH_4$ and 4 mol% (32 mg) of the catalyst (10 % Pd on activated carbon) on 0.75 mmol scale.

***o*-aminophenol (*o*-2c):** 107 mg (99%); ^{1}H NMR (d_6-DMSO, 300 MHz) δ/ppm = 8.88 (brs, 1 H, OH), 6.67–6.32 (m, 4 H, arom.), 4.45 (brs, 2 H, NH_2); ^{13}C NMR (d_6-DMSO, 75 MHz) δ/ppm = 144.0, 136.5, 119.4, 116.4, 114.4, 114.3; FTIR-ATR ν/cm^{-1} = 3374, 3302, 3051, 2711, 2653, 2584, 1600, 1509, 1459, 1400, 1265, 1216, 1140, 1084, 1030, 892, 845, 739.

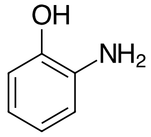

***m*-aminophenol (*m*-2c):** 103 mg (94%); ^{1}H NMR (d_6-DMSO, 300 MHz) δ/ppm = 8.82 (brs, 1 H, OH), 6.76 (t, J = 7.7 Hz, 1 H, arom.), 6.01–5.93 (m, 3 H, arom.), 4.89 (brs, 2 H, NH_2); ^{13}C NMR (d_6-DMSO, 75 MHz) δ/ppm = 158.1, 149.7, 129.3, 105.3, 103.3, 101.0; FTIR-ATR ν/cm^{-1} = 3359, 3294, 3027, 2837, 2710, 2604, 1590, 1506, 1464, 1386, 1303, 1255, 1176, 1150, 1072, 899, 840, 773, 687.

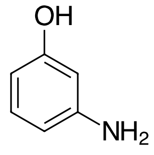

***p*-aminophenol (*p*-2c):** 107 mg (99%); ^{1}H NMR (d_6-DMSO, 300 MHz) δ/ppm = 8.33 (brs, 1 H, OH), 6.47 (d, J = 8.8 Hz, 2 H, arom.), 6.40 (d, J = 8.8 Hz, 2 H, arom.), 4.36 (brs, 2 H, NH_2); ^{13}C NMR (d_6-DMSO, 75 MHz) δ/ppm = 148.2, 140.6, 115.5, 115.2; FTIR-ATR ν/cm^{-1} = 3339, 3280, 3031, 2917, 2810, 2680, 2581, 1613, 1508, 1471, 1384, 1255, 1235, 1168, 1091, 1010, 967, 815, 748, 705, 521, 511.

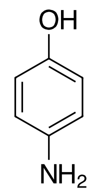

Scale-up procedure: A mixture of 4-nitrophenol (***p*-1c**) (1.391 g, 10.0 mmol), ammonium formate (2.080 g, 33.0 mmol, 1.1 eq), Pd/C (2 mol%, 210.0 mg) and silica (2.000 g) was ball milled in the presence of dry methanol (η = 0.25 μL mg^{-1}) in a 25 mL stainless steel jar with two 12 mm steel balls for 5 hours. The crude mixture was left in a well ventilated hood overnight, suspended in methanol (50 mL) and filtered over a Büchner funnel. Evaporation of the filtrate afforded ***p*-2c** in quantitative yield (1.09 g). FTIR-ATR analysis confirmed that the product was identical to 1.0 mmol scale reaction.

***o*-aminobenzoic acid (*o*-2d):** 134 mg (98%); ^{1}H NMR (d_6-DMSO, 300 MHz) δ/ppm = 9.5–8.0 (brs, 3 H, COOH and NH_2), 7.69 (dd, J = 7.9 Hz, J = 1.6 Hz, 1 H, arom.), 7.20 (t, J = 7.6 Hz, 1 H, arom.), 6.74 (dd, J = 8.2 Hz, J = 0.9 Hz, 1 H, arom.), 6.50 (t, J = 7.5 Hz, 1 H, arom.); ^{13}C NMR (d_6-DMSO, 75 MHz) δ/ppm = 169.6, 151.5, 133.7, 131.2, 116.4, 114.6, 109.6; FTIR-ATR ν/cm^{-1} = 3321, 3235, 1657, 1571, 1368, 1315, 1229, 1136, 928, 834, 751, 690, 658, 540, 504.

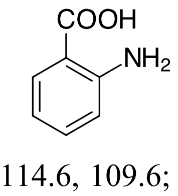

***m*-aminobenzoic acid (*m*-2d):** 135 mg (99%); ^{1}H NMR (d_6-DMSO, 300 MHz) δ/ppm = 11.5–13.5 (brs, 1 H, COOH), 7.03–7.25 (m, 3 H, arom.), 6.76 (td, J = 6.9 Hz, J = 2.3 Hz, 1 H, arom.), 4.60–6.30 (brs, 2 H, NH_2); ^{13}C NMR (d_6-DMSO, 75 MHz) δ/ppm = 167.9, 148.8, 131.3, 128.9, 118.0, 116.6, 114.4; FTIR-ATR ν/cm^{-1} = 2949, 1625, 1557, 1449, 1379, 787, 757, 675, 524.

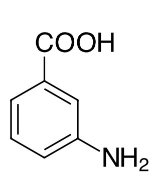

***p*-aminobenzoic acid (*p*-2d):** 133 mg (97 %); ^{1}H NMR (d_6-DMSO, 300 MHz) δ/ppm = 7.60 (d, 2 H, J = 8.7 Hz, arom.), 6.51 (d, 2 H, J = 8.7 Hz, arom.), 5.72 (brs, 3 H, COOH and NH_2); ^{13}C NMR (d_6-DMSO, 75 MHz) δ/ppm = 168.1, 152.4, 131.0, 119.0, 112.5; FTIR-ATR ν/cm^{-1} = 3459, 3361, 2822, 2545, 1660, 1622, 1598, 1420, 1284, 1128, 841, 770, 698, 616, 550, 498.

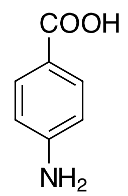

3,5-diaminobenzoic acid (2e): 113 mg (99 %, *0.75 mmol scale*); ^{1}H NMR (d_6-DMSO, 300 MHz) δ/ppm = 6.41 (d, J = 2.1 Hz, 2 H, arom.), 6.01 (t, J = 2.1 Hz, 1 H, arom.), 5.27 (brs, 3 H, COOH and NH_2); ^{13}C NMR (d_6-DMSO, 75 MHz) δ/ppm = 168.4, 149.2, 131.7, 104.1, 103.5; FTIR-ATR ν/cm^{-1} = 3431, 3349, 2976, 1629, 1558, 1520, 1384, 1329, 1148, 1000, 937, 866, 775, 759, 581, 502.

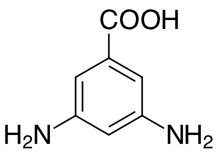

Note: For the mechanochemical reduction of 3,5-dinitrobenzoic acid (**1e**) on 0.75 mmol scale (159 mg), 1.1 eq of ammonium formate and 2 mol% of Pd/C *per one NO_2 group* was used, giving a total of 4.95 mmol (312 mg) of $HCOONH_4$ and 4 mol% (32 mg) of the catalyst (10 % Pd on activated carbon).

2-aminoterephthalic acid (2f): 179 mg (99%); ^{1}H NMR (d_6-DMSO, 300 MHz) δ/ppm = 7.71 (d, J = 8.2 Hz, 1 H, arom.), 7.27 (d, J = 1.4 Hz, 1 H, arom.), 6.96 (dd, J = 8.2 Hz, J = 1.5 Hz, 1 H, arom.); ^{13}C NMR (d_6-DMSO, 75 MHz) δ/ppm = 170.8, 168.6, 150.3, 137.2, 131.0, 117.6, 116.9, 114.9; FTIR-ATR ν/cm^{-1} = 3507, 3392, 2821, 2645, 1671, 1588, 1553, 1495, 1418, 1291, 1226, 879, 752, 587, 491.

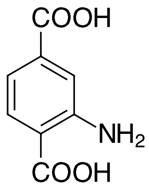

2,5-bis(acetamido)-1-aminobenzene (2g): 194 mg (94%); ^{1}H NMR (d_6-DMSO, 600 MHz) δ/ppm = 9.63 (s, 1 H, NH), 9.00 (s, 1 H, NH), 7.06 (d, J = 1.8 Hz, 1 H, arom.), 7.00 (d, J = 8.5 Hz, 1 H, arom.), 6.67 (dd, J = 8.5 Hz, J = 1.8 Hz, 1 H, arom.), 4.86 (brs, 2 H, NH_2), 2.00 (s, 3 H, CH_3), 1.99 (s, 3 H, CH_3); ^{13}C NMR (d_6-DMSO, 150 MHz) δ/ppm = 168.2, 167.8, 142.3, 137.2, 125.6, 118.9, 107.4, 106.3, 23.9, 23.2; FTIR-ATR ν/cm^{-1} = 3402, 3323, 3211, 3016, 1647, 1619, 1509, 1430, 1364, 1264, 984, 873, 684, 588, 559; HRMS-MALDI found: 230.0905; calc. for $C_{10}H_{13}N_3O_2Na$ $[M+Na]^+$: 230.0905.

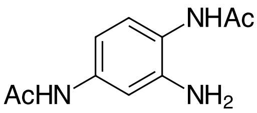

Ethyl *N*-(2,4-diaminophenyl)carbamate (2h): 136 mg (93%, *0.75 mmol scale*); ^{1}H NMR (d_6-DMSO, 300 MHz) δ/ppm = 8.07 (brs, 1 H, NH), 6.65 (d, J = 8.1 Hz, 1 H, arom.), 5.92 (d, J = 2.3 Hz, 1 H, arom.), 5.79 (dd, J = 8.3 Hz, J = 2.4 Hz, 1 H, arom.), 4.56 (brs, 4 H, 2 x NH_2), 4.02 (q, J = 7.1 Hz, 2 H, CH_2), 1.19 (t, J = 7.0 Hz, 3 H, CH_3); ^{13}C NMR (d_6-DMSO, 75 MHz) δ/ppm = 155.1, 147.0, 143.4, 127.0, 112.8, 103.2, 100.7, 59.6, 14.5; FTIR-ATR ν/cm^{-1} = 3343, 2980, 1693, 1620, 1518, 1455, 1324, 1225, 1094, 1055, 967, 842, 773; HRMS-MALDI found: 218.0915; calc. for $C_9H_{13}N_3O_2Na$ $[M+Na]^+$: 218.0905.

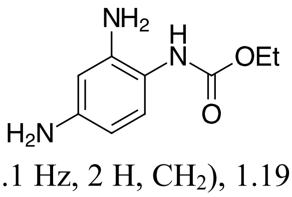

Note: For the mechanochemical reduction of ethyl *N*-(2,4-dinitrophenyl)carbamate (**1h**) on 0.75 mmol scale (191 mg), 1.1 eq of ammonium formate and 2 mol% of Pd/C *per one NO_2 group* was used, giving a total of 4.95 mmol (312 mg) of $HCOONH_4$ and 4 mol% (32 mg) of the catalyst (10 % Pd on activated carbon).

Ethyl *N*-(4-aminophenyl)carbamate (2i): 178 mg (99%); ^{1}H NMR ($CDCl_3$, 300 MHz) δ/ppm = 7.13 (d, J = 8.1 Hz, 2 H, arom.), 6.62 (d, J = 8.5 Hz, 2 H, arom.), 6.45 (s, 1 H, NH), 4.19 (q, J = 7.1 Hz, 2 H, CH_2), 3.36 (brs, 2 H, NH_2), 1.28 (t, J = 7.1 Hz, 3 H, CH_3); ^{13}C NMR ($CDCl_3$, 75 MHz) δ/ppm = 154.3, 142.7, 129.3, 121.1, 115.6, 61.0, 14.6; FTIR-ATR ν/cm^{-1} = 3313, 2980, 1697, 1601, 1515, 1430, 1308, 1224, 1174, 1063, 827, 769, 515; HRMS-MALDI found: 204.0870; calc. for $C_9H_{12}N_2O_2Na$ $[M+Na]^+$: 204.0875.

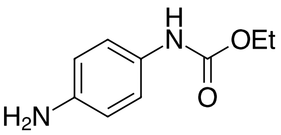

***tert*-Butyl *N*-(3,4-diaminophenyl)carbamate (2j):** 222 mg (99%); ^{1}H NMR (d_6-DMSO, 600 MHz) δ/ppm = 8.60 (s, 1 H, NH), 6.70 (s, 1 H, arom.), 6.30–6.45 (m, 2 H, overlapped arom.), 3.90–4.60 (brs, 4 H, NH_2), 1.44 (s, 9 H, CH_3); ^{13}C NMR (d_6-DMSO, 150 MHz) δ/ppm = 152.9, 135.2, 130.1, 114.5, 108.3, 106.4, 77.9, 28.2; FTIR-ATR ν/cm^{-1} = 3409, 3376, 3335, 2978, 1724, 1699, 1609, 1519, 1427, 1391, 1367, 1296, 1238, 1154, 1055, 955, 849, 718; HRMS-MALDI found: 246.1220; calc. for $C_{11}H_{17}N_3O_2Na$ $[M+Na]^+$: 246.1218.

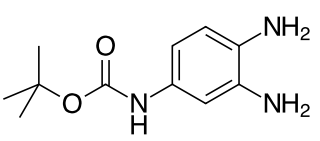

***N*-(*N'*-(*tert*-butoxycarbonyl)-L-alanyl)-3-aminoaniline (2k):** 278 mg (99%); ^{1}H NMR ($CDCl_3$, 600 MHz) δ/ppm = 8.48 (s, 1 H, NH), 7.07 (s, 1 H, arom.), 7.03 (t, J = 7.9 Hz, 1 H, arom.), 6.72 (d, J = 7.8 Hz, 1 H, arom.), 6.39 (d, J = 7.7 Hz, 1 H, arom.), 5.28 (d, J = 7.1 Hz, 1 H, CH), 4.33 (s, 1 H, NH), 3.15–3.50 (brs, 2 H, NH_2), 1.45 (s, 9 H, CH_3), 1.41 (d, J = 7.1 Hz, 3 H, CH_3); ^{13}C NMR ($CDCl_3$, 150 MHz) δ/ppm = 171.0, 156.0, 147.2, 138.8, 129.6, 111.1, 110.0, 106.7, 80.5, 50.9, 28.3, 17.8; FTIR-ATR ν/cm^{-1} = 3316, 2978, 2935, 1668, 1612, 1495, 1455, 1366, 1247, 1161, 1068, 1021, 855, 774, 688; HRMS-MALDI found: 302.1486; calc. for $C_{14}H_{21}N_3O_3Na$ $[M+Na]^+$: 302.1481.

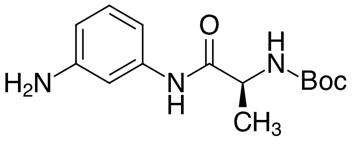

***N*-(*N'*-(Fluorenylmethyloxycarbonyl)-L-phenylalanyl)-3-aminoaniline (2l-Phe):** A mixture of *N*-(*N'*-(fluorenylmethyloxycarbonyl)-L-phenylalanyl)-3-nitroaniline (380.6 mg, 0.75 mmol), ammonium formate (312 mg, 4.95 mmol, 2.2 eq), Pd/C (5 mol%, 40.0 mg) and silica (131 mg) was ball milled in the presence of dry methanol (η = 0.25 μL mg^{-1}) for 3 hours. The crude mixture was left in a well ventilated hood overnight, suspended in methanol : acetone (1 : 1) mixture and filtered over a Büchner funnel. Evaporation of the filtrate afforded the desired amino-derivative **2l-Phe**. 332 mg (93%); ^{1}H NMR (d_6-DMSO, 300 MHz) δ/ppm = 9.80 (s, 1 H, NH), 7.88 (d, J = 7.6 Hz, 2 H, arom.), 7.75 (d, J = 8.3 Hz, 1 H, NH), 7.66 (t, J = 6.2 Hz, 2 H, arom.), 7.17–7.45 (overlapped 9 H, arom.), 6.85–7.03 (overlapped 2 H, arom.), 6.71 (d, J = 7.6 Hz, 1 H, arom.), 6.27 (d, J = 7.6 Hz, 1 H, arom.), 5.08 (s, 2 H, NH_2), 4.41 (m, 1 H, CH), 4.08–4.25 (overlapped 3 H, CH and CH_2), 3.01 (m, 1 H,

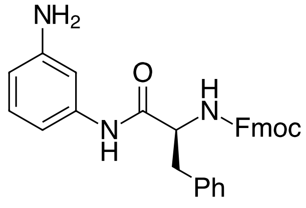

CH_2Ph), 2.87 (m, 1 H, CH_2Ph); ^{13}C NMR (d_6-DMSO, 75 MHz) δ/ppm = 170.2, 155.9, 149.0, 143.8;143.7, 140.7, 139.4, 138.0, 129.2, 128.9, 128.1, 127.6, 127.0, 126.3, 125.3, 120.1, 109.5, 107.3, 105.0, 65.7, 56.9, 46.6, 37.5; FTIR-ATR ν/cm^{-1} = 3469, 3379, 3289, 3063, 1685, 1654, 1619, 1535, 1495, 1445, 1366, 1259, 1220, 1136, 1103, 1086, 1034, 754, 739, 686, 506; HRMS-MALDI found: 500.1927; calc. for $C_{30}H_{27}N_3O_3Na$ [M+Na]$^+$: 500.1950.

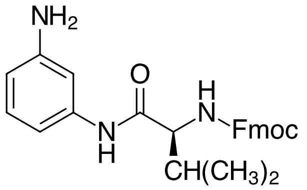

***N*-(*N'*-(Fluorenylmethyloxycarbonyl)-L-valyl)-3-aminoaniline (2l-Val):** A mixture of *N*-(*N'*-(fluorenylmethyloxycarbonyl)-L-valyl)-3-nitroaniline (119.5 mg, 0.26 mmol), ammonium formate (109 mg, 1.72 mmol, 2.2 eq), Pd/C (5 mol%, 13.7 mg) and silica (150 mg) was ball milled in the presence of dry methanol (η = 0.25 µL mg^{-1}) for 3 hours. The crude mixture was left in a well ventilated hood overnight, suspended in methanol : acetone (1 : 1) mixture and filtered over a Büchner funnel. Evaporation of the filtrate afforded the desired amino-derivative **2l-Val**. 101 mg (90%); ^1H NMR (d_6-DMSO, 300 MHz) δ/ppm = 9.72 (s, 1 H, NH), 7.89 (d, J = 7.5 Hz, 2 H, arom.), 7.76 (t, J = 6.8 Hz, 2 H, arom.), 7.57 (d, J = 8.7 Hz, 1 H, NH), 7.41 (t, J = 7.5 Hz, 2 H, arom.), 7.31 (t, J = 7.7 Hz, 2 H, arom.), 6.85–6.98 (overlapped 2 H, arom.), 6.71 (d, J = 7.8 Hz, 1 H, arom.), 6.26 (d, J = 7.7 Hz, 1 H, arom.), 4.71–5.37 (brs, 2 H, NH_2), 4.13–4.33 (m, overlapped 3 H, CH and CH_2), 3.97 (t, J = 8.1 Hz, 1 H, CH), 2.00 (m, 1 H, CH), 0.79–1.05 (overlapped 6 H, CH_3); ^{13}C NMR (d_6-DMSO, 75 MHz) δ/ppm = 170.1, 156.2, 149.0, 143.9;143.8, 140.7, 139.4, 128.9, 127.6, 127.0, 125.4, 120.1, 109.4, 107.3, 105.0, 65.7, 61.1, 46.7, 30.4, 19.2, 18.6; FTIR-ATR ν/cm^{-1} = 3463, 3371, 3292, 2958, 1684, 1653, 1618, 1533, 1496, 1444, 1370, 1287, 1250, 1214, 1082, 1031, 978, 860, 756, 743, 732, 689, 536; HRMS-MALDI found: 452.1928; calc. for $C_{26}H_{27}N_3O_3Na$ [M+Na]$^+$: 452.1950.

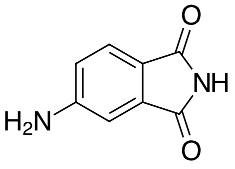

4-aminophthalimide (2m): 157 mg (97%); ^1H NMR (d_6-DMSO, 300 MHz) δ/ppm = 10.68 (s, 1 H, NH), 7.43 (d, J = 8.1 Hz, 1 H, arom.), 6.86 (d, J = 1.9 Hz, 1 H, arom.), 6.79 (dd, J = 8.2 Hz, J = 2.1 Hz, 1 H, arom.), 6.37 (s, 2 H, NH_2); ^{13}C NMR (d_6-DMSO, 75 MHz) δ/ppm = 169.6, 169.3, 154.8, 135.4, 124.6, 117.9, 116.8, 106.6; FTIR-ATR ν/cm^{-1} = 3341, 3350, 3229, 1760, 1709, 1641, 1613, 1586, 1504, 1386, 1322, 1037, 835, 747, 633, 563, 492; HRMS-MALDI found: 163.0508; calc. for $C_8H_7N_2O_2$ [M+H]$^+$: 163.0510.

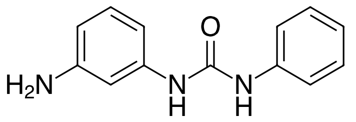

***N*-(3-aminophenyl)-*N'*-phenylurea (2n-O):** 218 mg (96%); ^1H NMR (d_6-DMSO, 300 MHz) δ/ppm = 8.54 (s, 1 H, NH), 8.35 (s, 1 H, NH), 7.43 (d, J = 7.7 Hz, 2 H, arom.), 7.26 (t, J = 7.9 Hz, 2 H, arom.), 6.96 (d, J = 7.3 Hz, 1 H, arom.), 6.89 (t, J = 7.9 Hz, 1 H, arom.), 6.80 (t, J = 1.9 Hz, 1 H, arom.), 6.55 (dd, J = 7.9 Hz, J = 0.9 Hz, 1 H, arom.), 6.18 (dd, J = 7.9 Hz, J = 1.2 Hz, 1 H,

arom.), 5.02 (s, 2 H, NH_2); ^{13}C NMR (d_6-DMSO, 75 MHz) δ/ppm = 152.4, 149.1, 140.2, 139.9, 129.0, 128.8, 121.6, 118.0, 108.1, 106.1, 103.8; FTIR-ATR ν/cm^{-1} = 3302, 1651, 1610, 1592, 1547, 1492, 1441, 1313, 1223, 695; HRMS-MALDI found: 228.1130; calc. for $C_{13}H_{13}N_3O$ $[M]^+$: 228.1137.

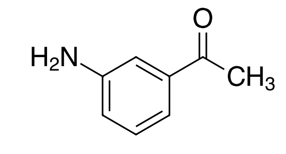

3-aminoacetophenon (2o): A mixture of 3-nitroacetophenone (82.6 mg, 0.5 mmol), ammonium formate (208 mg, 3.3 mmol, 2.2 eq), Pd/C (5 mol%, 26.3 mg) and silica (175 mg) was ball milled in the presence of dry methanol (η = 0.25 μL mg^{-1}) for 6 hours. The crude mixture was left in a well ventilated hood overnight, suspended in methanol and filtered over a Büchner funnel. Evaporation of the filtrate afforded the desired amino-derivative **2o**. 64.4 mg (95%); ^{1}H NMR (d_6-DMSO, 300 MHz) δ/ppm = 7.05–7.20 (m, 3 H, overlapped arom.), 6.79 (ddd, J = 7.3 Hz, J = 4.0 Hz, J = 1.5 Hz, 1 H, arom.), 5.32 (s, 2 H, NH_2), 2.48 (s, 3 H, CH_3); ^{13}C NMR (d_6-DMSO, 75 MHz) δ/ppm = 198.2, 149.0, 137.6, 129.1, 118.5, 115.9, 112.7, 26.7; FTIR-ATR ν/cm^{-1} = 3466, 3368, 2999, 1666, 1626, 1597, 1490, 1457, 1354, 1318, 1286, 1236, 976, 869, 775, 683, 587, 513; HRMS-MALDI found: 136.0760; calc. for $C_8H_{10}NO$ $[M+H]^+$: 136.0762.

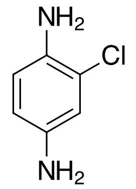

2-chloro-*p*-phenylenediamine (*p*-2p): Purified by column chromatography using DCM:ethyl acetate (1 : 1) mixture as an eluent. 24 mg (17%); ^{1}H NMR (d_6-DMSO, 300 MHz) δ/ppm = 6.56 (d, J = 8.4 Hz, 1 H, arom.), 6.50 (d, J = 2.4 Hz, 1 H, arom.), 6.36 (dd, J = 8.4 Hz, J = 2.5 Hz, 1 H, arom.), 4.43 (brs, 4 H, NH_2); ^{13}C NMR (d_6-DMSO, 75 MHz) δ/ppm = 140.3, 134.7, 118.2, 117.0, 114.7, 114.5; FTIR-ATR ν/cm^{-1} = 3412, 3323, 3032, 1722, 1602, 1500, 1435, 1303, 1234, 1152, 1038, 862, 815, 570; HRMS-MALDI found: 142.0290; calc. for $C_6H_7ClN_2$ $[M]^+$: 142.0298.

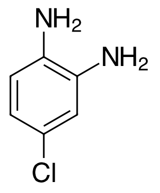

4-chloro-*o*-phenylenediamine (*o*-2p): Purified by column chromatography using DCM:ethyl acetate (1 : 1) mixture as an eluent. 60 mg (42%); ^{1}H NMR (d_6-DMSO, 300 MHz) δ/ppm = 6.50 (d, J = 2.4 Hz, 1 H, arom.), 6.45 (d, J = 8.2 Hz, 1 H, arom.), 6.34 (dd, J = 8.2 Hz, J = 2.4 Hz, 1 H, arom.), 4.70 (brs, 2 H, NH_2), 4.57 (brs, 2 H, NH_2); ^{13}C NMR (d_6-DMSO, 75 MHz) δ/ppm = 136.6, 133.8, 120.3, 116.1, 114.9, 113.3; FTIR-ATR ν/cm^{-1} = 3400, 3310, 1624, 1587, 1496, 1424, 1274, 1238, 1089, 907, 855, 804, 648; HRMS-MALDI found: 142.0291; calc. for $C_6H_7ClN_2$ $[M]^+$: 142.0298.

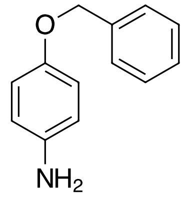

***O*-benzyl-*p*-aminophenol (2q):** Purified by column chromatography using DCM (to remove nitro-reactant **1q**) and DCM:MeOH (20 : 1) as an eluent. 73 mg (37%); ^{1}H NMR (d_6-DMSO, 300 MHz) δ/ppm = 7.25–7.47 (m, 5 H, overlapped phenyl), 6.71 (d, J = 8.8 Hz, 2 H, aminophenol), 6.50 (d, J = 8.8 Hz, 2 H, aminophenol), 4.94 (s, 2 H, CH_2), 4.55–4.73 (brs, 2 H, NH_2); ^{13}C NMR (d_6-DMSO, 75 MHz) δ/ppm = 151.2,

149.7, 142.6, 137.8, 128.3, 127.5, 115.7, 114.9, 69.8; FTIR-ATR ν/cm^{-1} = 3376, 3310, 3193, 3062, 3035, 2908, 2864, 1610, 1588, 1508, 1468, 1454, 1378, 1295, 1228, 1170, 1007, 862, 819, 734, 695, 514; HRMS-MALDI found: 200.1070; calc. for $C_{13}H_{14}NO$ $[M+H]^+$: 200.1075.

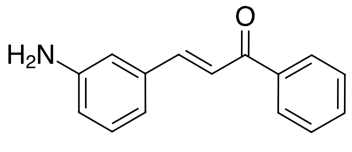

***m*-aminochalcone (*m*-2r):** 221 mg (99%); ^{1}H NMR (d_6-DMSO, 600 MHz) δ/ppm = 8.09 (d, J = 7.3 Hz, 2 H, arom.), 7.69 (t, J = 15.6 Hz, 1 H, alkene), 7.66 (d, J = 14.7 Hz, 1 H, alkene), 7.54–7.62 (m, 3 H, overlapped arom.), 7.11 (t, J = 7.7 Hz, 1 H, arom.), 7.02 (d, J = 7.6 Hz, 1 H, arom.), 6.97 (s, 1 H, arom.), 6.68 (dd, J = 7.9 Hz, J = 1.4 Hz, arom.), 5.19 (s, 2 H, NH_2); ^{13}C NMR (d_6-DMSO, 150 MHz) δ/ppm = 189.3, 149.0, 145.1, 137.7, 135.0, 132.9, 129.3, 128.7, 128.3, 121.1, 116.6, 116.5, 113.6; FTIR-ATR ν/cm^{-1} = 3440, 3343, 1656, 1592, 1573, 1492, 1448, 1341, 1310, 1280, 1213, 1018, 982, 768, 687; HRMS-MALDI found: 246.0892; calc. for $C_{15}H_{13}NONa$ $[M+Na]^+$: 246.0895.

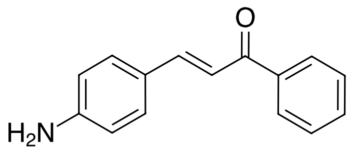

***p*-aminochalcone (*p*-2r):** 222 mg (99%); ^{1}H NMR (d_6-DMSO, 300 MHz) δ/ppm = 8.08 (d, J = 7.2 Hz, 2 H, arom.), 7.46–7.70 (m, 7 H, overlapped arom.), 6.60 (d, J = 8.5 Hz, 2 H, arom.), 5.90 (s, 2 H, NH_2); ^{13}C NMR (d_6-DMSO, 75 MHz) δ/ppm = 188.6, 152.1, 145.6, 138.4, 132.4, 131.1, 128.6, 128.1, 121.9, 115.3, 113.6; FTIR-ATR ν/cm^{-1} = 3432, 3336, 1636, 1593, 1553, 1515, 1440, 1311, 1214, 1173, 993, 829, 781, 688, 517; HRMS-MALDI found: 246.0898; calc. for $C_{15}H_{13}NONa$ $[M+Na]^+$: 246.0895.

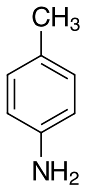

***p*-toluidine (2s):** 106 mg (99%); ^{1}H NMR ($CDCl_3$, 600 MHz) δ/ppm = 6.95 (d, J = 7.9 Hz, 2 H, arom.), 6.59 (d, J = 8.1 Hz, 2 H, arom.), 3.50 (s, 2 H, NH_2), 2.23 (s, 3 H, CH_3); ^{13}C NMR ($CDCl_3$, 150 MHz) δ/ppm = 143.8, 129.7, 127.7, 115.2, 20.4; FTIR-ATR ν/cm^{-1} = 3418, 3337, 3222, 3010, 2912, 2859, 2738, 1879, 1621, 1513, 1280, 1268, 1178, 1122, 810, 679, 503.

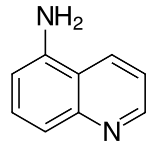

5-aminoquinoline (2t): Purified by column chromatography using DCM:MeOH (20 : 1) as an eluent. 139 mg (96%); ^{1}H NMR (d_6-DMSO, 600 MHz) δ/ppm = 8.76 (dd, J = 4.1 Hz, J = 1.6 Hz, 1 H, arom.), 8.51 (d, J = 8.4 Hz, 1 H, arom.), 7.41 (t, J = 8.0 Hz, 1 H, arom.), 7.34 (dd, J = 8.5 Hz, J = 4.1 Hz, 1 H, arom.), 7.18 (d, J = 8.3 Hz, 1 H, arom.), 6.71 (d, J = 7.6 Hz, 1 H, arom.), 5.94 (s, 2 H, NH_2); ^{13}C NMR (d_6-DMSO, 150 MHz) δ/ppm = 149.7, 148.9, 145.3, 130.8, 130.1, 118.7, 117.5, 116.0, 107.3; FTIR-ATR ν/cm^{-1} = 3328, 3194, 1657, 1613, 1585, 1574, 1507, 1463, 1411, 1365, 1325, 1280, 1206, 1082, 1049, 1011, 825, 789, 699, 566, 472; HRMS-MALDI found: 145.0770; calc. for $C_9H_9N_2$ $[M+H]^+$: 145.0766.

5-amino-6-methylquinoline (2u): Reaction time 3 h, purified by column chromatography using diethylether as an eluent. 133 mg (84%); ^{1}H NMR (d_6-DMSO, 600 MHz) δ/ppm = 8.71 (dd, J = 4.0 Hz, J = 1.2 Hz, 1 H, arom.), 8.58 (d, J = 8.5 Hz, 1 H, arom.), 7.36 (d, J = 8.4 Hz, 1 H, arom.), 7.33 (dd, J = 8.5 Hz, J = 4.1 Hz, 1 H, arom.), 7.17 (d, J = 8.4 Hz, 1 H, arom.), 5.62 (s, 2 H, NH_2), 2.25 (s, 3 H, CH_3); ^{13}C NMR (d_6-DMSO, 150 MHz) δ/ppm = 148.7, 147.6, 141.6, 132.8, 130.4, 118.7, 117.1, 115.9, 114.4, 17.7; FTIR-ATR ν/cm^{-1} = 3442, 3318, 3183, 2966, 2920, 1642, 1570, 1509, 1468, 1405, 1377, 1356, 1090, 823, 798, 567, 476; HRMS-MALDI found: 159.0915; calc. for $C_{10}H_{11}N_2$ [M+H]$^+$: 159.0922.

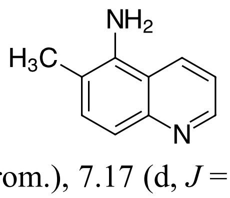

8-aminoquinoline (2v): 129 mg (90%); ^{1}H NMR (d_6-DMSO, 300 MHz) δ/ppm = 8.71 (dd, J = 4.1 Hz, J = 1.7 Hz, 1 H, arom.), 8.16 (dd, J = 8.3 Hz, J = 1.7 Hz, 1 H, arom.), 7.45 (dd, J = 8.3 Hz, J = 4.1 Hz, 1 H, arom.), 7.26 (t, J = 7.8 Hz, 1 H, arom.), 7.05 (dd, J = 8.1 Hz, J = 1.1 Hz, 1 H, arom.), 6.86 (dd, J = 7.5 Hz, J = 1.2 Hz, 1 H, arom.), 5.91 (s, 2 H, NH_2); ^{13}C NMR (d_6-DMSO, 75 MHz) δ/ppm = 146.9, 145.2, 137.4, 135.8, 128.5, 127.6, 121.4, 113.7, 108.6; FTIR-ATR ν/cm^{-1} = 3452, 3350, 3034, 1924, 1614, 1590, 1504, 1470, 1427, 1368, 1335, 1125, 1094, 890, 818, 788, 759, 537; HRMS-MALDI found: 145.0765; calc. for $C_9H_9N_2$ [M+H]$^+$: 145.0766.

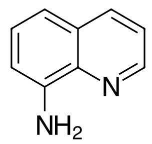

6-aminotetralin (2w): *Standard procedure (1.0 mmol scale, 1.1 eq of $HCOONH_4$, 2 mol% of Pd/C, 90 minutes) gave 59 mg (40%) of **2w** after column chromatography.* A mixture of 6-nitrotetralin (180.3 mg, 1.012 mmol), ammonium formate (421 mg, 6.68 mmol, 2.2 eq), Pd/C (5 mol%, 53.1 mg) and silica (175 mg) was ball milled in the presence of dry methanol (η = 0.2 µL mg^{-1}, 166 µL) for 3 hours. The crude mixture was suspended in methanol and filtered over a Büchner funnel. Following evaporation of the filtrate and purification over a short silicagel column using DCM as an eluent, the desired amino-derivative **2w** was isolated. 146 mg (97%); ^{1}H NMR ($CDCl_3$, 600 MHz) δ/ppm = 6.87 (d, J = 8.0 Hz, 1 H, arom.), 6.48 (dd, J = 8.0 Hz, J = 2.3 Hz, 1 H, arom.), 6.44 (s, 1 H, arom.), 3.25–3.75 (brs, 2 H, NH_2), 2.64–2.72 (m, 4 H, overlapped CH_2), 1.72–1.82 (m, 4 H, overlapped CH_2); ^{13}C NMR ($CDCl_3$, 150 MHz) δ/ppm = 143.9, 137.9, 129.8, 127.3, 115.4, 113.2, 29.5, 28.6, 23.6, 23.3; FTIR-ATR ν/cm^{-1} = 3345, 3002, 2922, 2855, 2836, 1621, 1504, 1443, 1352, 1289, 1272, 1158, 904, 859, 824, 803; HRMS-MALDI found: 170.0944; calc. for $C_{10}H_{13}NNa$ [M+Na]$^+$: 170.0946.

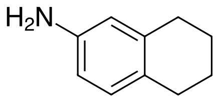

1-aminonaphthalene (2x): *Not isolated, yield determined based on ^{1}H NMR analysis of the reaction mixture after workup.* Standard conditions (1.0 mmol scale, 1.1 eq of $HCOONH_4$, 2 mol% of Pd/C, 90 minutes) afforded a mixture of unreacted 1-nitronaphthalene (**1x**) and 1-aminonaphthalene (**2x**) in 82 : 18 ratio. Prolonging the reaction time to 6 hours led to 63 : 37 mixture of **1x** and **2x**, respectively. See Figure S1 for the ^{1}H NMR spectrum.

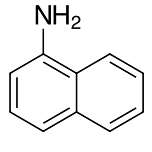

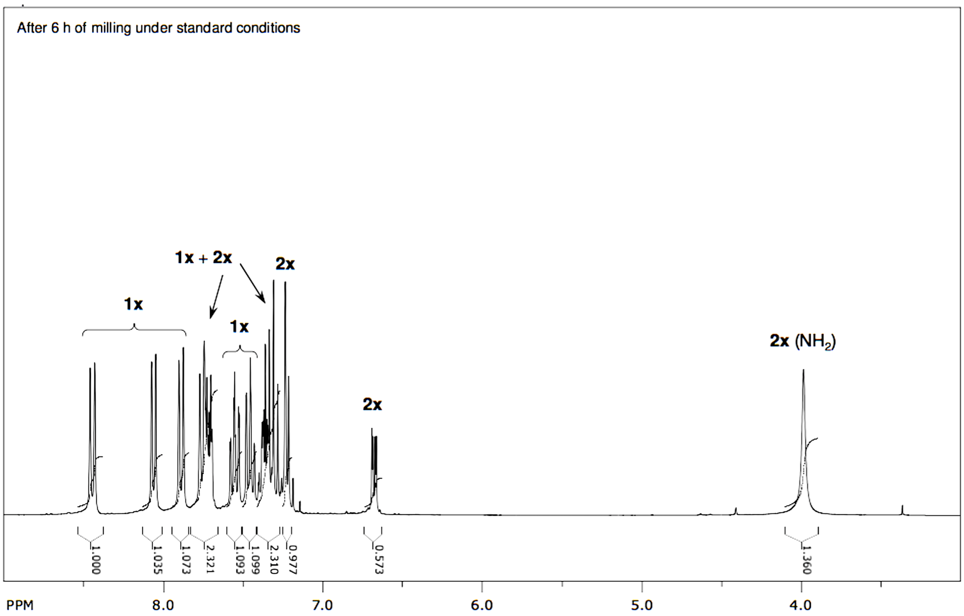

Figure S1. ^{1}H NMR spectrum of the crude reaction mixture after 6 hours of milling under standard conditions (1.0 mmol scale, 1.1 eq $HCOONH_4$, 2 mol% Pd/C) for the catalytic transfer hydrogenation of 1-nitronaphthalene (**1x**) to 1-aminonaphthalene (**2x**). Integration of the corresponding signals revealed the ratio **1x** : **2x** = 63 : 37.

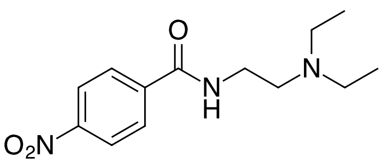

***N*-[2-(diethylamino)ethyl]-4-nitrobenzamide (4):** A mixture of 4-nitrobenzoyl chloride (205.0 mg, 1.1 mmol), *N*,*N*-diethylethylenediamine (116.2 mg, 141 μL, 1.0 mmol), anhydrous potassium carbonate (278.0 mg, 2.0 mmol) and silica (250 mg) was ball milled in teflon media (10 mm ball and 10 mL jar) at 30 Hz for 60 minutes. The reaction was run in parallel, the two crude mixtures were combined and suspended in 100 mL of dist. H_2O : methanol (1 : 1) mixture. The filtrate was concentrated in vacuuo until all methanol was removed and the aqueous solution extracted with ethyl acetate (3 x 25 mL). Combined organic extracts were dried on anhydrous $MgSO_4$ and evaporation of the filtrate afforded nitro-intermediate **4** as a yellow oil that crystallized on standing overnight. 468.0 mg (88%); ^{1}H NMR ($CDCl_3$, 300 MHz) δ/ppm = 8.29 (d, J = 8.8 Hz, 2 H, arom.), 7.94 (d, J = 8.8 Hz, 2 H, arom.), 7.06–7.20 (brs, 1 H, NH), 3.51 (q, J = 5.3 Hz, 2 H, CH_2), 2.68 (t, J = 5.8 Hz, 2 H, CH_2), 2.59 (q, J = 7.1 Hz, 4 H, CH_2), 1.05 (t, J = 7.1 Hz, 6 H, CH_3); ^{13}C NMR ($CDCl_3$, 75 MHz) δ/ppm = 165.1, 149.5, 140.4, 128.0, 123.8, 51.0, 46.7, 37.5, 12.0; FTIR-ATR ν/cm^{-1} = 3330, 3058, 2971, 2936, 2821, 1638, 1600, 1543, 1523, 1492, 1338, 1298, 1178, 1069, 875, 841, 776, 722, 687, 644, 622, 503; HRMS-MALDI found: 266.1511; calc. for $C_{13}H_{20}N_3O_3$ $[M+H]^+$: 266.1505.

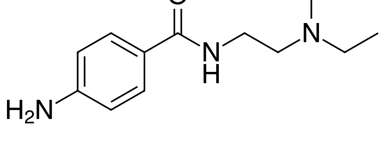

Procainamide (5): 233 mg (99%); ^{1}H NMR ($CDCl_3$, 300 MHz) δ/ppm = 7.61 (d, J = 8.6 Hz, 2 H, arom.), 6.82–7.00 (brs, 1 H, NH), 6.64 (d, J = 8.6 Hz, 2 H, arom.), 3.80–4.20 (brs, 2 H, NH_2), 3.46 (q, J = 5.5

Hz, 2 H, CH_2), 2.64 (t, *J* = 5.5 Hz, 2 H, CH_2), 2.57 (q, *J* = 7.2 Hz, 4 H, CH_2), 1.03 (t, *J* = 7.2 Hz, 6 H, CH_3); ^{13}C NMR ($CDCl_3$, 75 MHz) δ/ppm = 167.2, 149.4, 128.6, 124.3, 114.1, 51.5, 46.8, 37.1, 11.8; FTIR-ATR ν/cm^{-1} = 3336, 3221, 2968, 2816, 1602, 1543, 1504, 1287, 1183, 1067, 841, 768; HRMS-MALDI found: 258.1580; calc. for $C_{13}H_{21}N_3ONa$ $[M+Na]^+$: 258.1582.

Scale-up procedure: A mixture of nitro-intermediate **4** (1.327 g, 5.0 mmol), ammonium formate (1.040 g, 16.5 mmol, 1.1 eq), Pd/C (2 mol%, 105.0 mg) and silica (1.000 g) was ball milled in the presence of dry methanol (η = 0.25 µL mg^{-1}) in a 25 mL stainless steel jar with two 12 mm steel balls for 6 hours. The crude mixture was left in a well ventilated hood overnight, suspended in methanol (50 mL) and filtered over a Büchner funnel. Evaporation of the filtrate afforded procainamide (**5**) as oil in quantitative yield (1.180 g). FTIR-ATR analysis confirmed that the product was identical to 1.0 mmol scale reaction.

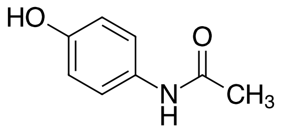

Paracetamol (6): A 10 mL teflon jar was charged with 4-aminophenol (***p*-2c**) (218.3 mg, 2.0 mmol) and silica (250 mg). Acetic anhydride (Ac_2O) was added using a pipette (204.2 mg, 189 µL, 2.0 mmol) and the mixture was milled at 30 Hz for 30 minutes. The crude mixture was left in a fume hood to allow acetic acid to evaporate and was then suspended in 10 mL of methanol. After filtration and evaporation, paracetamol (**6**) was isolated. FTIR-ATR analysis confirmed that **6** was identical to the reference sample of paracetamol provided by Pliva TAPI. 298 mg (99%); ^{1}H NMR (d_6-DMSO, 300 MHz) δ/ppm = 9.63 (s, 1 H, NH), 9.15 (s, 1 H, OH), 7.32 (d, *J* = 8.9 Hz, 2 H, arom.), 6.66 (d, *J* = 8.9 Hz, 2 H, arom.), 1.97 (s, 3 H, CH_3); ^{13}C NMR (d_6-DMSO, 75 MHz) δ/ppm = 167.5, 153.1, 131.0, 120.8, 115.0, 23.7; FTIR-ATR ν/cm^{-1} = 3322, 3160, 1651, 1609, 1561, 1505, 1436, 1370, 1327, 1258, 1225, 1172, 1108, 968, 836, 807, 682, 603, 518, 503.

Scale-up procedure: A mixture of 4-aminophenol (***p*-2c**) (720.4 mg, 6.6 mmol), silica (825 mg) and acetic anhydride (673.9 mg, 624 µL, 6.6 mmol) was ball milled in a 10 mL teflon jar with two 10 mm teflon balls for 2 hours. The crude mixture was left in a well ventilated hood overnight, suspended in methanol (40 mL) and filtered over a Büchner funnel. Evaporation of the filtrate afforded paracetamol (**6**) in 99% yield (991 mg). FTIR-ATR analysis confirmed that the product was identical to 2.0 mmol scale reaction.

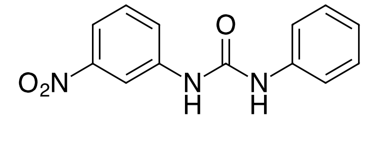

N*-(3-nitrophenyl)-*N′*-phenylurea (1n-O):** A mixture of 3-nitroaniline (m*-1b**) (138.1 mg, 1.0 mmol) and phenyl isocyanate (119.1 mg, 109 µL, 1.0 mmol) was ball milled in the presence of dry acetonitrile (η = 0.25 µL mg^{-1}) in a 10 mL stainless steel jar using a single 10 mm steel ball for 60 minutes. The reaction was run in parallel, the two crude mixtures were combined, suspended in methanol (20 mL) and filtered over a short celite plug. Evaporation of the filtrate

afforded nitrourea **1n-O**. 475 mg (92%); ^{1}H NMR (d_6-DMSO, 300 MHz) δ/ppm = 9.19 (s, 1 H, NH), 8.81 (s, 1 H, NH), 8.56 (t, J = 2.1 Hz, 1 H, arom.), 7.82 (dd, J = 8.1 Hz, J = 0.8 Hz, 1 H, arom.), 7.71 (dd, J = 8.0 Hz, J = 0.8 Hz, 1 H, arom.), 7.56 (t, J = 8.2 Hz, 1 H, arom.), 7.48 (d, J = 7.8 Hz, 2 H, arom.), 7.30 (t, J = 7.7 Hz, 2 H, arom.), 7.00 (t, J = 7.4 Hz, 1 H, arom.); ^{13}C NMR (d_6-DMSO, 75 MHz) δ/ppm = 152.4, 148.1, 141.0, 139.2, 130.0, 128.8, 124.2, 122.3, 118.6, 116.2, 112.1; FTIR-ATR ν/cm^{-1} = 3281, 3089, 1637, 1595, 1556, 1522, 1446, 1349, 1313, 1235, 1090, 1051, 891, 799, 743, 689, 667, 498; HRMS-MALDI found: 258.0872; calc. for $C_{13}H_{12}N_3O_3$ [M+H]$^+$: 258.0879.

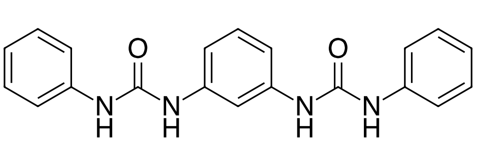

***N*^{1},*N*^{2}-di(*N*-phenylcarbamoyl)-1,3-diaminobenzene (7a):** A mixture of amino-urea **2n-O** (136.4 mg, 0.6 mmol) and phenyl isocyanate (71.5 mg, 65.3 μL, 0.6 mmol) was ball milled in a 10 mL stainless steel jar using a single 12 mm steel ball for 30 minutes. The crude reaction mixture was washed with DCM (2 mL) and dried in air to afford bis-urea **7a**. 203 mg (98%); ^{1}H NMR (d_6-DMSO, 600 MHz) δ/ppm = 8.68 (s, 2 H, NH), 8.58 (s, 2 H, NH), 7.67 (s, 1 H, arom.), 7.45 (d, J = 7.2 Hz, 4 H, arom.), 7.28 (t, J = 7.2 Hz, 4 H, arom.), 7.17 (t, J = 7.8 Hz, 1 H, arom.), 7.07 (d, J = 7.3 Hz, 2 H, arom.), 6.97 (t, J = 6.9 Hz, 2 H, arom.); ^{13}C NMR (d_6-DMSO, 150 MHz) δ/ppm = 152.4, 140.1, 139.6, 129.0, 128.7, 121.7, 118.1, 111.7, 107.8; FTIR-ATR ν/cm^{-1} = 3295, 1638, 1593, 1551, 1491, 1444, 1407, 1296, 1220, 787, 729, 694, 633; HRMS-MALDI found: 347.1498; calc. for $C_{20}H_{19}N_4O_2$ [M+H]$^+$: 347.1508.

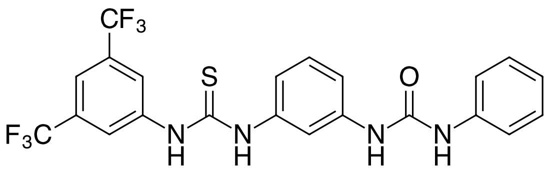

***N*^{1}-[*N*-(3,5-di(trifluoromethyl)phenylthiocarbamoyl]-*N*^{2}-(*N*-phenylcarbamoyl)-1,3-diaminobenzene (7b):** A mixture of amino-urea **2n-O** (102.3 mg, 0.45 mmol) and 3,5-di(trifluoromethyl)phenyl isothiocyanate (122.0 mg, 82.2 μL, 0.45 mmol) was ball milled in the presence of dry acetonitrile (η = 0.25 μL mg^{-1}) in a 10 mL stainless steel jar using a single 12 mm steel ball for 60 minutes. The crude reaction mixture was washed with DCM (2 mL) and dried in air to afford mixed urea-thiourea product **7b**. 208 mg (93%); ^{1}H NMR (d_6-DMSO, 600 MHz) δ/ppm = 10.30 (s, 1 H, NH), 10.18 (s, 1 H, NH), 8.74 (s, 1 H, NH), 8.64 (s, 1 H, NH), 8.27 (s, 2 H, arom.), 7.78 (s, 1 H, arom.), 7.61 (s, 1 H, arom.), 7.45 (d, J = 7.7 Hz, 2 H, arom.), 7.23–7.35 (m, 4 H, arom.), 7.08 (d, J = 6.9 Hz, 1 H, arom.), 6.97 (t, J = 7.1 Hz, 1 H, arom.); ^{13}C NMR (d_6-DMSO, 150 MHz) δ/ppm = 179.5, 152.4, 141.9, 140.1, 139.5, 138.9, 130.3;130.0;129.8;129.6 (q, J = 33.1 Hz), 129.0, 128.7, 125.9;124.1;122.3;120.5 (q, J = 272.5 Hz), 123.4, 121.8, 118.2, 117.3, 116.7, 115.0, 113.5; FTIR-ATR ν/cm^{-1} = 3306, 1683, 1598, 1544, 1474, 1447, 1383, 1313, 1279, 1218, 1175, 1131, 954, 886, 749, 681; HRMS-MALDI found: 499.1037; calc. for $C_{22}H_{17}N_4OSF_6$ [M+H]$^+$: 499.1027.

4. Catalytic transfer hydrogenation of 3-nitrobenzonitrile under aging conditions

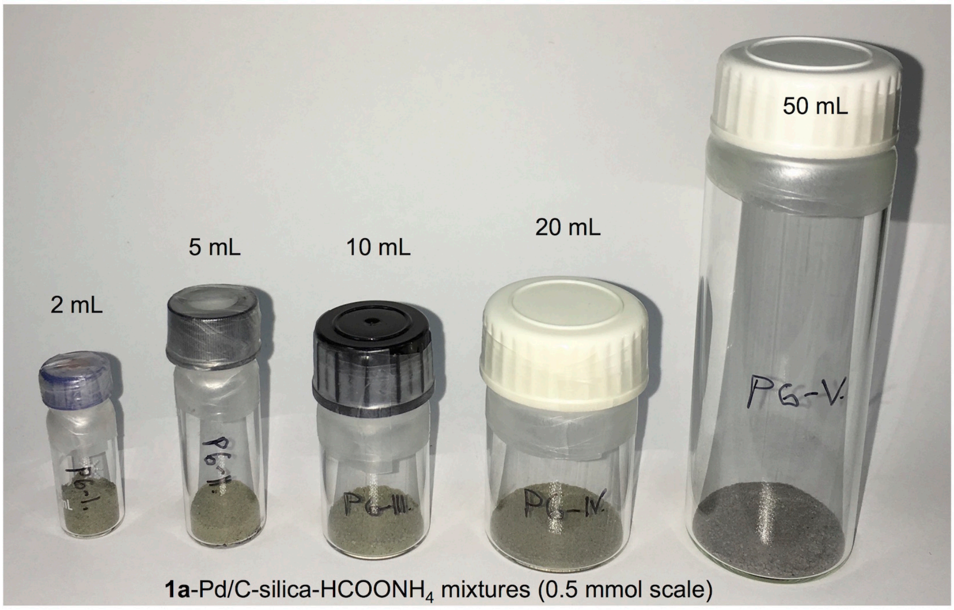

Figure S2. Aging experiments in vials of different volume. Each vial contained a mixture of 0.5 mmol of 3-nitrobenzonitrile (**1a**), 1.65 mmol of $HCOONH_4$ (104 mg, 1.1 eq), 2 mol% of Pd/C catalyst (10.5 mg) and 87.5 mg of silica. This mixture was prepared by gentle manual grinding of **1a**, Pd/C and silica in a mortar, the resulting fine powder was then transferred to a vial, pre-ground ammonium formate was stirred in and quickly sealed. The final mixture was gently shaken in the vial for ca. 10 seconds and left undisturbed for 3 days.

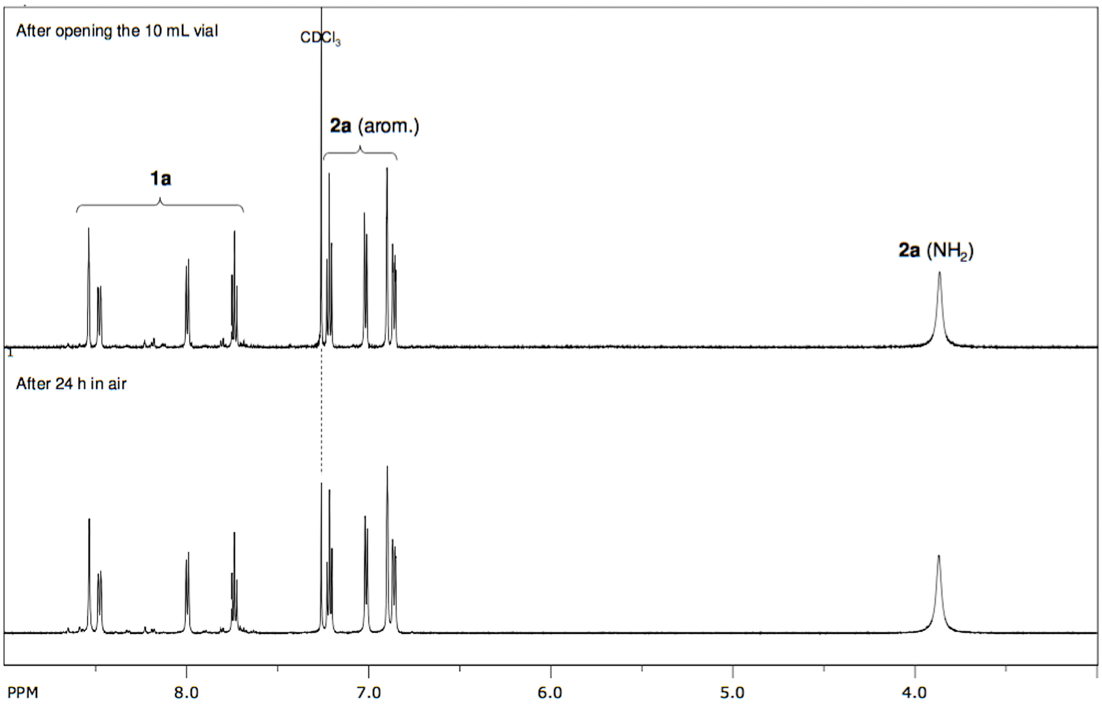

Figure S3. Representative ^{1}H NMR spectra of the mixture taken from the 10 mL vial immediately after opening and 24 h in air. The spectra were recorded in $CDCl_3$. Notably, there is no significant change in the amount of 3-cyanoaniline (**2a**), confirming that upon exposure to air ("open system") the NO_2-reduction stops and the primary reaction path becomes Pd-catalyzed decomposition of $HCOONH_4$.

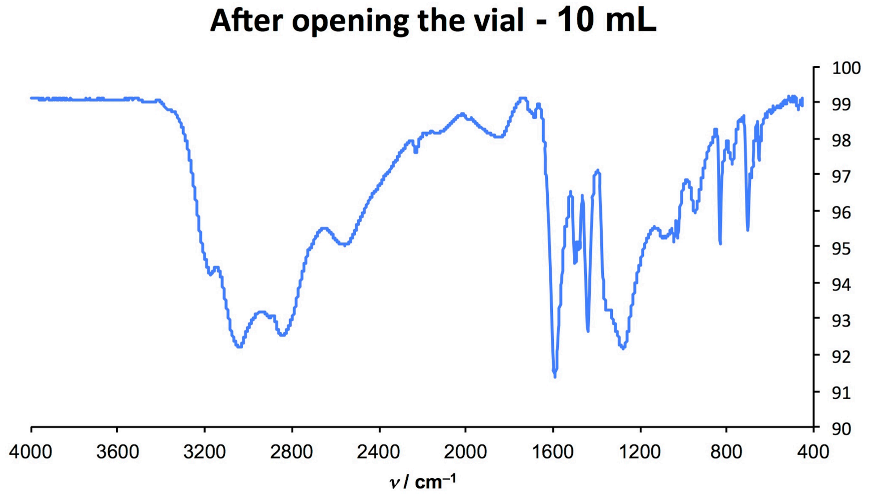

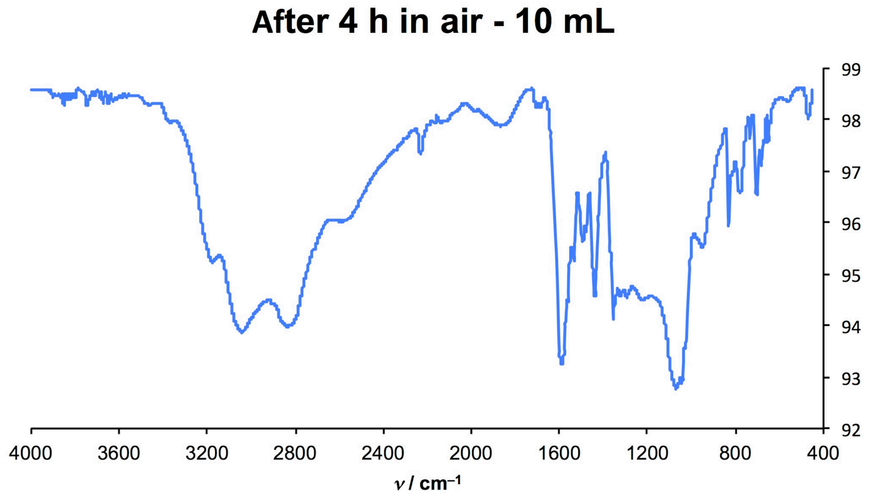

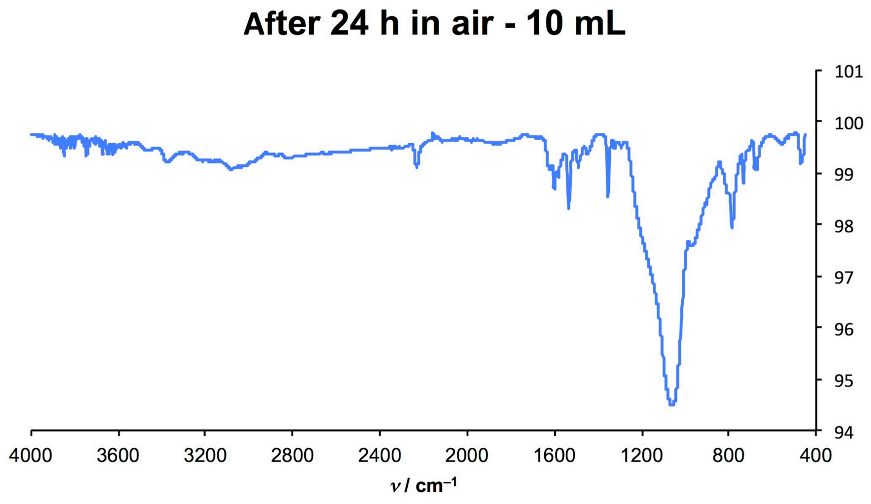

Figure S4. Representative IR spectra of the aging mixture from 10 mL vial immediately after opening, 4 h and 24 h in air. Ammonium formate decomposes in air revealing the absorption bands of **1a**, **2a** and silica after 24 h.

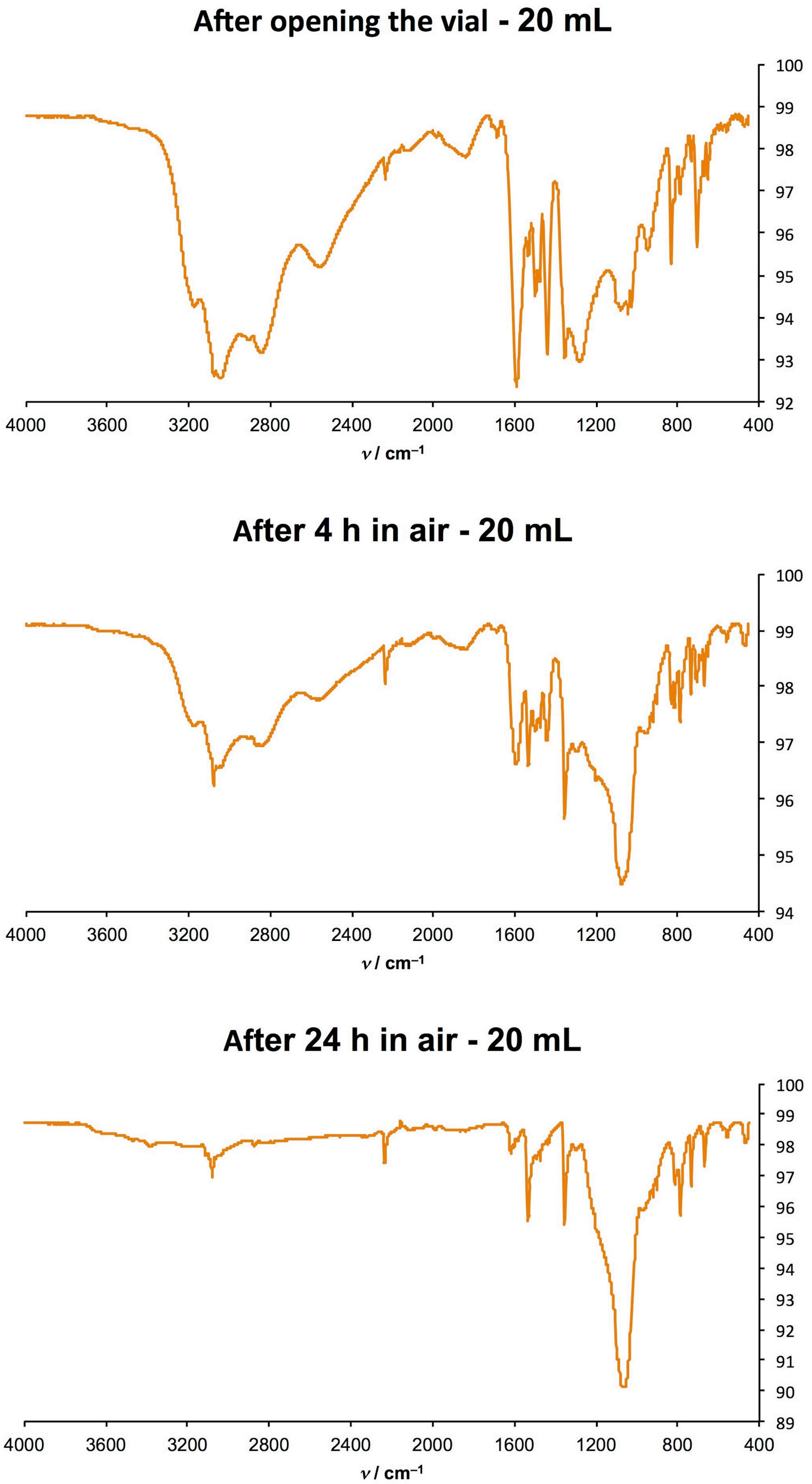

Figure S5. Representative IR spectra of the aging mixture from 20 mL vial immediately after opening, 4 h and 24 h in air. Ammonium formate decomposes in air revealing the absorption bands of **1a** and silica after 24 h.

5. SEM and EDS analysis of the commercial and milled Pd/C catalyst samples

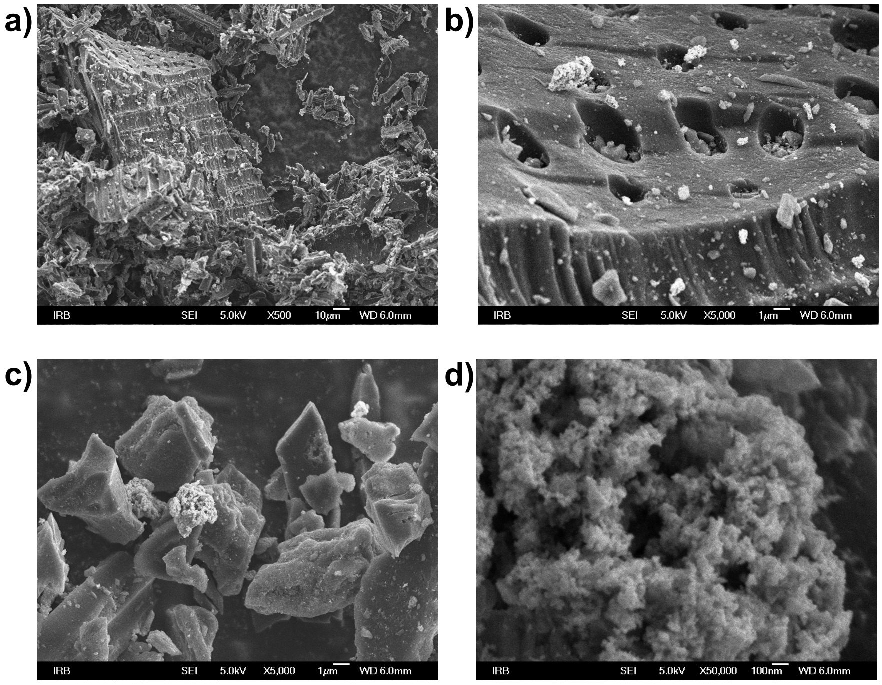

Figure S6. SEM analysis of commercial Pd/C catalyst (10 wt%). a-c) Typical morphology of carbon particles (ca. 10–100 μm) with palladium aggregates (shown in light grey to white) of different sizes (from ca. 100 nm to several μm) distributed on carbon surface (500x and 5000x). d) A close-up view of one Pd aggregate (50000x). Each aggregate is composed of many nanometer-sized Pd particles.

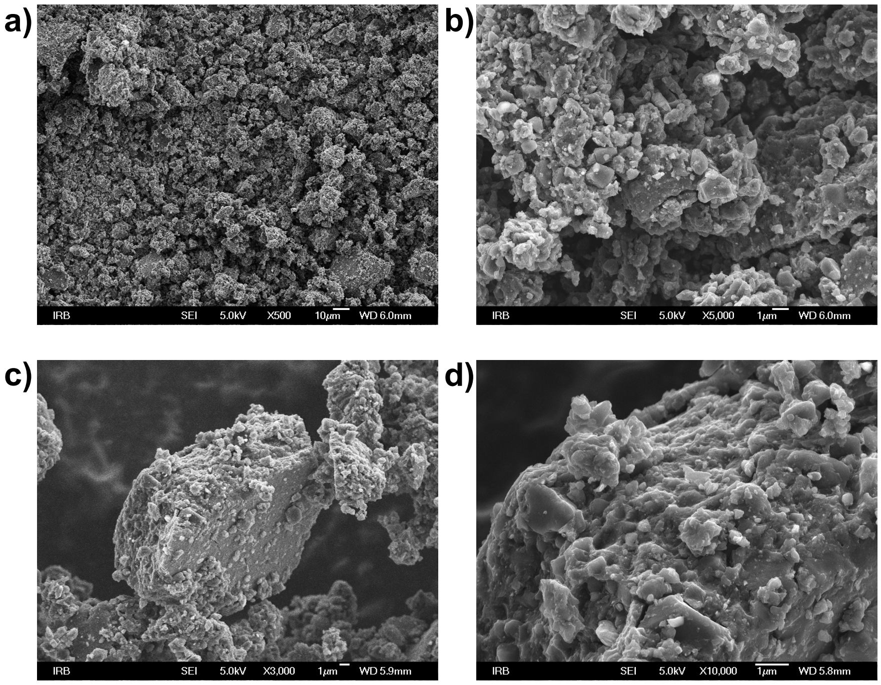

Figure S7. SEM analysis of commercial Pd/C catalyst (10 wt%) milled for 60 mintues at 30 Hz using a single 12 mm (7.0 g) stainless steel ball. Morphology of the samples a-d (500x-10000x) is characteristic for homogenization and pulverization of carbon particles during ball milling resulting in particle sizes ca. 0.5–5 μm.

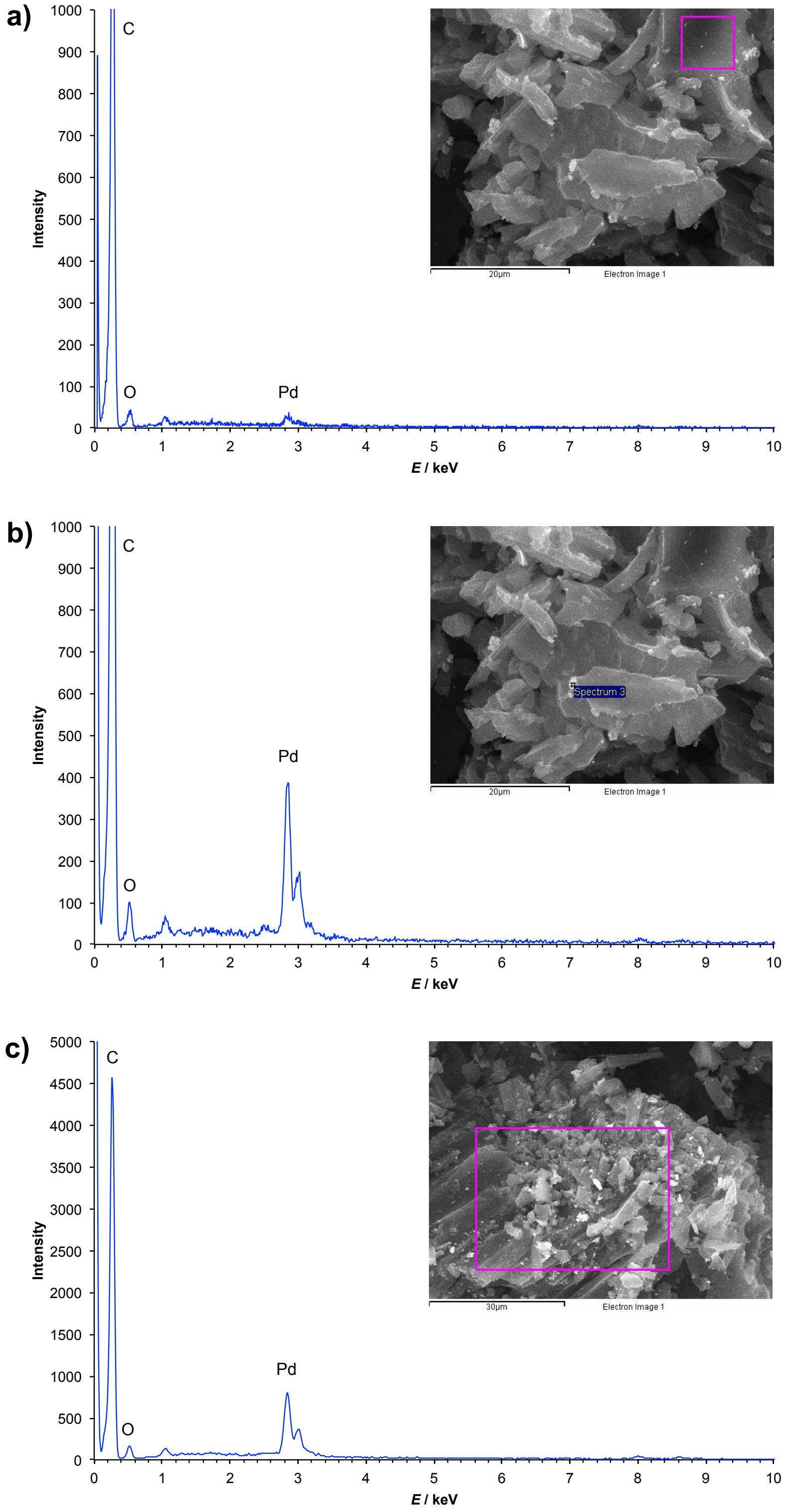

Figure S8. SEM-EDS analysis of commercial palladium on carbon (10 wt%). A distinctive feature of the catalyst as determined by SEM is the organization of palladium into aggregates (seen as small light particles) on the surface of larger carbon particles. a) Analysis of the selected area on a large carbon particle shows traces of Pd, b) point analyisis on the same carbon particle identifies the palladium aggregate, c) analysis of the selected area comprising both carbon particles and Pd aggregates.

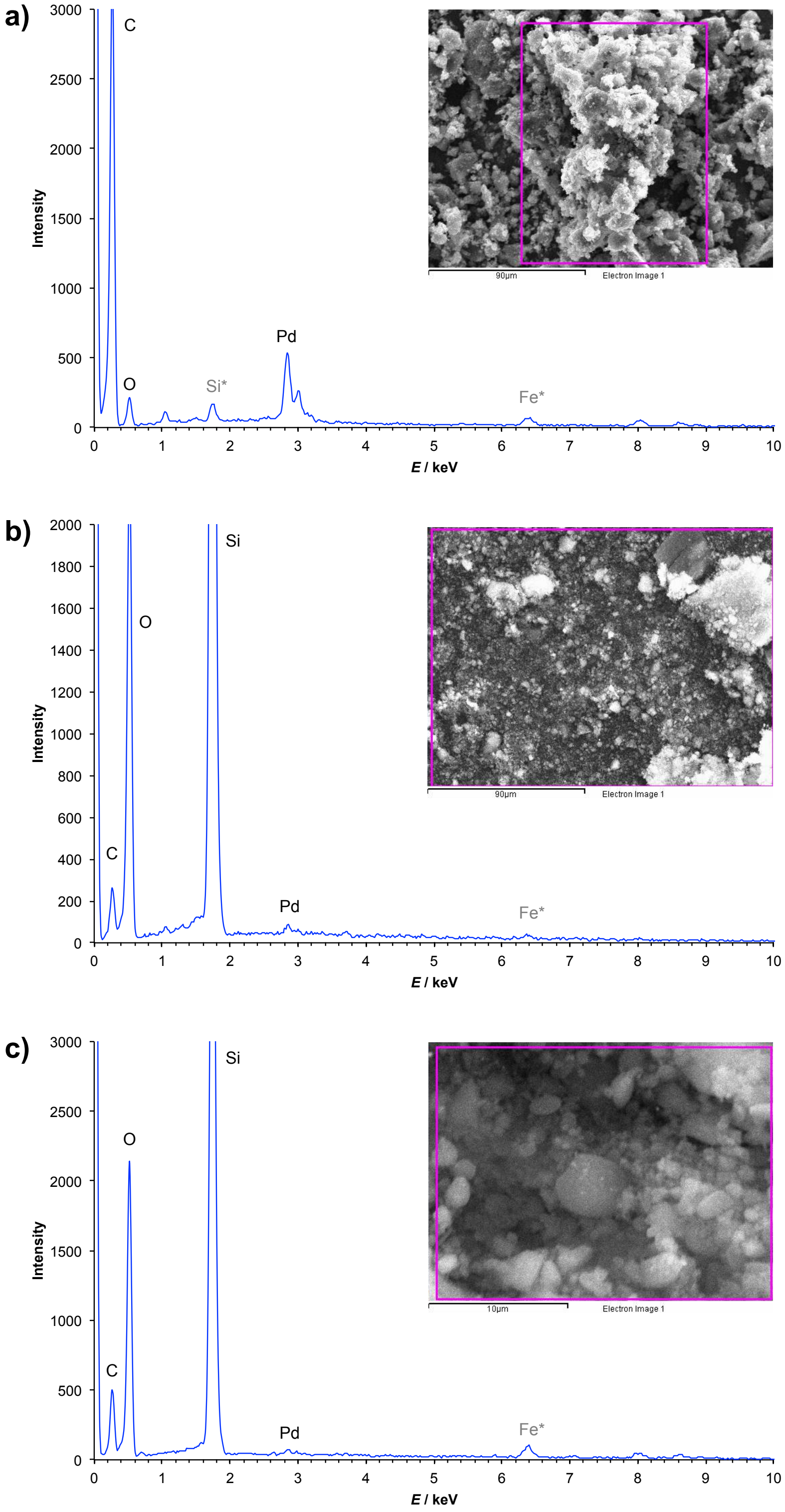

Figure S9. SEM-EDS analysis of a) commercial Pd/C catalyst (10 wt%) milled for 60 mintues at 30 Hz using a single 12 mm (7.0 g) stainless steel ball shows a homogeneous distribution of Pd over the entire selected area, b) Pd/C catalyst milled in the presence of silica under LAG conditions for 60 minutes, c) a post-workup sample of the mixture containing Pd/C and silica after LAG reduction of **1a** to **2a** for 60 minutes. The intensity of Pd signal in EDS spectra under b) and c) is reduced because of dilution effect by added silica. All samples contain iron contamination (Fe*) due to abrasion of stainless steel jar walls and the ball during milling.

6. MS and GC analysis of CTH of 4-chloro-2-nitroaniline (*o*-1p) and 2-chloro-4-nitroaniline (*p*-1p)

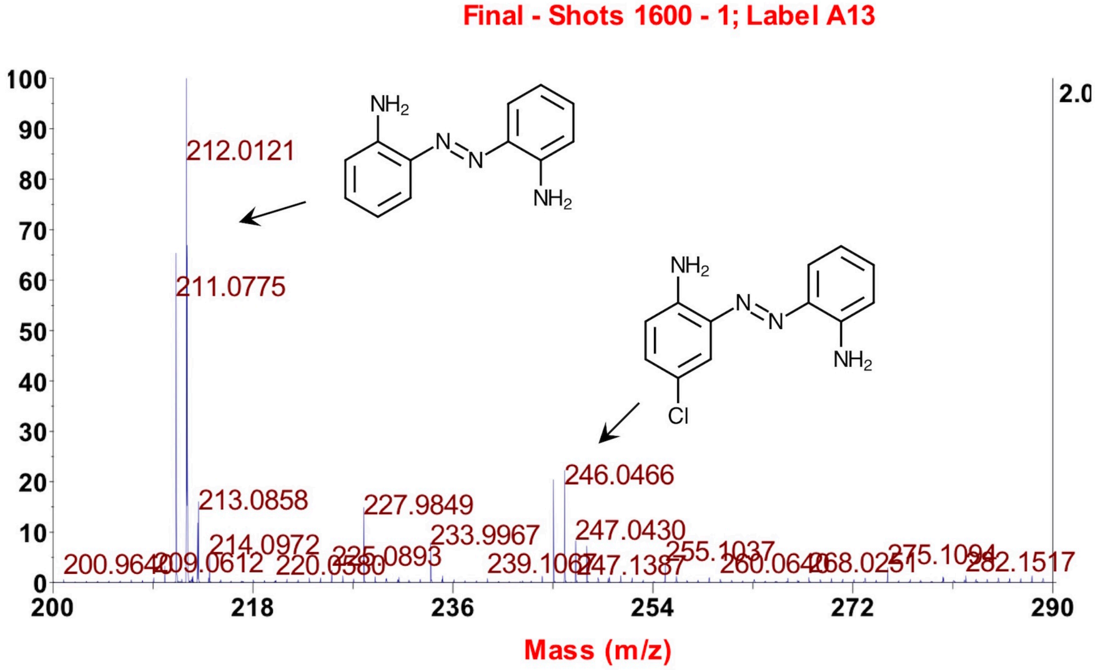

Figure S10. MS analysis of the methanol fraction after CTH of 4-chloro-2-nitroaniline (***o*-1p**) under standard conditions. 2,2'-Diaminoazobenzene (M_r = 212) and 2,2'-diamino-4-chloroazobenzene (M_r = 246) were identified as intermediates in the reaction mixture, which was consistent with the observed diminished yield (42%) of 4-chloro-*o*-phenylenediamine (***o*-2p**) due to dehalogenation side reaction.

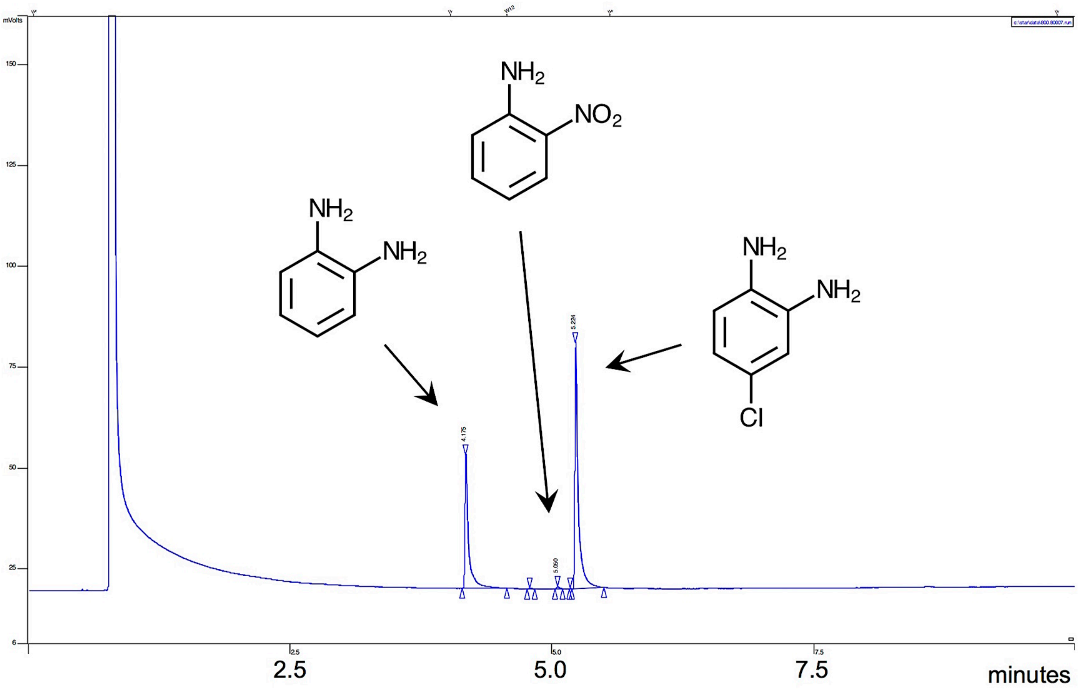

Figure S11. GC analysis of the crude mixture after CTH of 4-chloro-2-nitroaniline (***o*-1p**). *o*-Phenylenediamine (t_R = 4.18 min) and *o*-nitroaniline (t_R = 5.06 min) are the by-products of dehalogenation side reaction. The reactant was consumed completely while the signal at t_R = 5.22 min corresponds to 4-chloro-*o*-phenylenediamine (***o*-2p**) product.

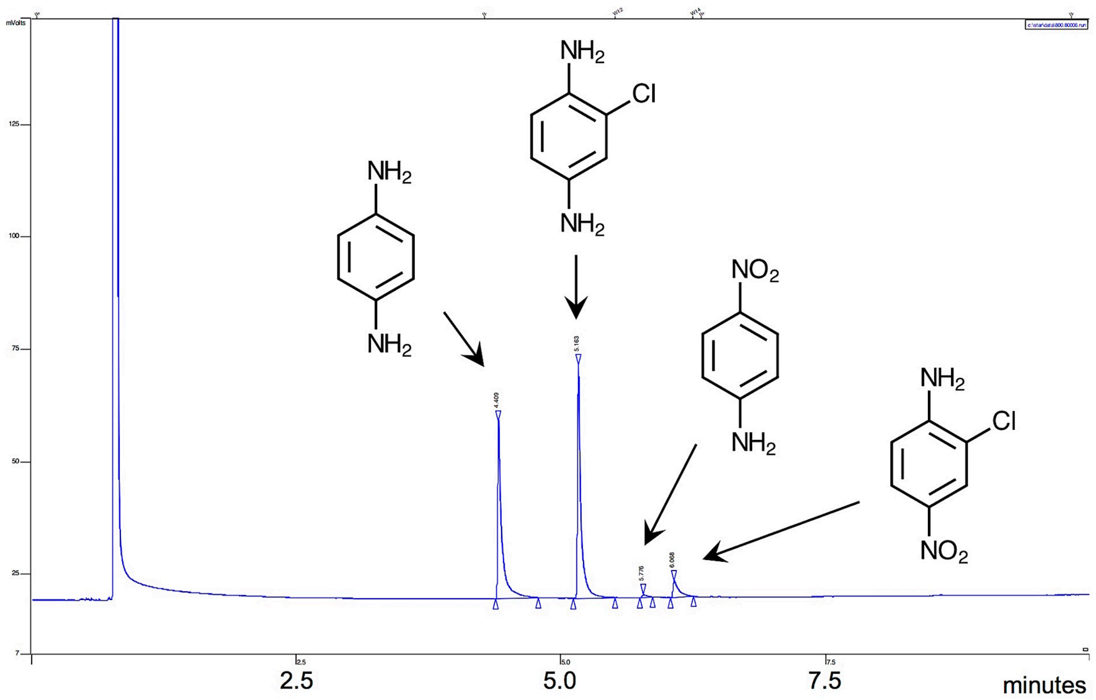

Figure S12. GC analysis of the crude mixture after CTH of 2-chloro-4-nitroaniline (***p*-1p**). *p*-Phenylenediamine (t_R = 4.41 min) and *p*-nitroaniline (t_R = 5.77 min) are the by-products of dehalogenation side reaction. The reactant (t_R = 6.06 min) was not consumed completely while the signal at t_R = 5.16 min corresponds to 2-chloro-*p*-phenylenediamine (***p*-2p**) product.

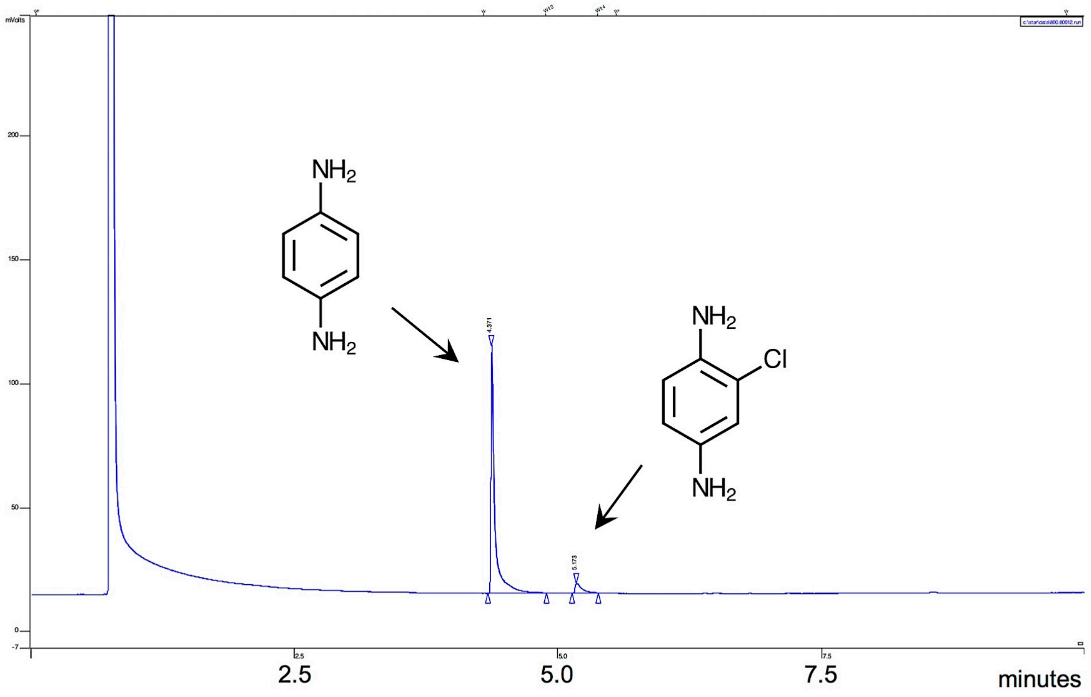

Figure S13. GC analysis of the crude mixture after CTH of 2-chloro-4-nitroaniline (***p*-1p**) with 2.2 eq of ammonium formate (6.6 mmol). *p*-Phenylenediamine (t_R = 4.41 min) and small amount of 2-chloro-*p*-phenylenediamine (***p*-2p**) were found as the only products, suggesting that dehalogenation became the primary reaction pathway.

7. ^{1}H and ^{13}C NMR spectra of selected compounds

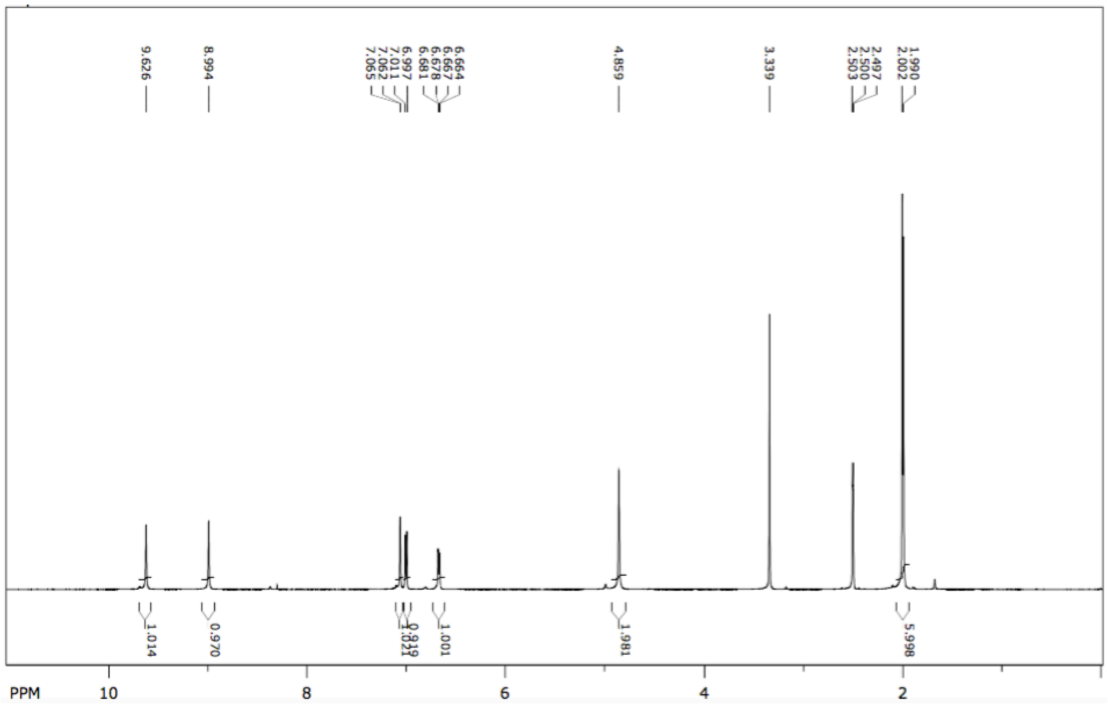

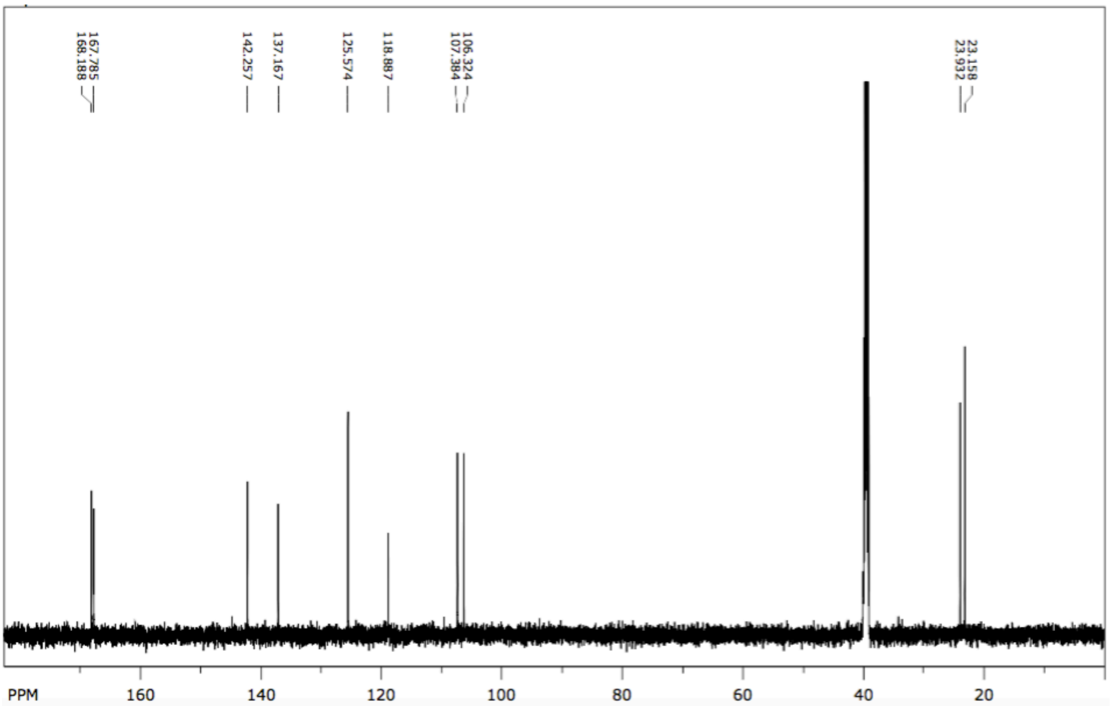

Figure S14. ^{1}H and ^{13}C NMR spectra of **2g**.

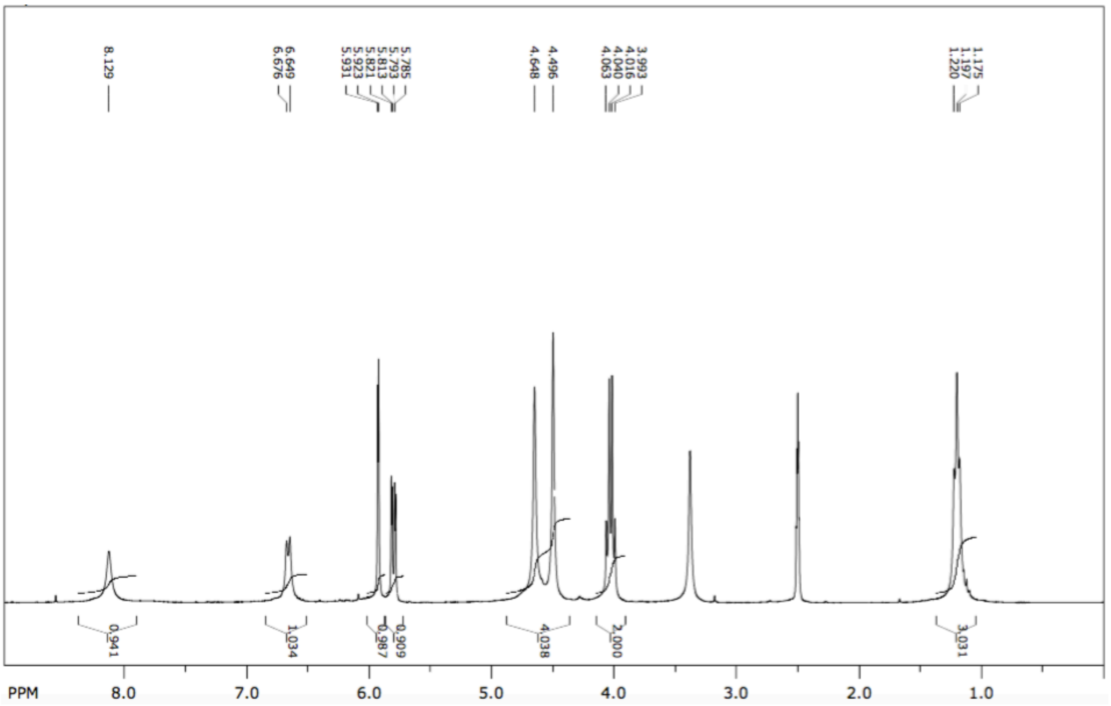

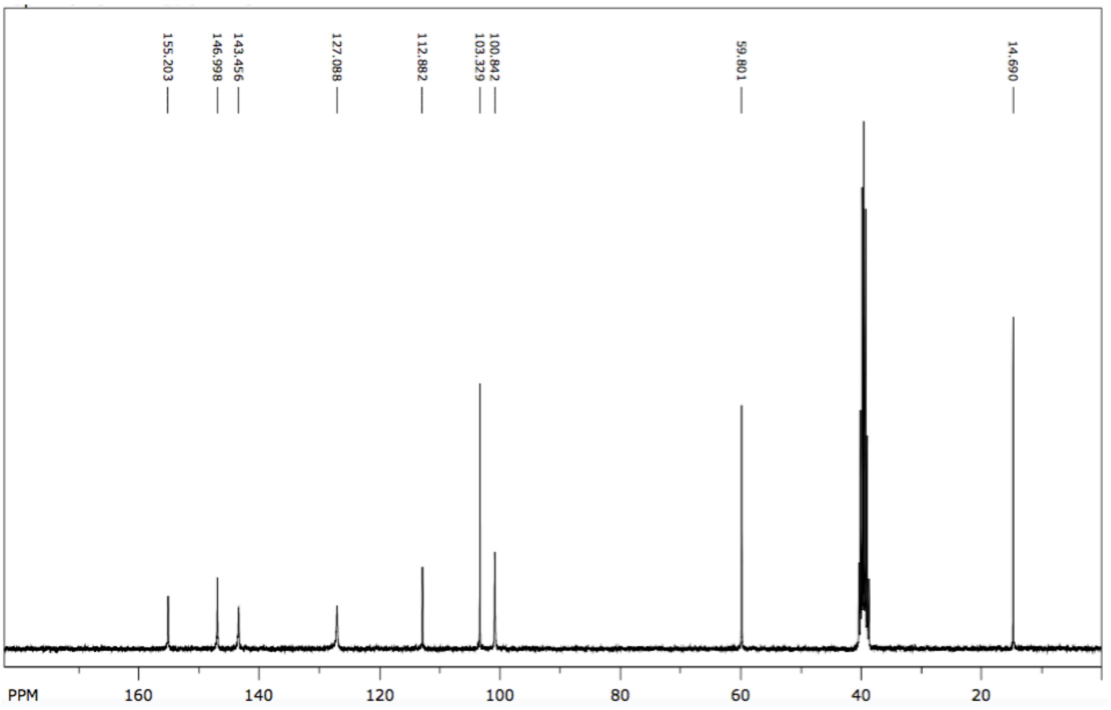

Figure S15. ^{1}H and ^{13}C NMR spectra of **2h**.

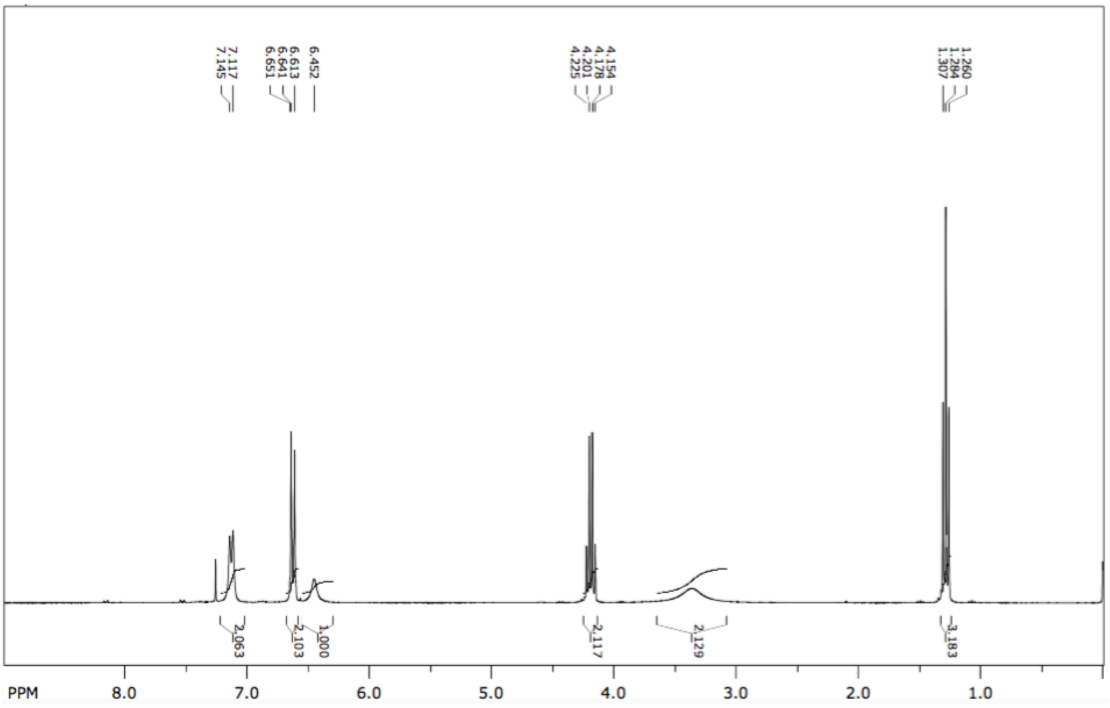

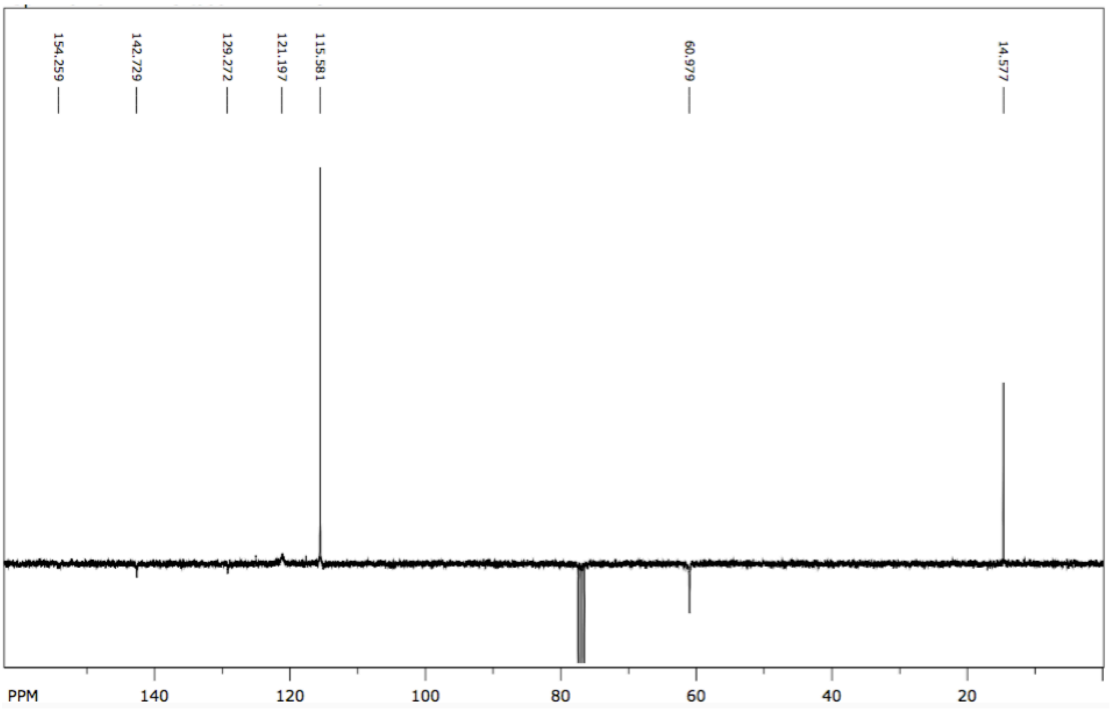

Figure S16. ^{1}H and ^{13}C NMR spectra of **2i**.

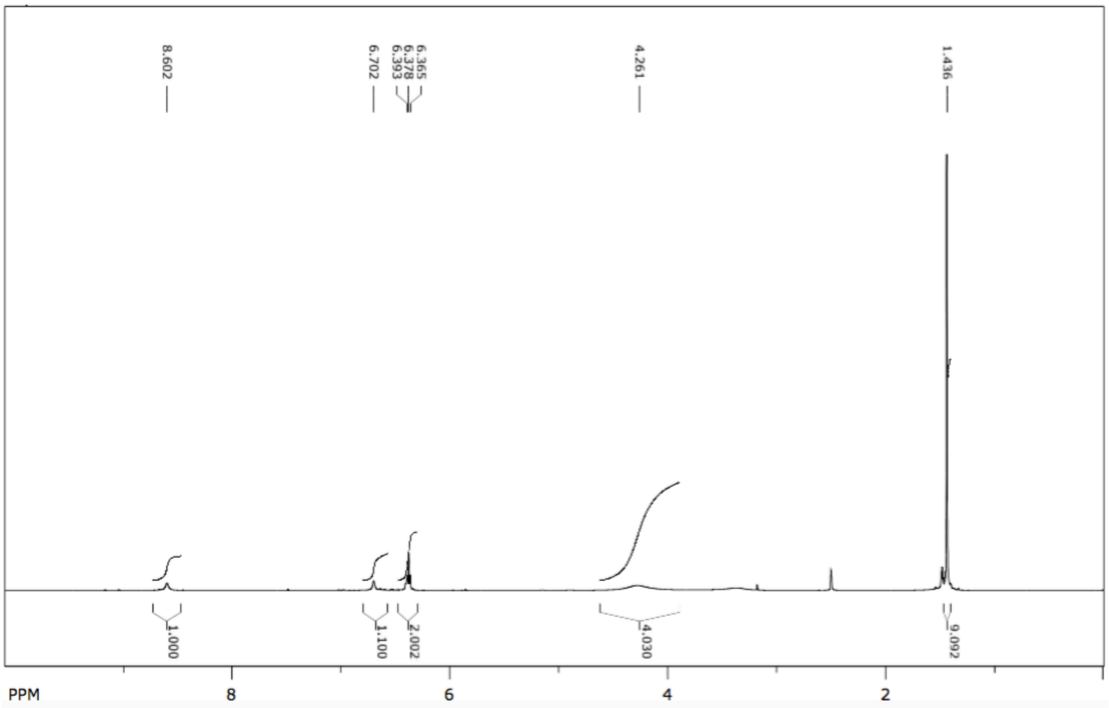

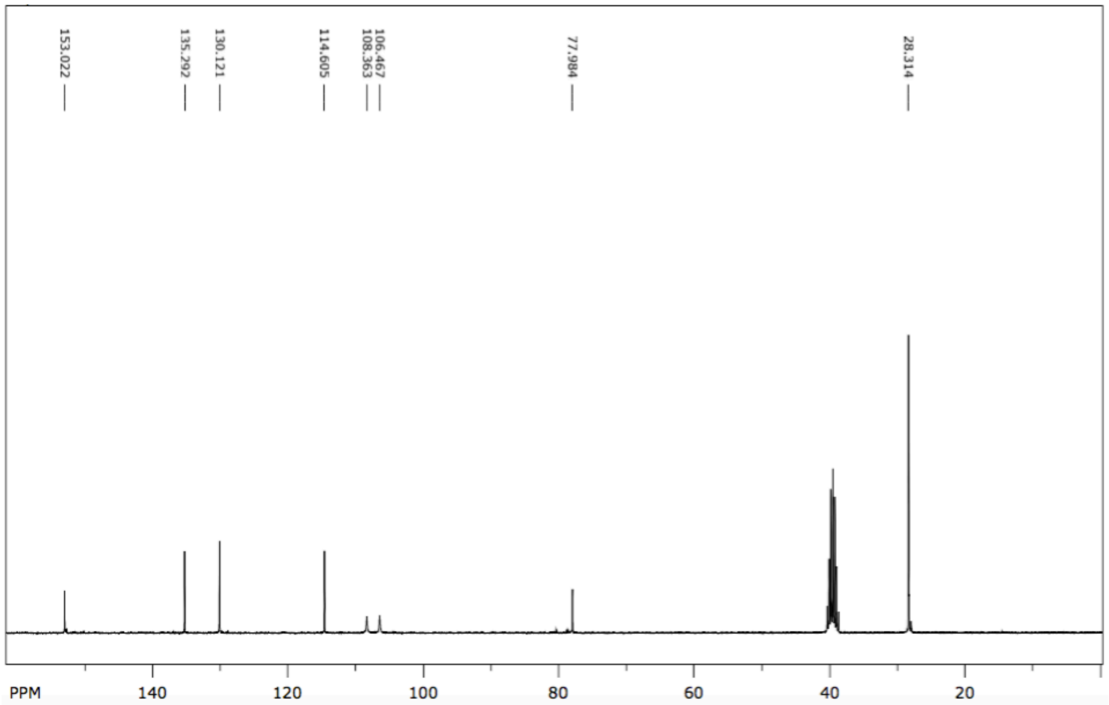

Figure S17. ^{1}H and ^{13}C NMR spectra of **2j**.

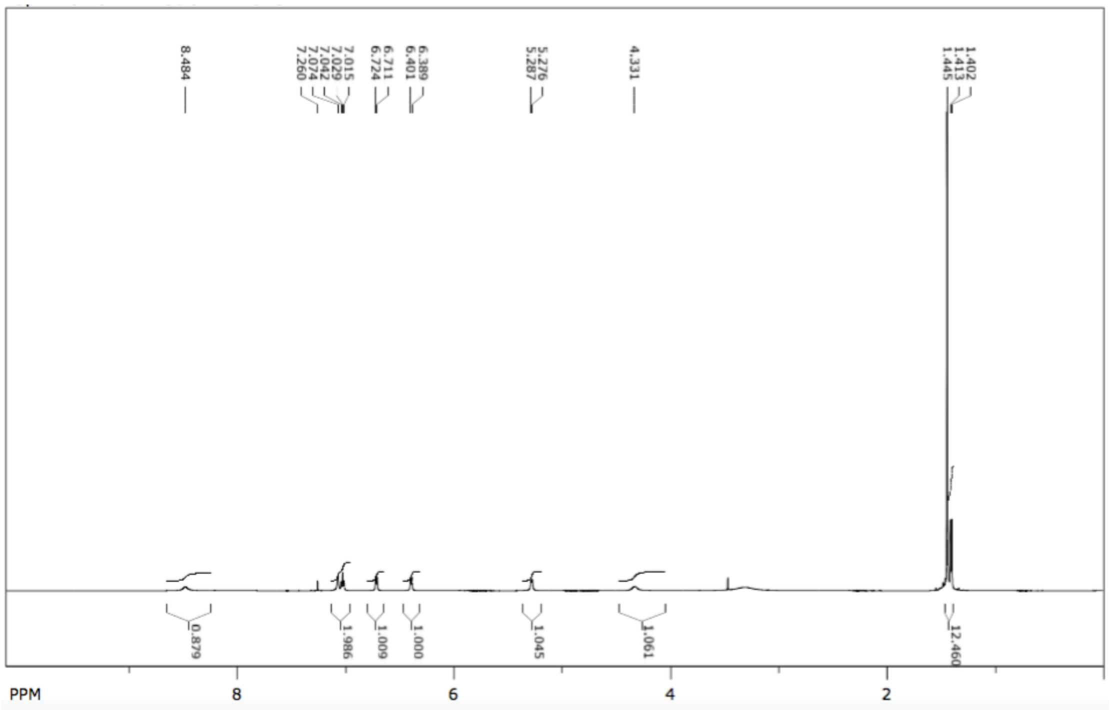

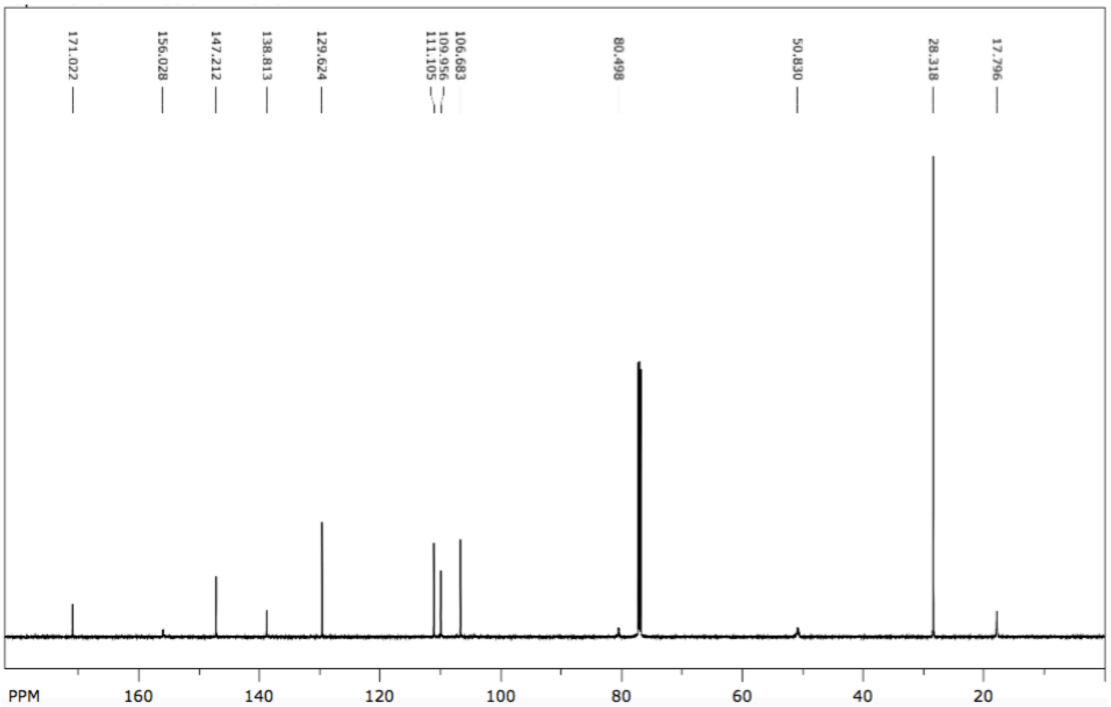

Figure S18. ^{1}H and ^{13}C NMR spectra of **2k**.

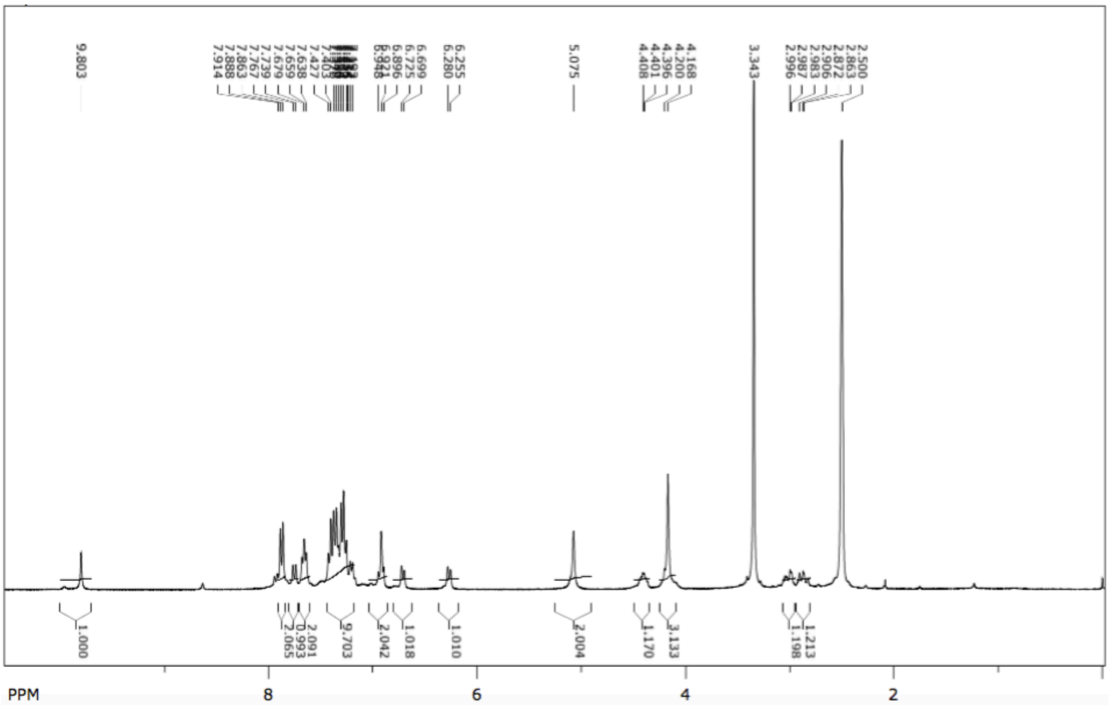

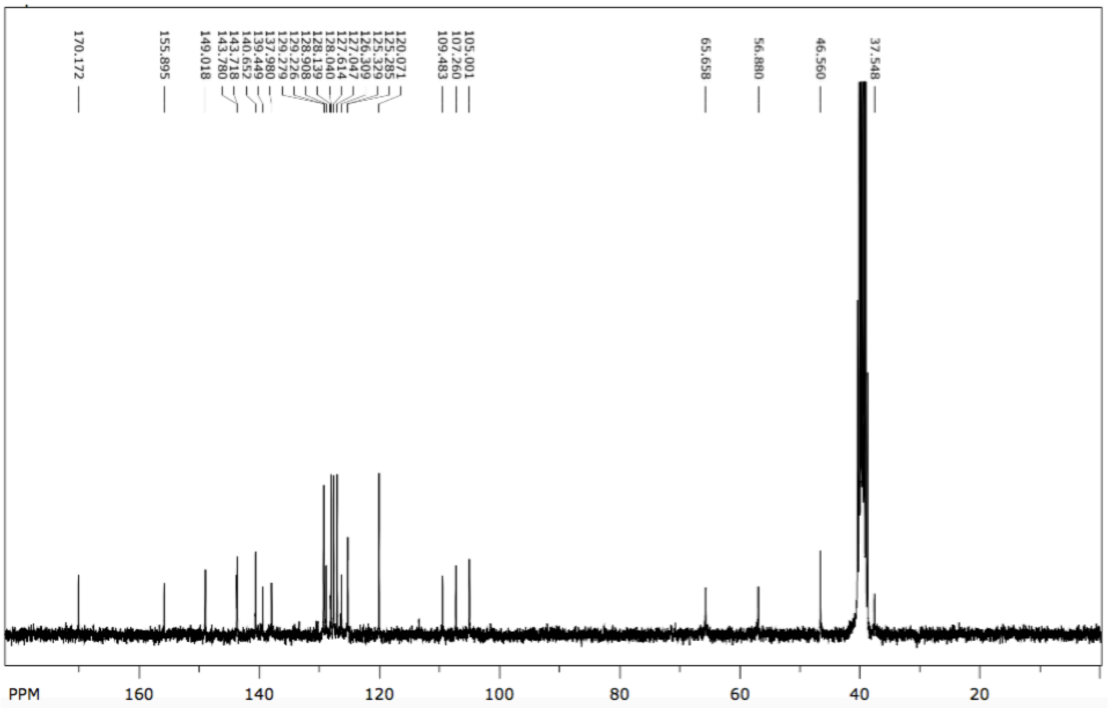

Figure S19. ^{1}H and ^{13}C NMR spectra of **2l-Phe**.

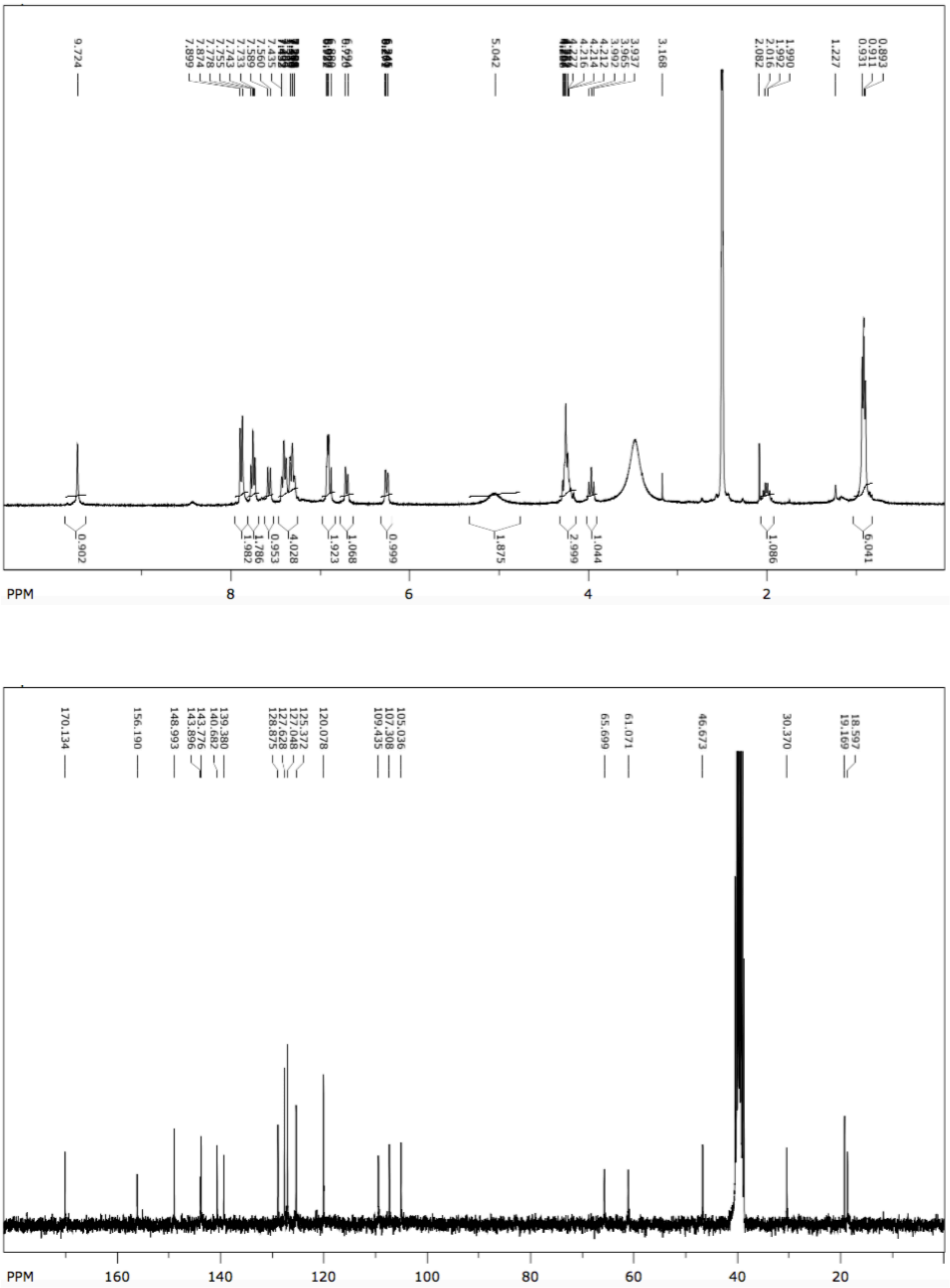

Figure S20. ^{1}H and ^{13}C NMR spectra of **2l-Val**.

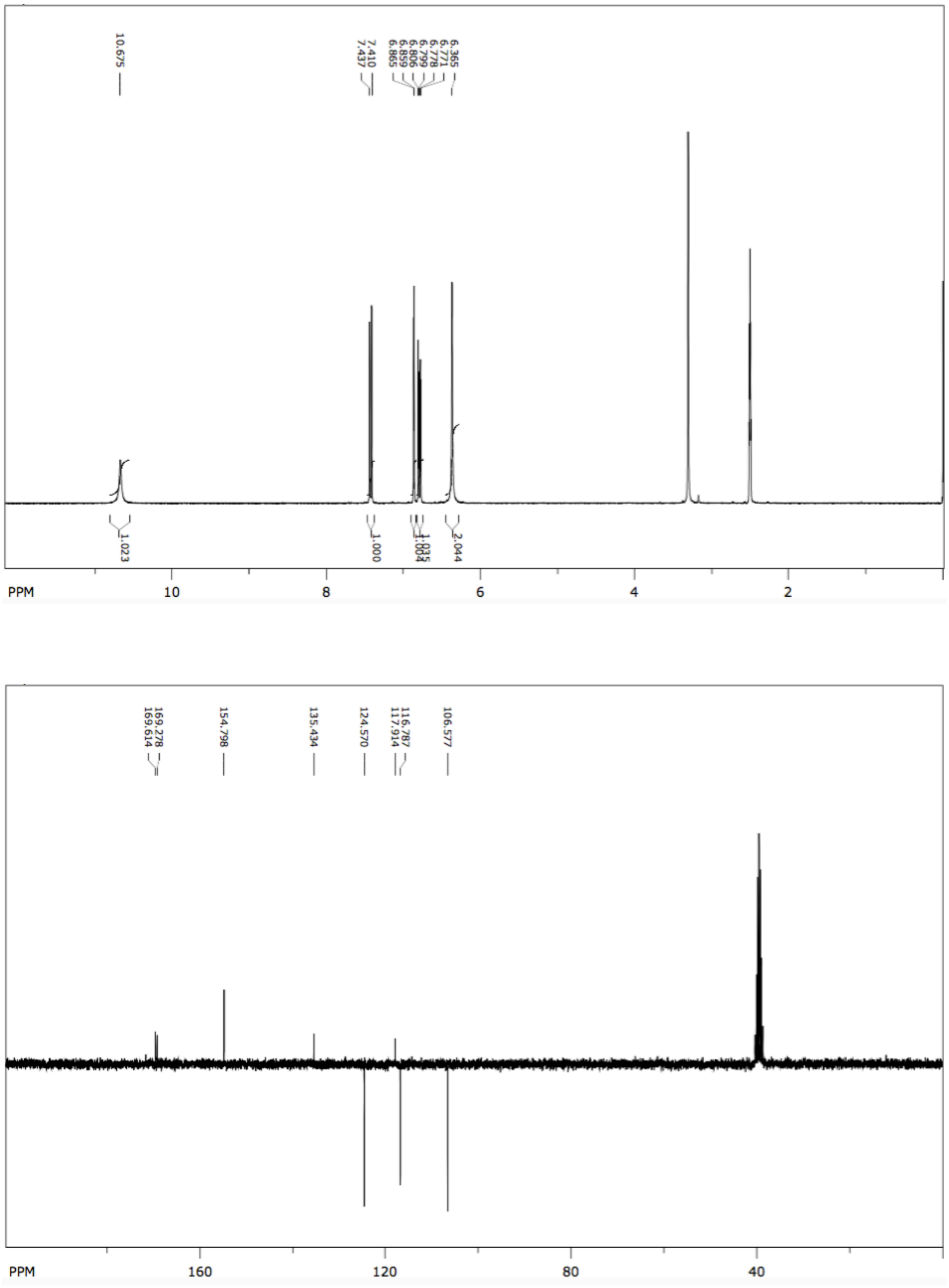

Figure S21. ^{1}H and ^{13}C NMR spectra of **2m**.

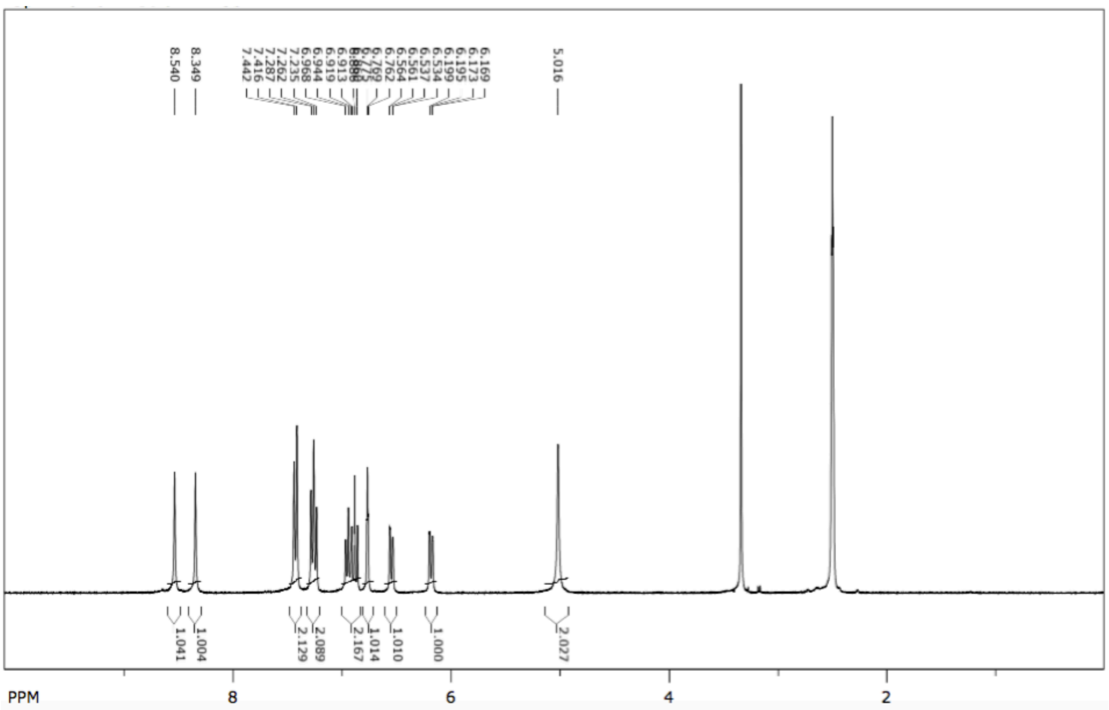

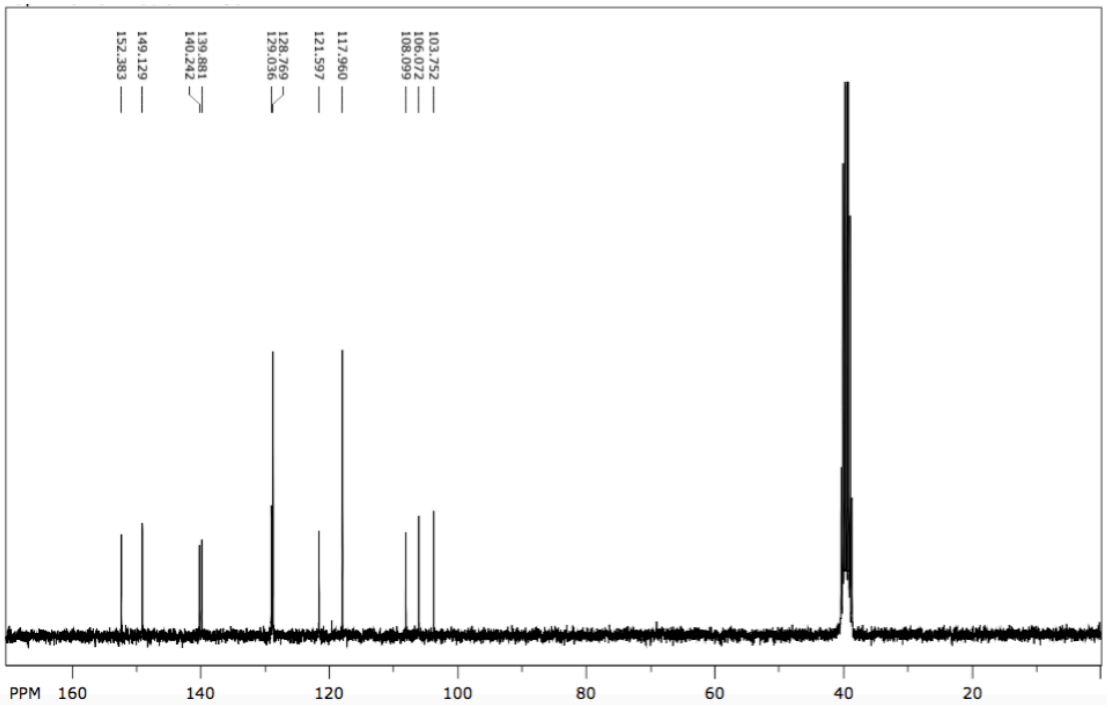

Figure S22. ^{1}H and ^{13}C NMR spectra of **2n-O**.

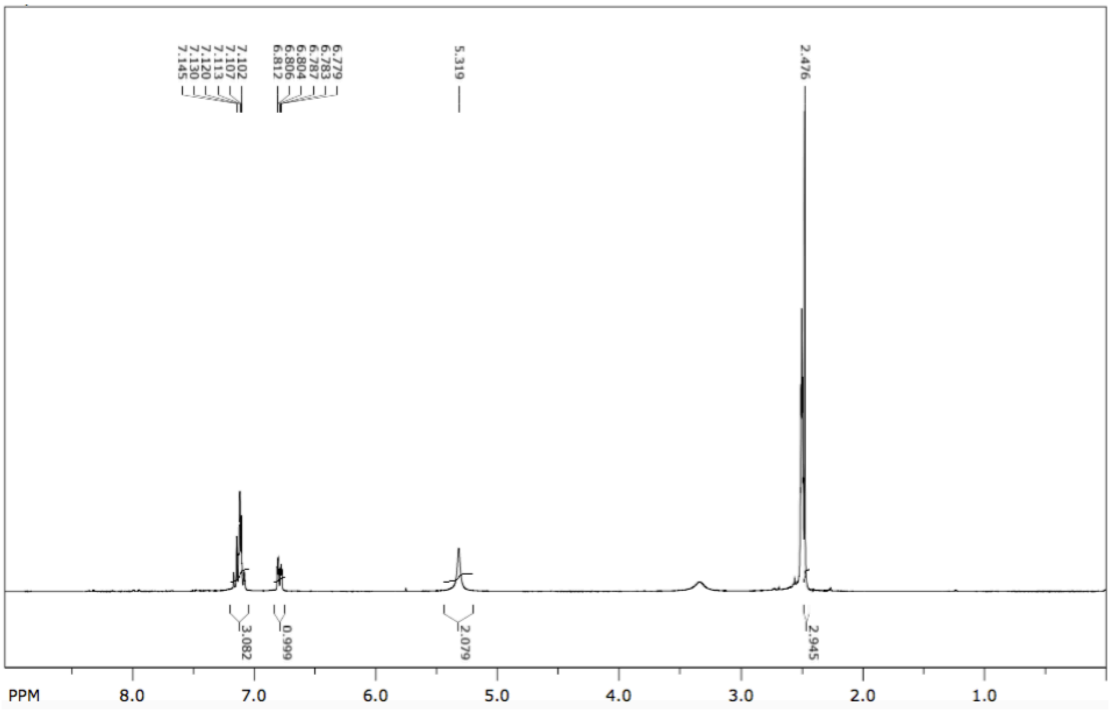

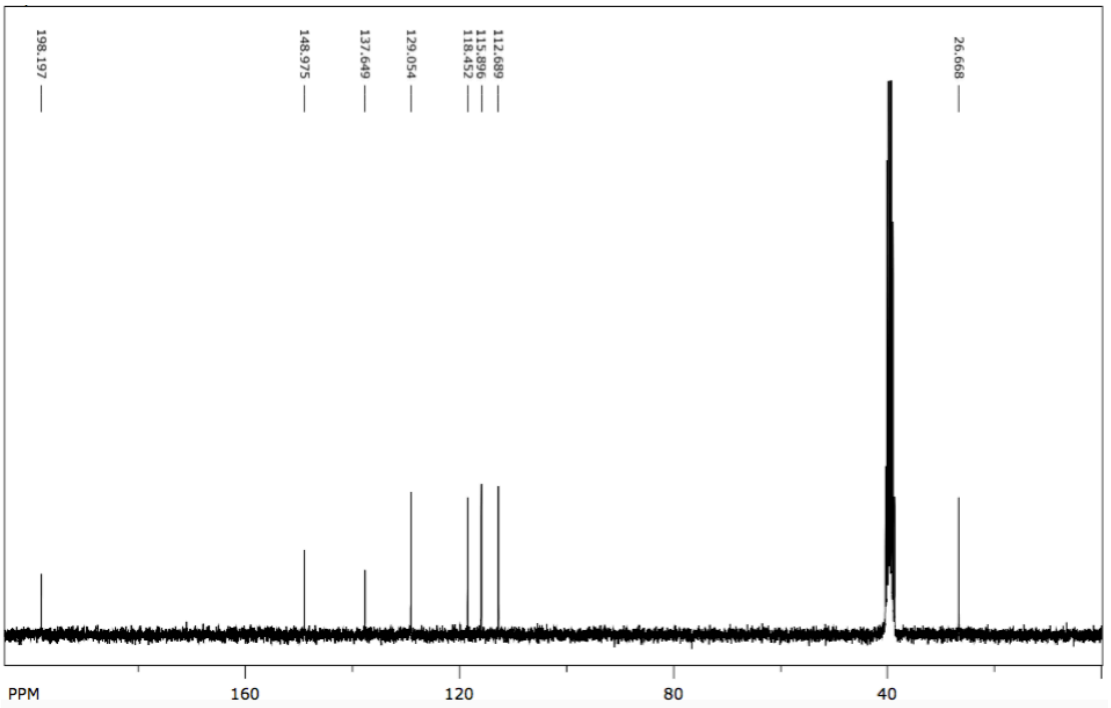

Figure S23. ^{1}H and ^{13}C NMR spectra of **2o**.

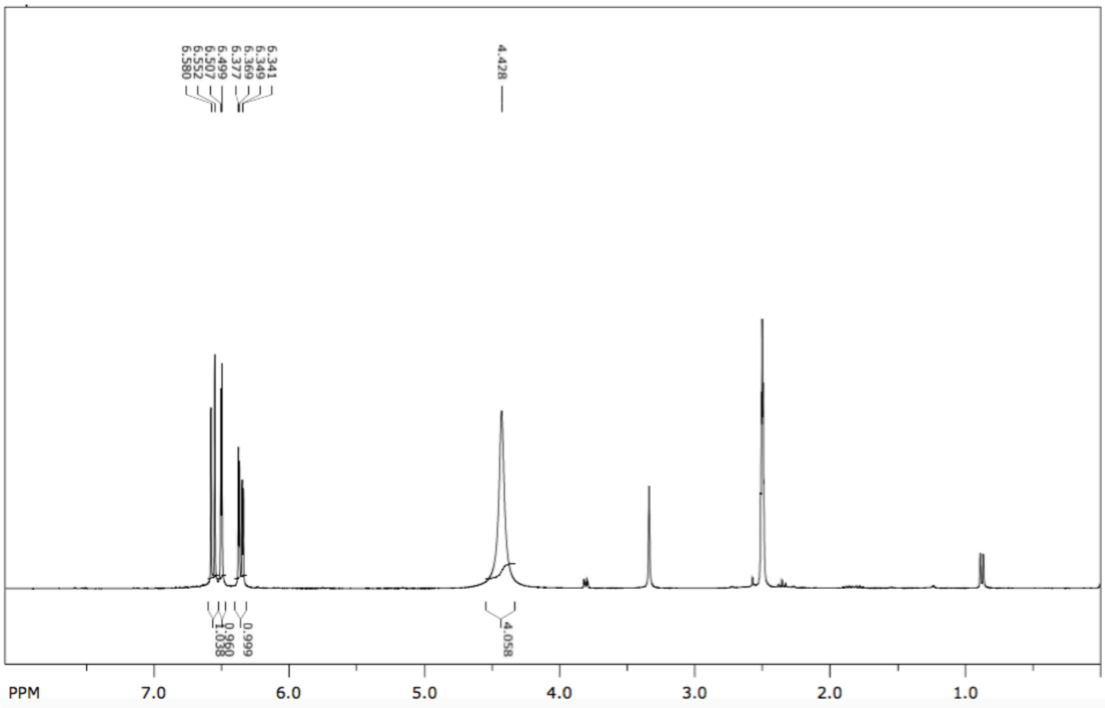

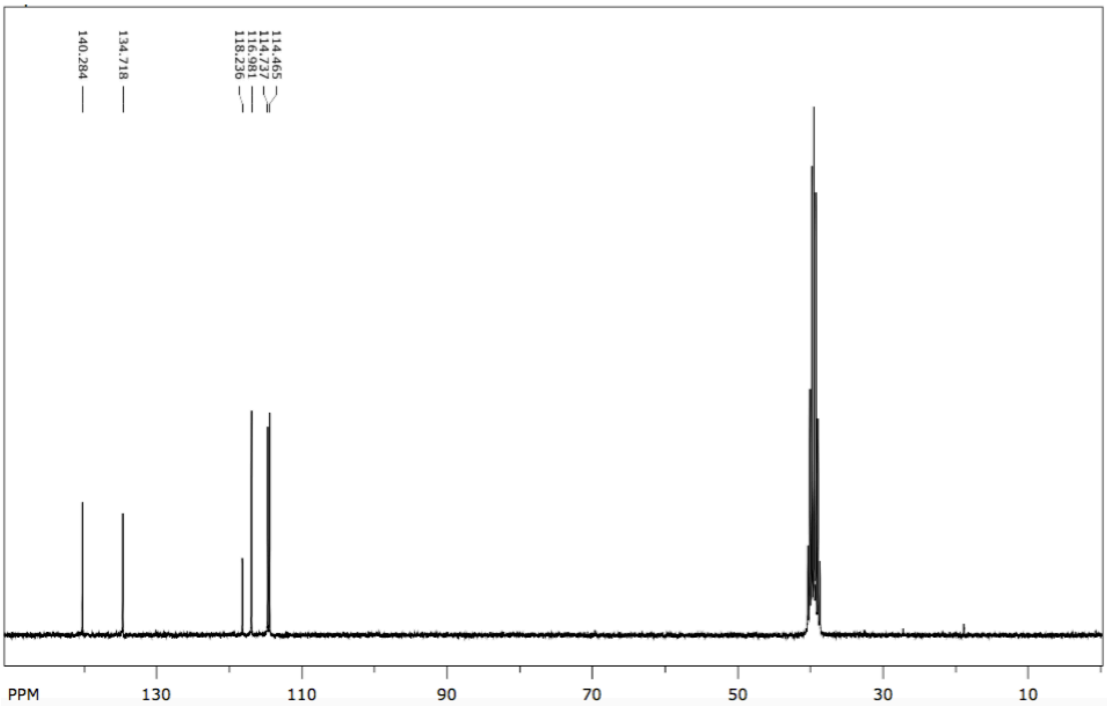

Figure S24. ^{1}H and ^{13}C NMR spectra of ***p*-2p**.

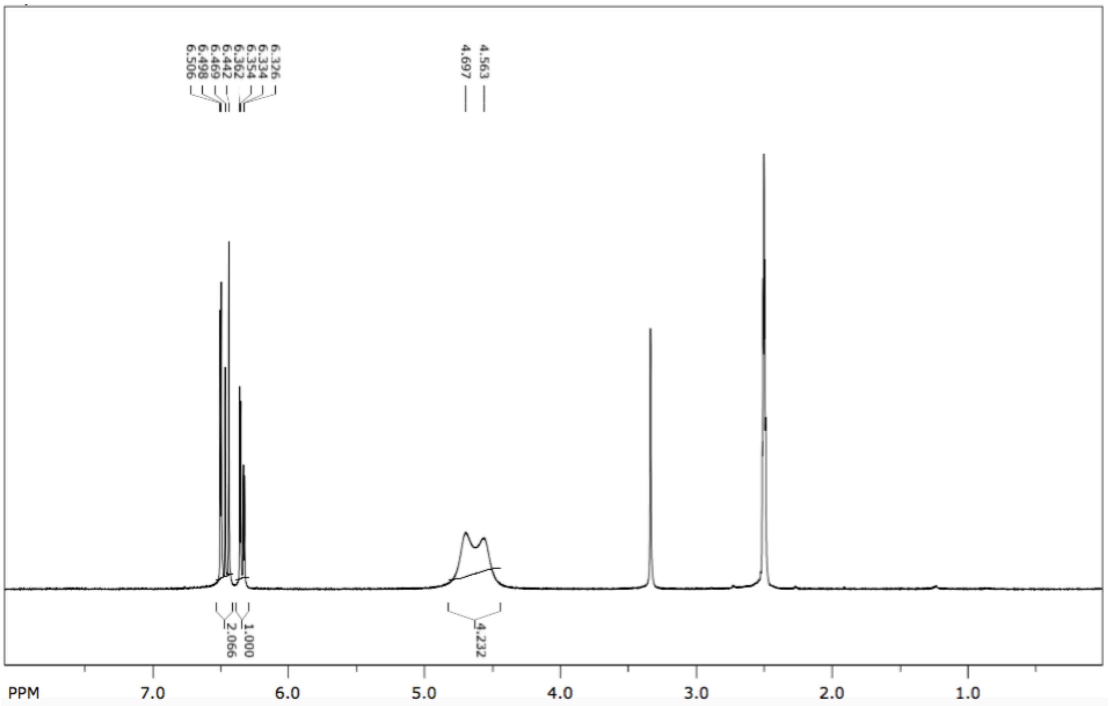

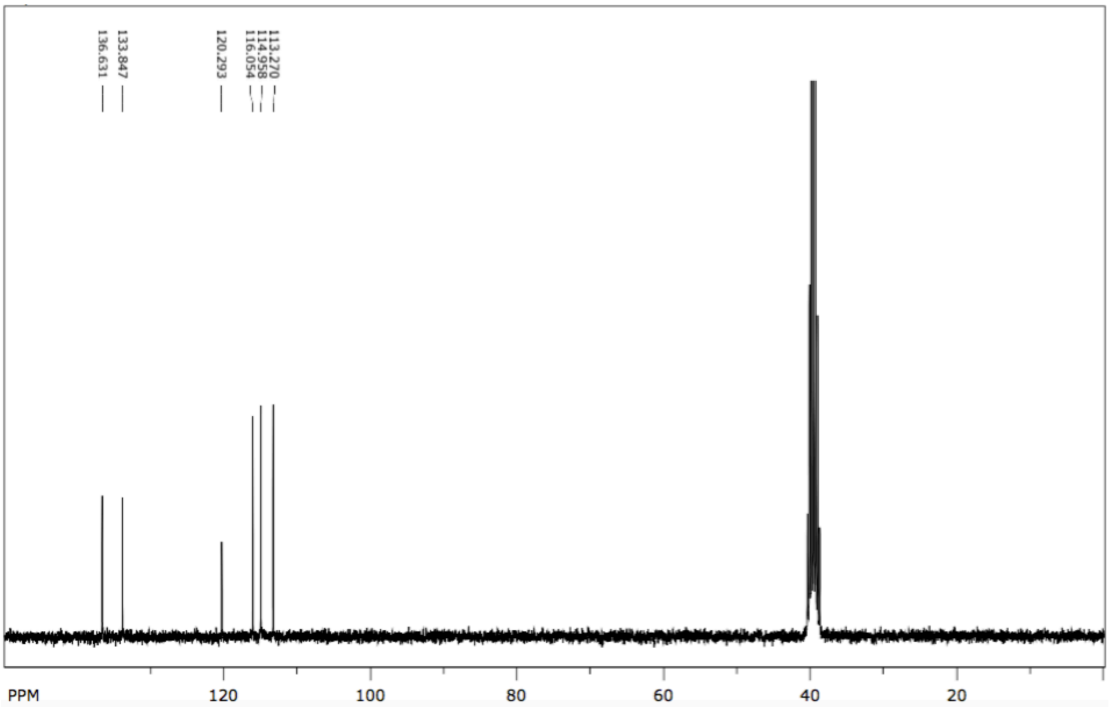

Figure S25. ^{1}H and ^{13}C NMR spectra of ***o*-2p**.

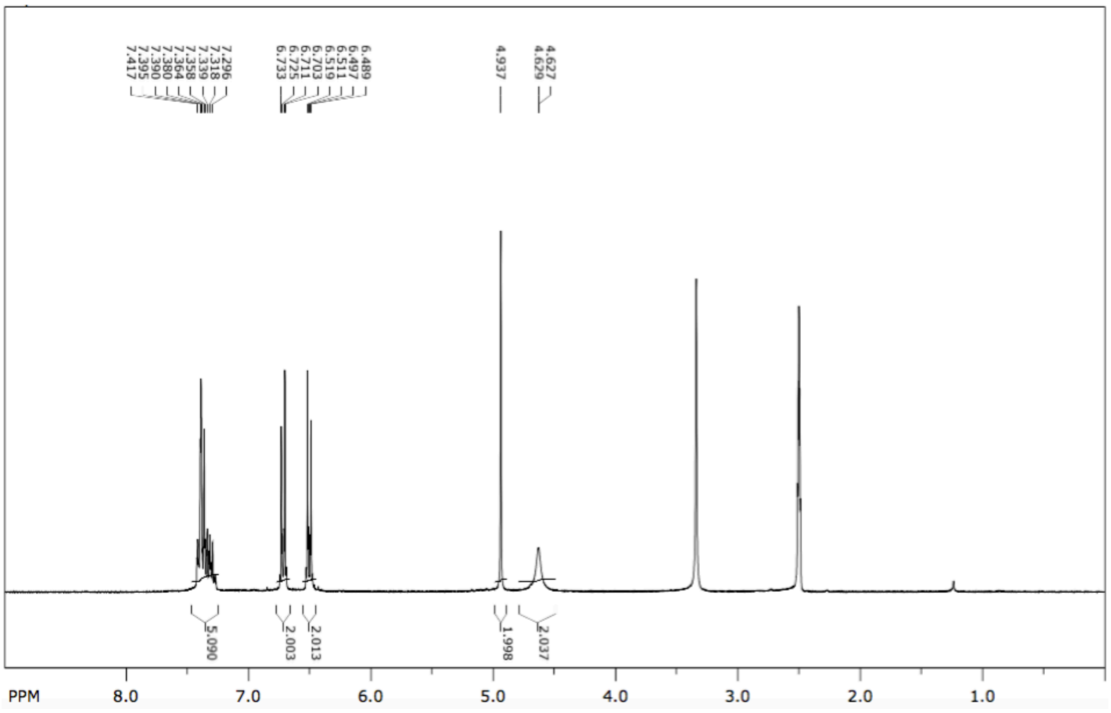

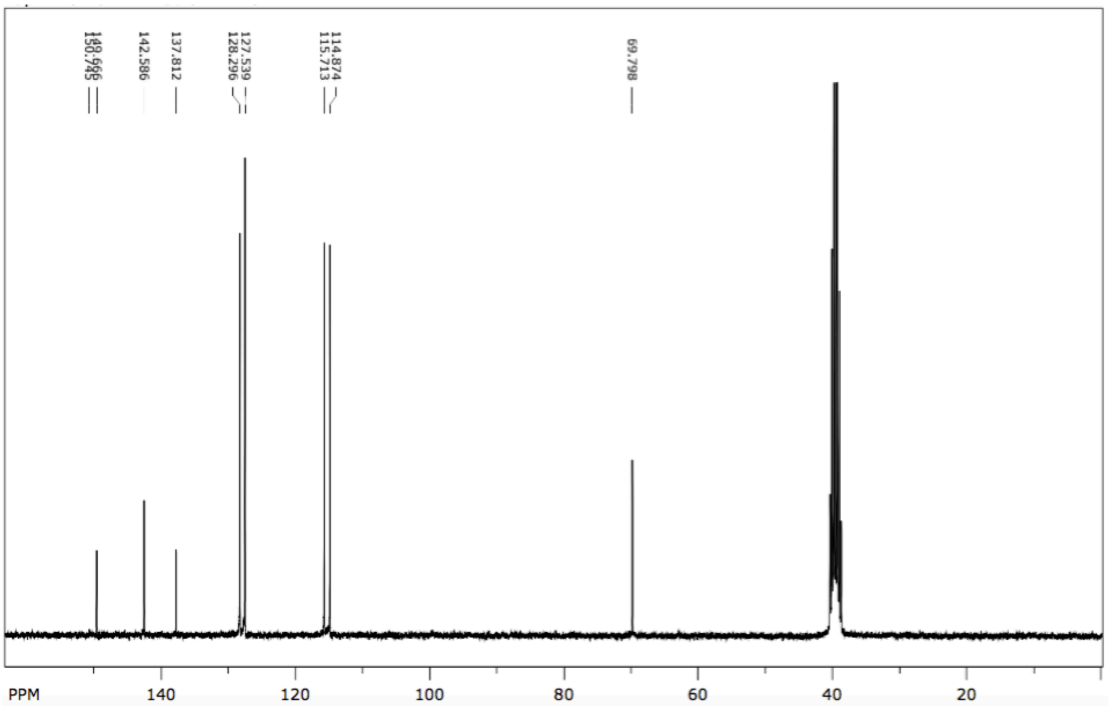

Figure S26. ^{1}H and ^{13}C NMR spectra of **2q**.

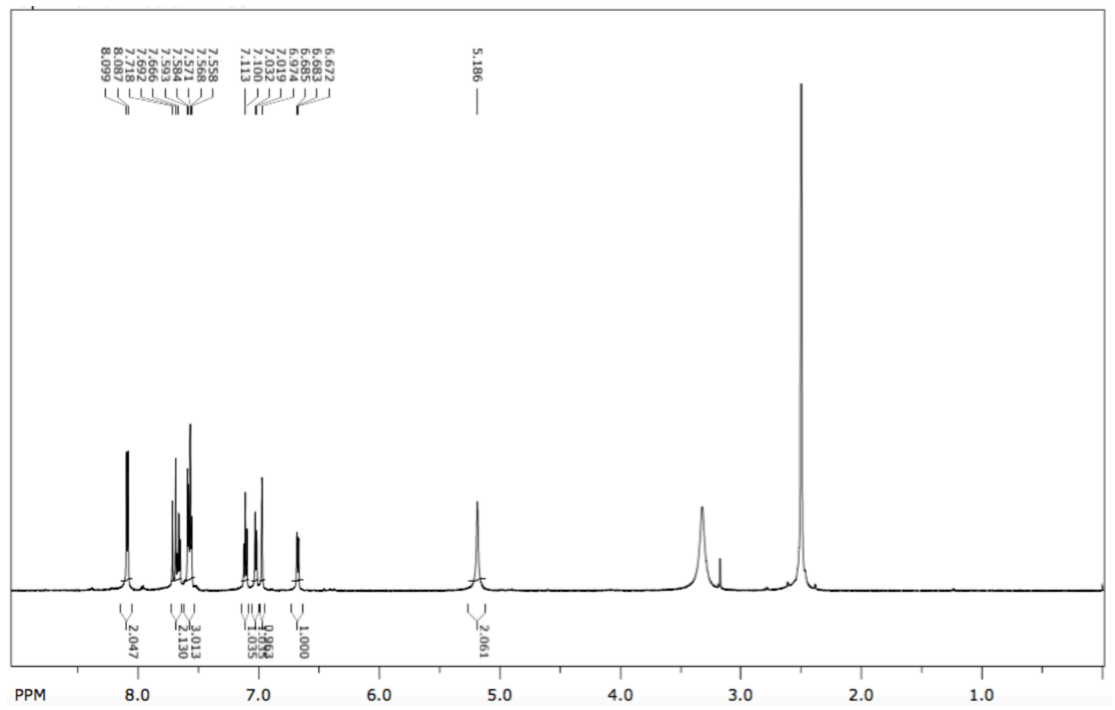

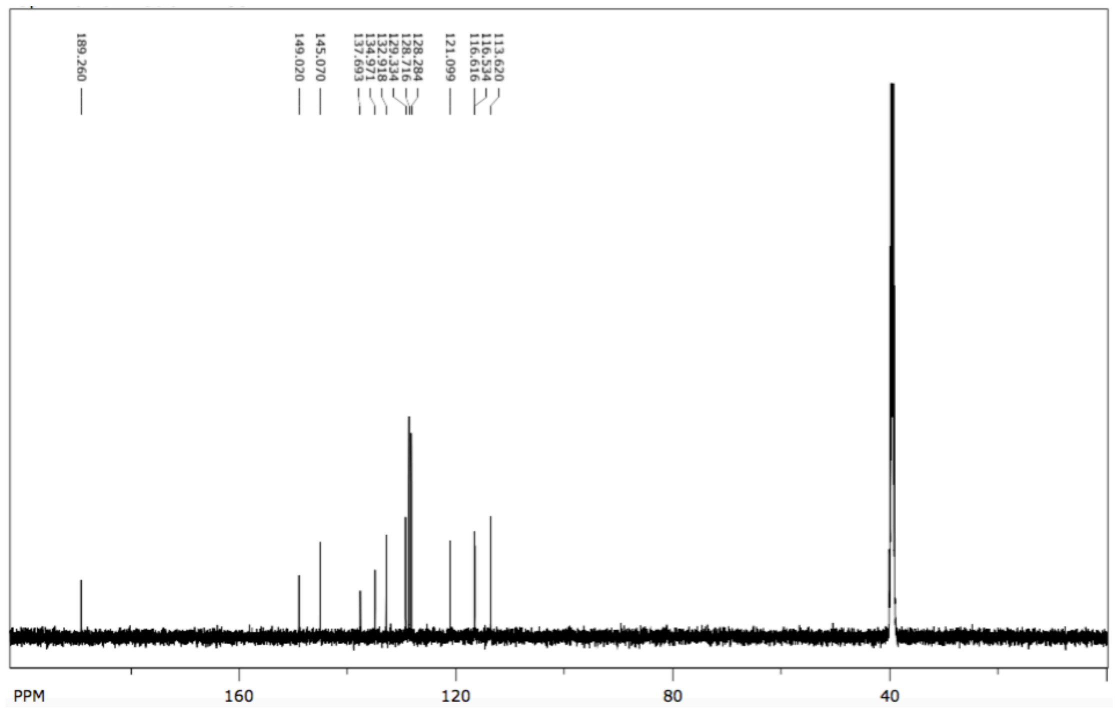

Figure S27. ^{1}H and ^{13}C NMR spectra of ***m*-2r**.

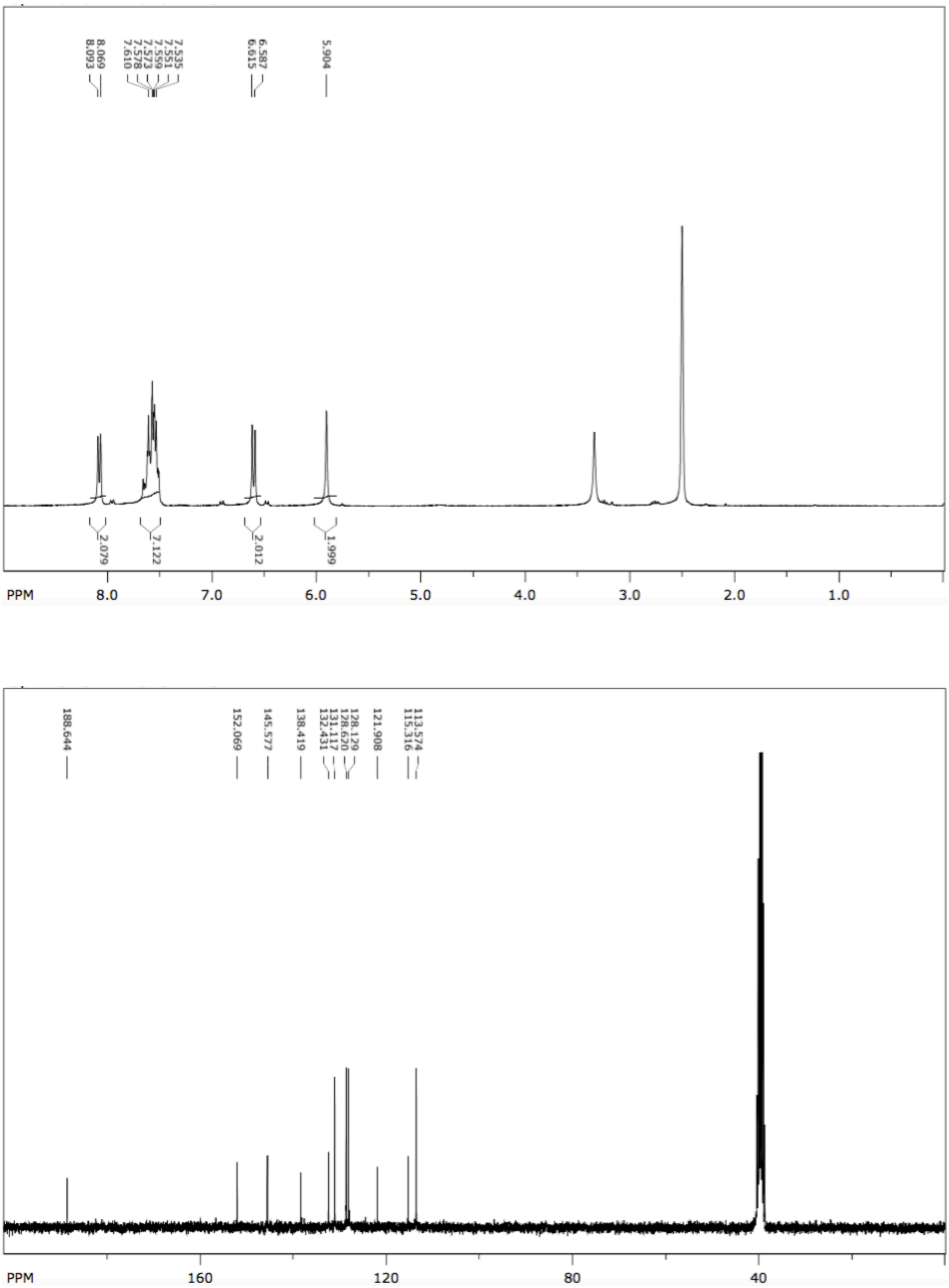

Figure S28. ^{1}H and ^{13}C NMR spectra of ***p*-2r**.

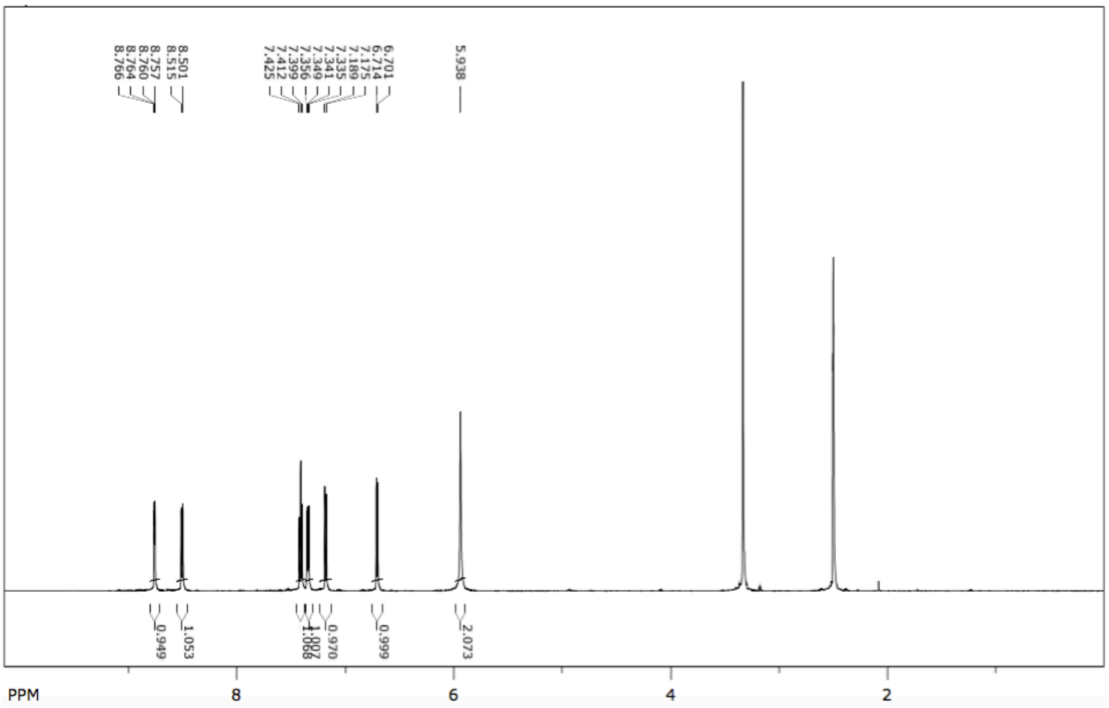

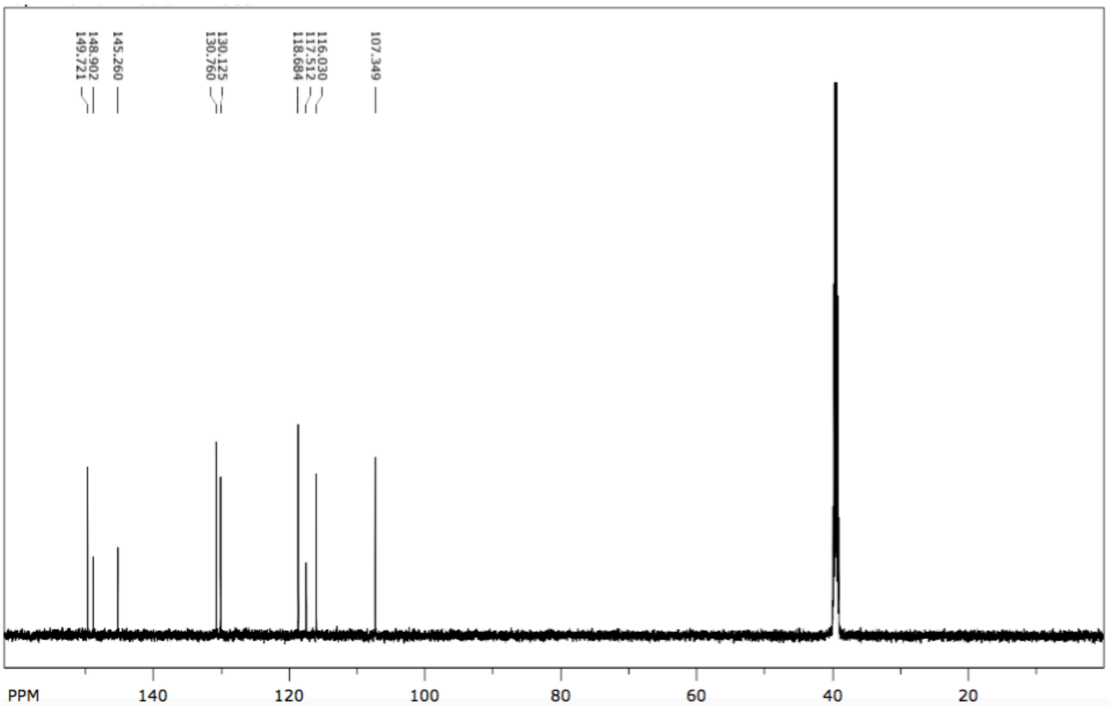

Figure S29. ^{1}H and ^{13}C NMR spectra of **2t**.

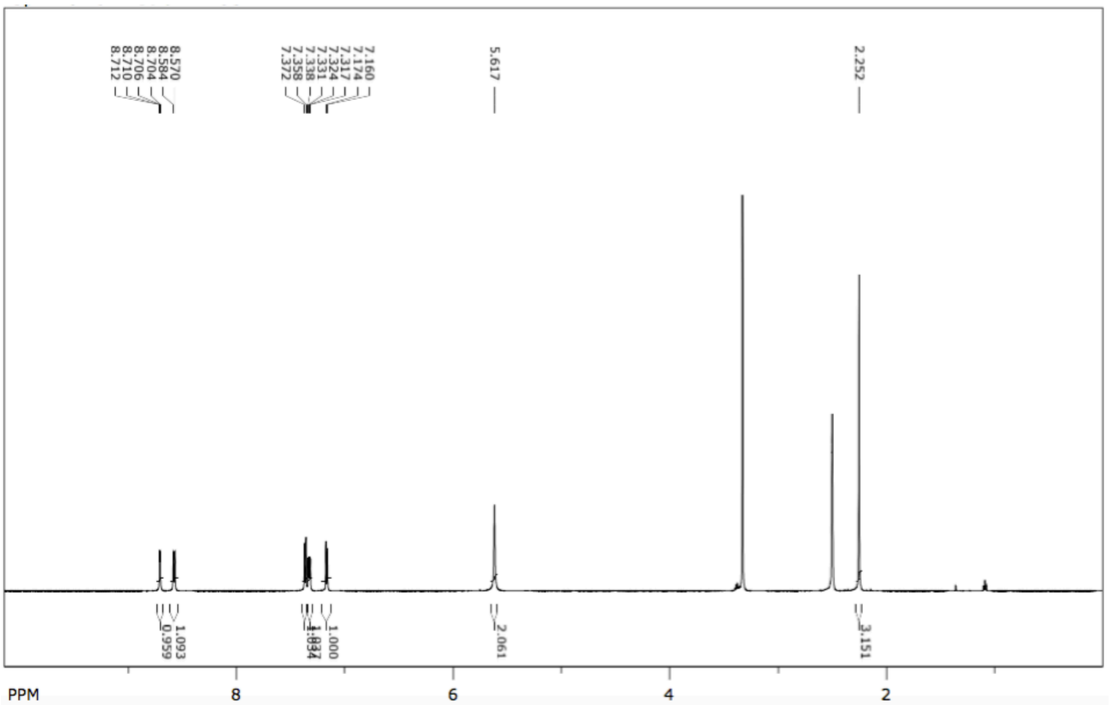

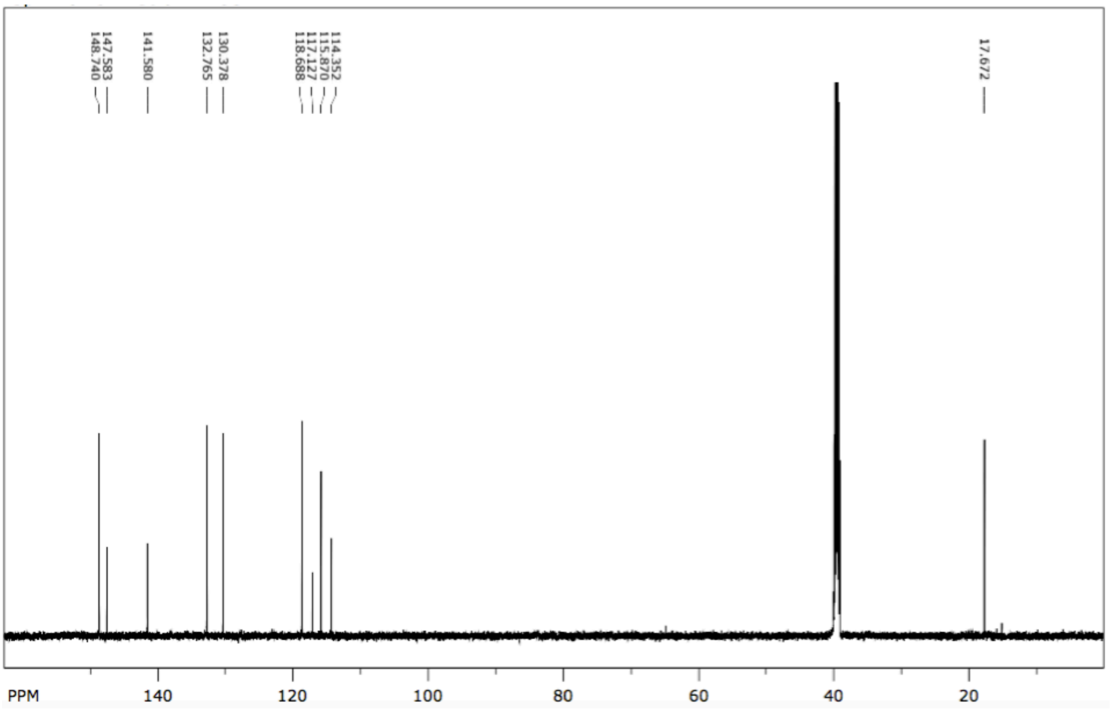

Figure S30. ^{1}H and ^{13}C NMR spectra of **2u**.

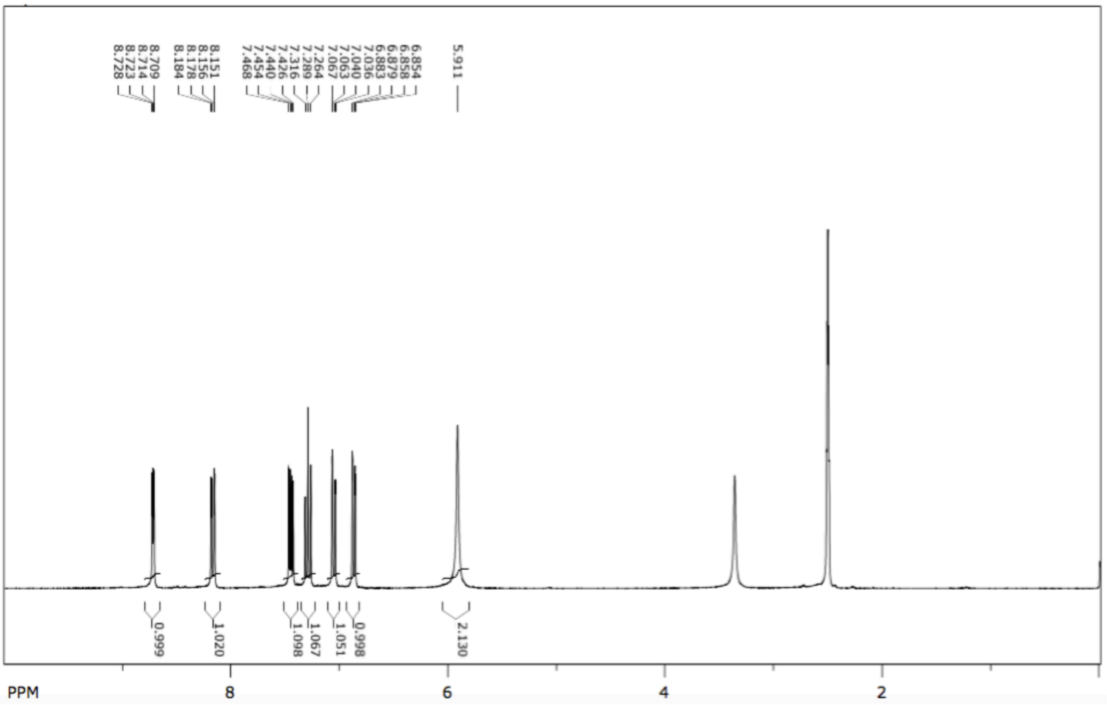

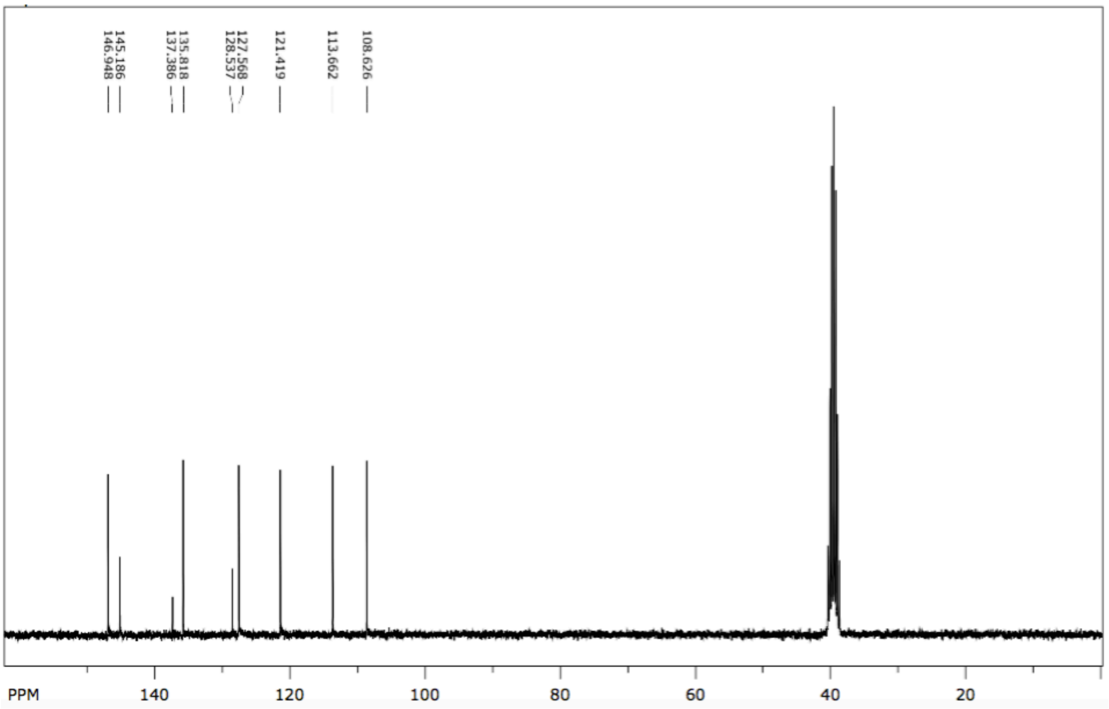

Figure S31. ^{1}H and ^{13}C NMR spectra of **2v**.

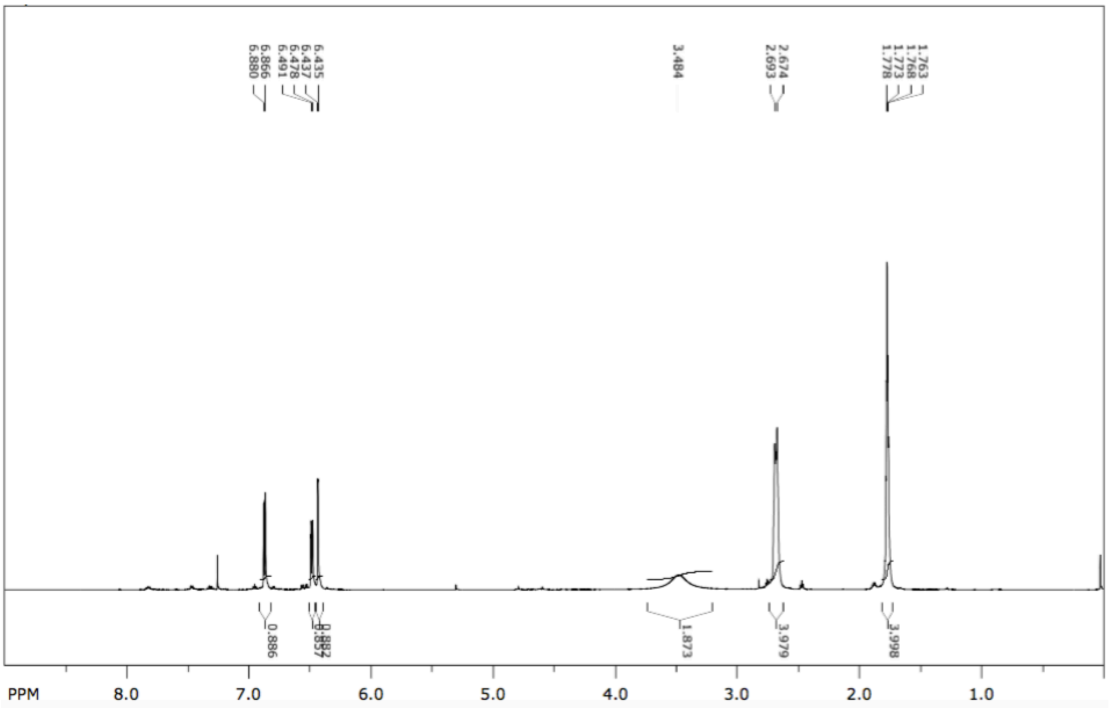

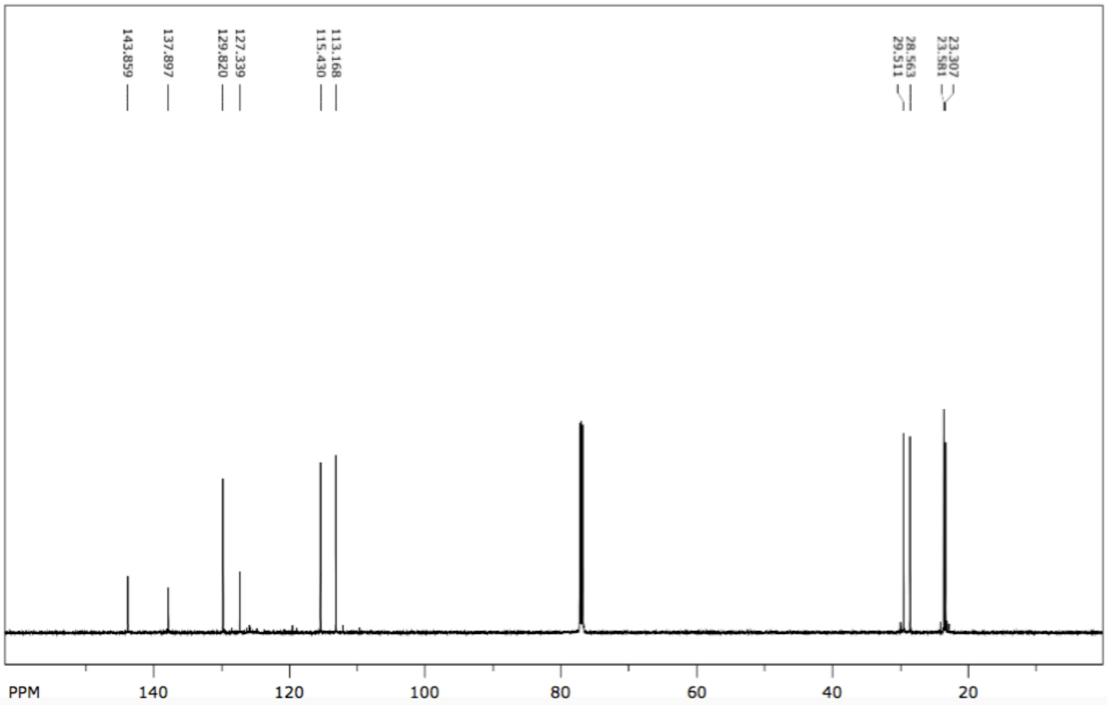

Figure S32. ^{1}H and ^{13}C NMR spectra of **2w**.

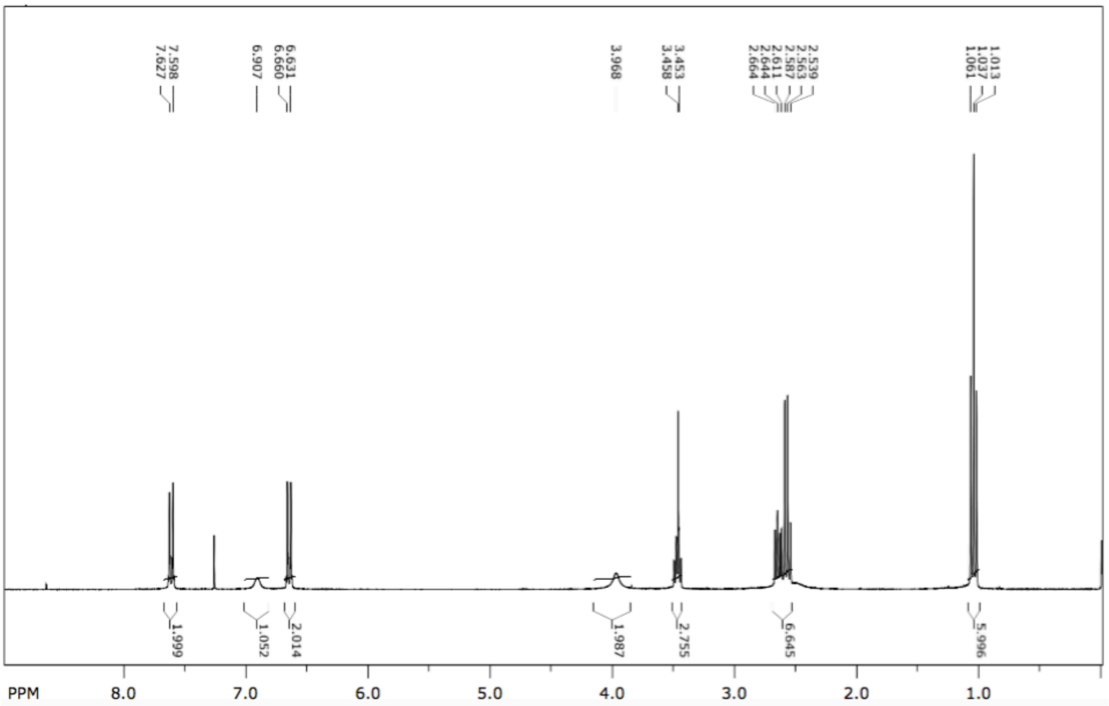

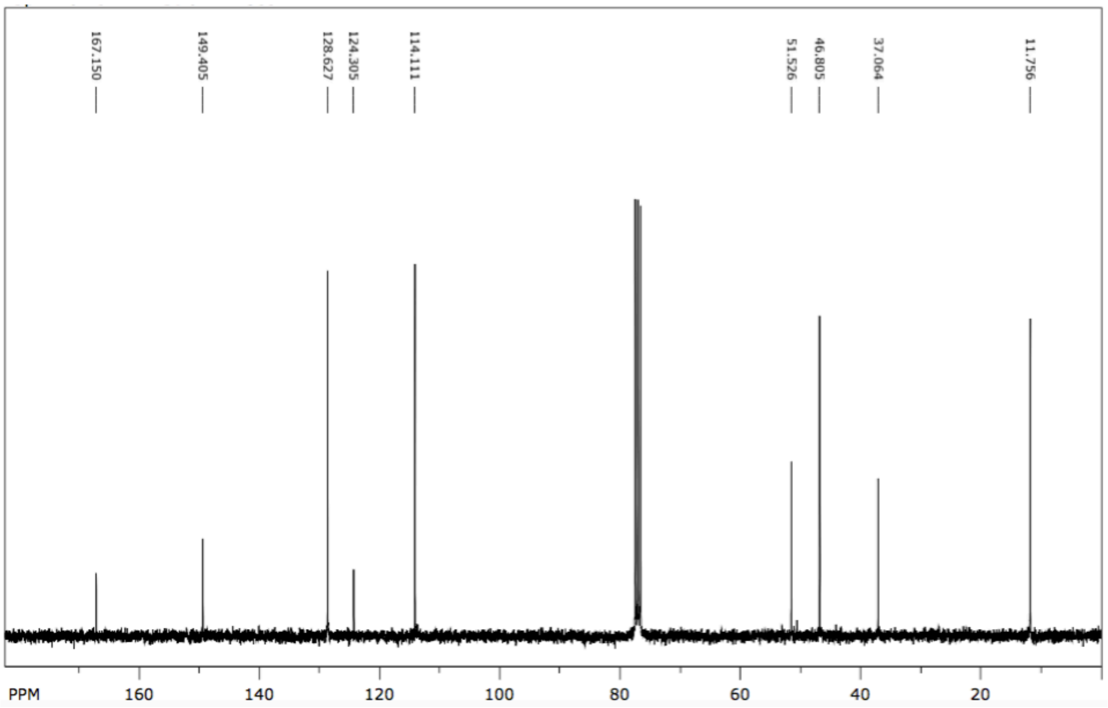

Figure S33. ^{1}H and ^{13}C NMR spectra of **5**. *Note: The CH_2 quartet signal at 3.46 ppm in ^{1}H NMR spectrum is overlapped with traces of methanol.*

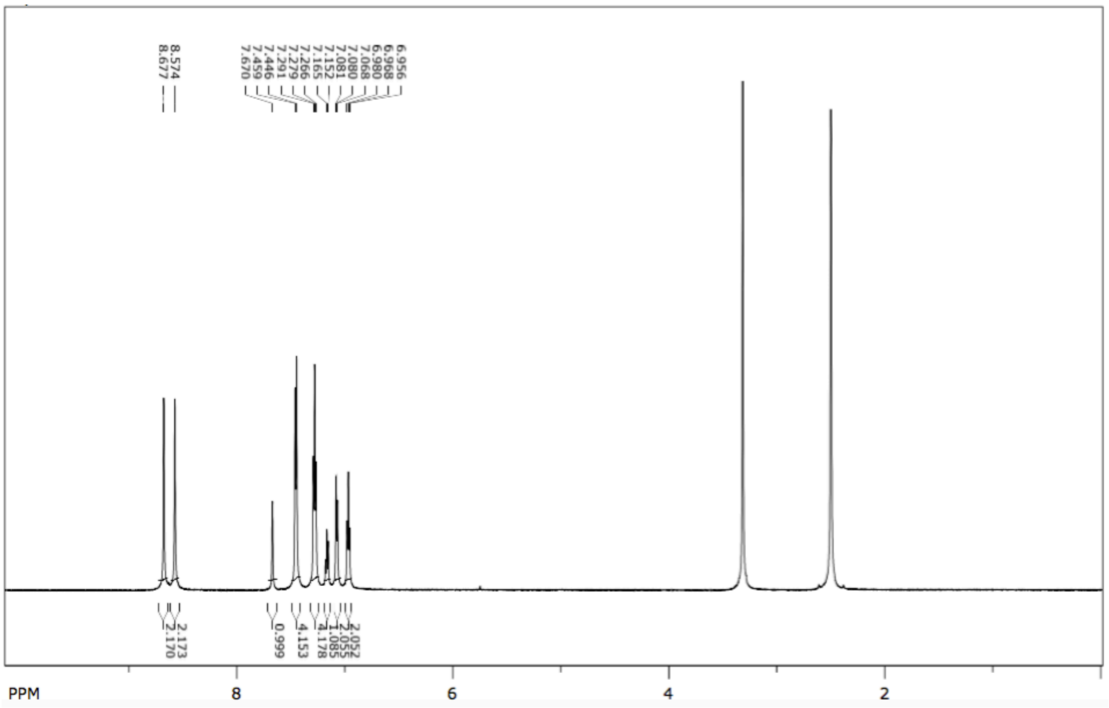

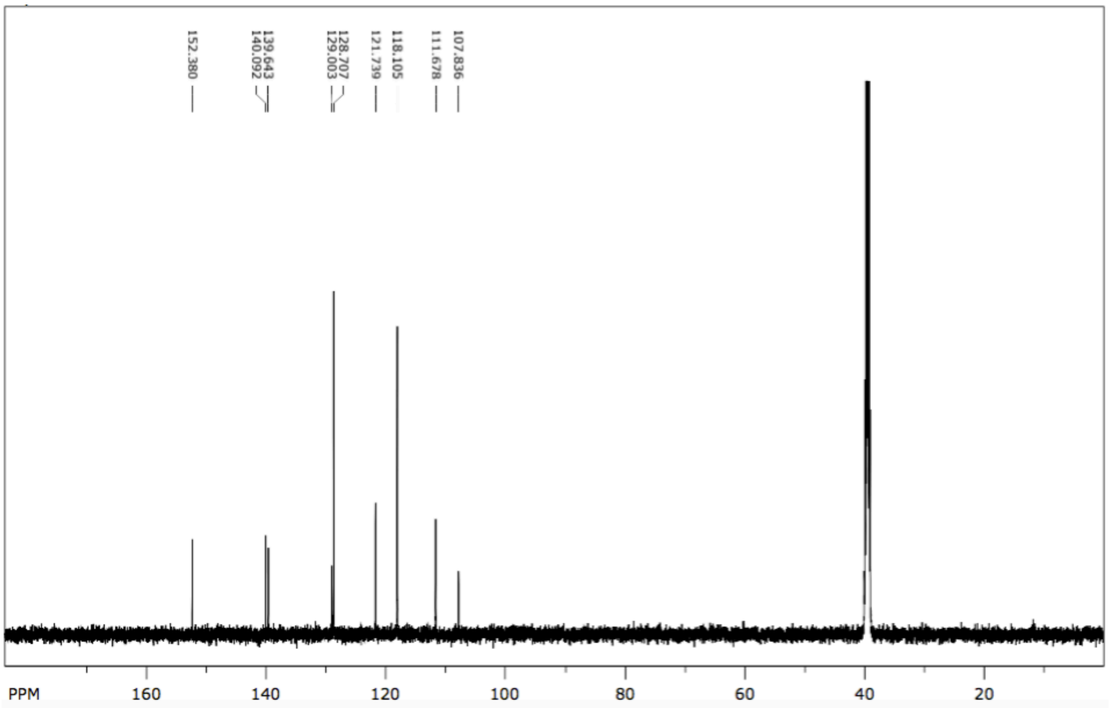

Figure S34. ^{1}H and ^{13}C NMR spectra of **7a**.

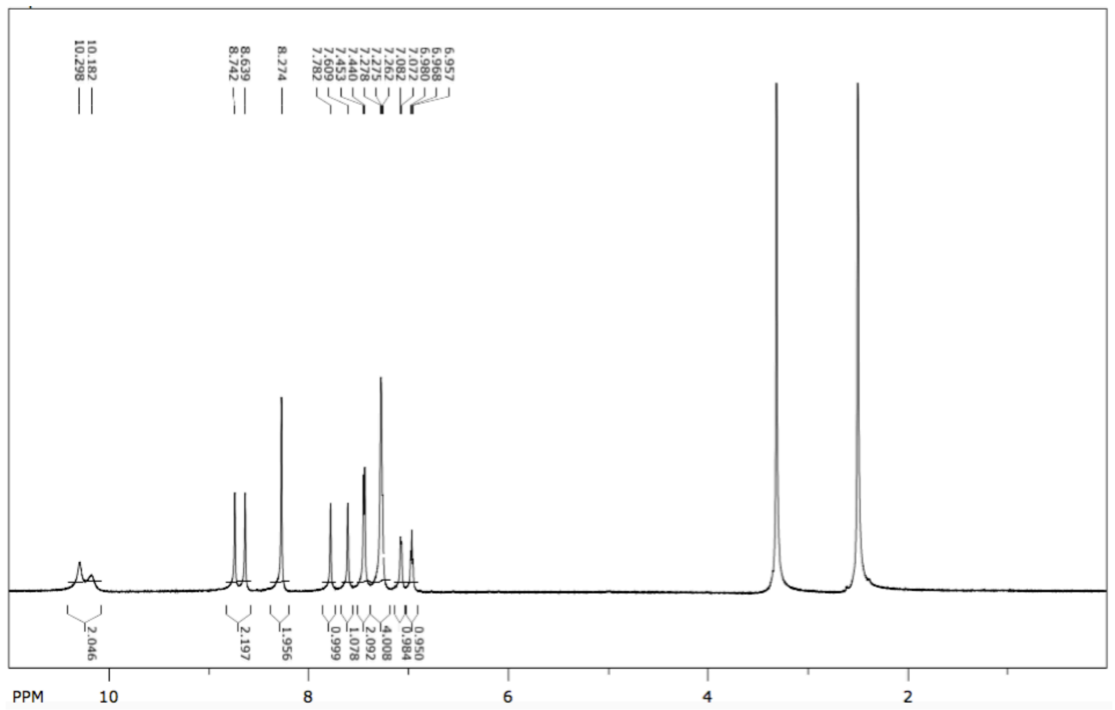

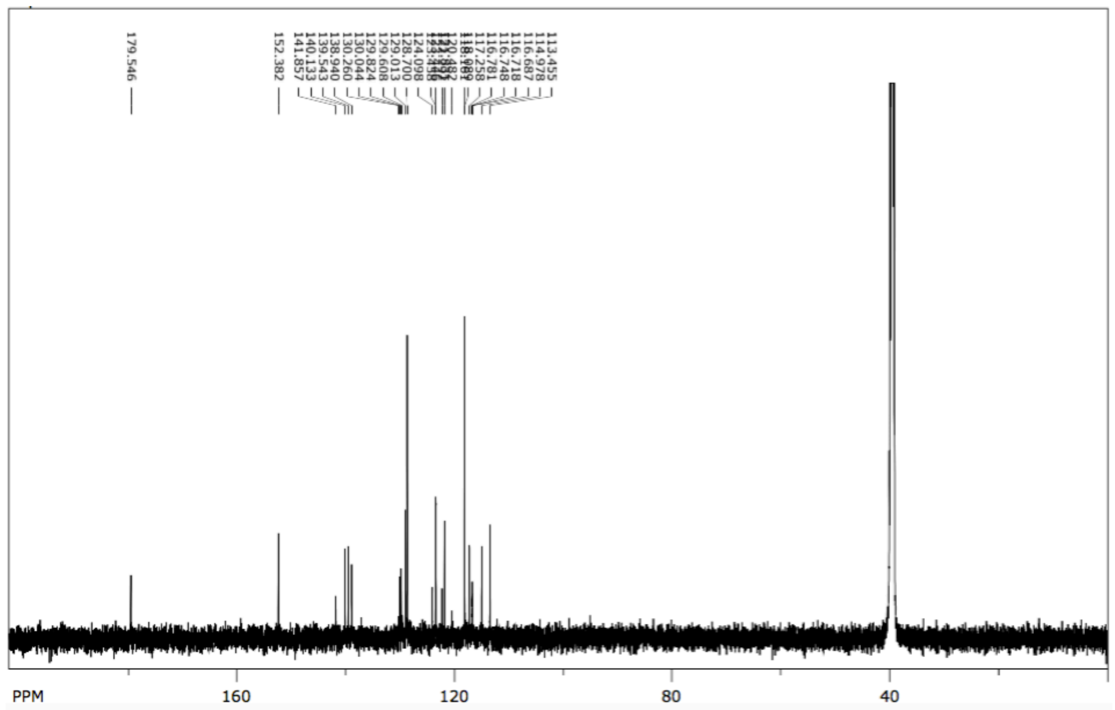

Figure S35. ^{1}H and ^{13}C NMR spectra of **7b**.

8. PXRD analysis of mechanochemically-synthesized paracetamol (6)

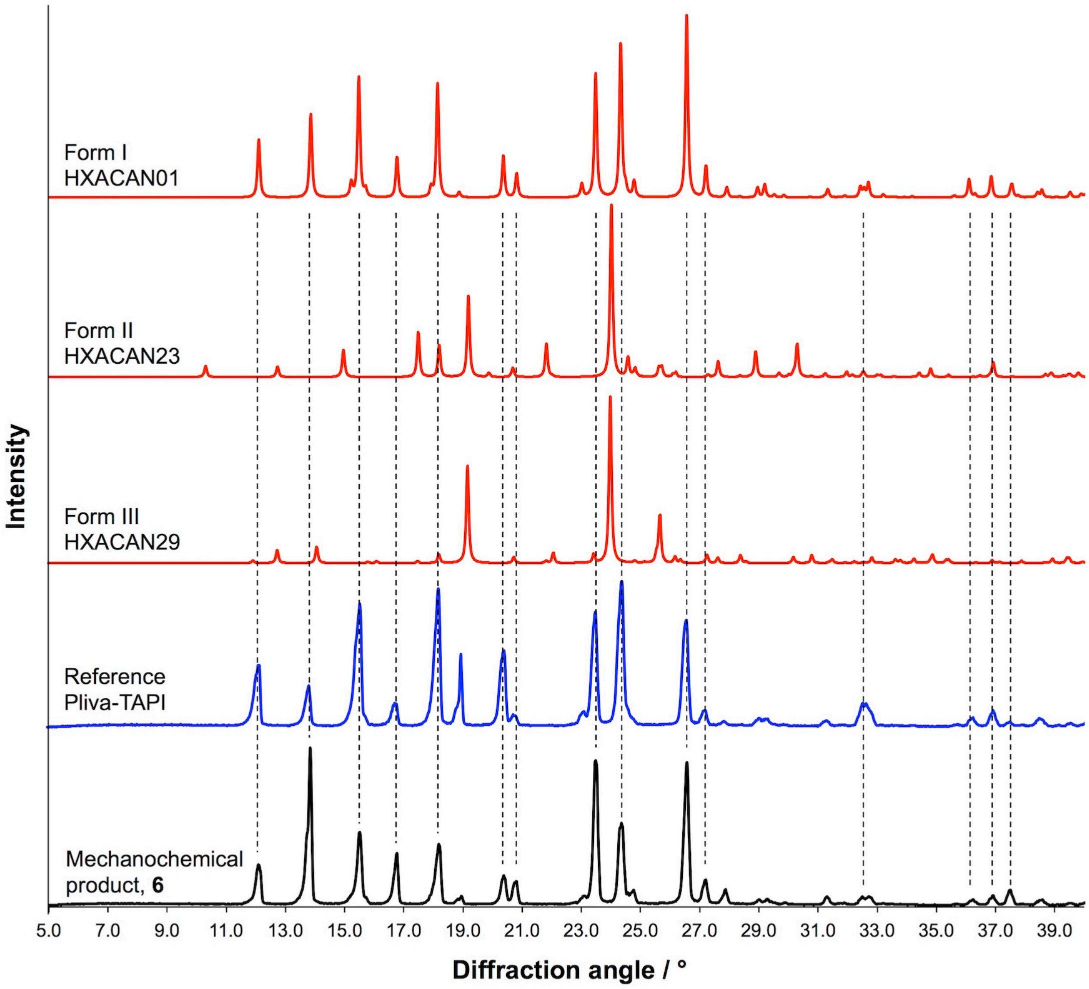

Figure S36. PXRD analysis shows that mechanochemically-synthesized paracetamol (**6**) adopts the crystal structure of the thermodynamically most stable polymorph, form I.

9. References

1. NMR data for the following compounds are consistent with the ones deposited in the Spectral Database for Organic Compounds SDB (https://sdbs.db.aist.go.jp/sdbs/cgi-bin/direct_frame_top.cgi) or on Sigma Aldrich website (https://www.sigmaaldrich.com/european-export.html).

3-aminobenzonitrile (2a) in d_6-DMSO, SDBS number: 21149

***o*-phenylenediamine (o-2b)** in d_6-DMSO, SDBS number: 2795

***p*-phenylenediamine (*p*-2b)** in $CDCl_3$, SDBS number: 1131

***o*-aminophenol (*o*-2c)** in d_6-DMSO, SDBS number: 1516

***m*-aminophenol (*m*-2c)** in d_6-DMSO, SDBS number: 1514

***p*-aminophenol (*p*-2c)** in d_6-DMSO, SDBS number: 1520

***p*-toluidine (2s)** in $CDCl_3$, SDBS number: 990

paracetamol (6) in d_6-DMSO, SDBS number: 3290

***o*-aminobenzoic acid (*o*-2d)** in d_6-DMSO, SDBS number: 1150

***p*-aminobenzoic acid (*p*-2d)** in d_6-DMSO, SDBS number: 1152

3,5-diaminobenzoic acid (2e) in d_6-DMSO,
https://www.sigmaaldrich.com/spectra/fnmr/FNMR009935.PDF

2-aminoterephthalic acid (2f) in d_6-DMSO,
https://www.sigmaaldrich.com/spectra/fnmr/FNMR008833.PDF

2. ***m*-phenylenediamine (*m*-2b)** in d_6-DMSO, in Su, Y.; Li, X.; Wang, Y.; Zhong, H.; Wang, R. Gold nanoparticles supported by imidazolium-based porous organic polymers for nitroarene reduction. *Dalton Trans.* **2016**, *45*, 16896–16903.

   ***m*-aminobenzoic acid (*m*-2d)** in d_6-DMSO, in Samsonowicz, M.; Hrynaszkiewicz, T.; Swisłocka, R.; Regulska, E.; Lewandowski, W. Experimental and theoretical IR, Raman, NMR spectra of 2-, 3- and 4-aminobenzoic acids. *J. Mol. Struct.* **2005**, *744–747*, 345–352.
